# Supplementary material for: Pennelliiside D, a New Acyl Glucose from Solanum pennellii and Chemical Synthesis of Pennelliisides
Source: Molecules. 2022 Jun 9;27(12):3728. doi: 10.3390/molecules27123728 (PMC9231340; doi:10.3390/molecules27123728)
Supplement: Supplementary file 1 [file molecules-27-03728-s001.zip › molecules-1751977-supplementary.pdf]

Supplementary Materials

Pennelliiside D, a new acyl glucose from *Solanum pennellii* and chemical synthesis of pennelliisides

Rishni Masimbula, Hiroto Kobayashi, Tenki Nakashima, Yurika Nambu, Naoki Kitaoka, and Hideyuki Matsuura*

Research Faculty of Agriculture, Hokkaido University, Kita 9 Nishi 9, Kita-ku, Sapporo 060-8589, Japan; rish.masimbula@gmail.com (R.M); hiryouryoukasai_36@eis.hokudai.ac.jp (H.K.); soccer.ikkyunyukon@gmail.com (T.N.); southpart1192@gmail.com (Y.N.); kitaoka@chem.agr.hokudai.ac.jp (N.K.); matsuura@chem.agr.hokudai.ac.jp (H.M.)

* Correspondence: matsuura@chem.agr.hokudai.ac.jp; Tel.: +81-11-706-2495

Table of content

**Scheme S1:** Synthesis of 1,6-*O*-dibenzyl-3,4-*O*-diisobutyryl-2-*O*-((*R*)-2-methylbutyryl)-*β*-D-glucose (**12**).	6
**Table S1**: ^{1}H NMR (500 MHz) and ^{13}C NMR (126 MHz) spectroscopic data of synthesized (*S*) and (*R*) isomers of dibenzyl pennelliiside D (**2**) in C_6D_6 (δ in ppm, *J* in Hz).	7
**Figure S1**: HRFD-MS spectrum of natural dibenzyl pennelliiside D (**2**).	8
**Figure S2**: ^{1}H NMR spectrum of natural dibenzyl pennelliiside D (**2**) (500 MHz, C_6D_6).	9
**Figure S3**: ^{13}C NMR spectrum of natural dibenzyl pennelliiside D (**2**) (126 MHz, C_6D_6).	10
**Figure S4**: COSY spectrum of natural dibenzyl pennelliiside D (**2**) (500 MHz, C_6D_6).	11
**Figure S5**: HSQC spectrum of natural dibenzyl pennelliiside D (**2**) (500 MHz, C_6D_6).	12
**Figure S6**: HMBC spectrum of natural dibenzyl pennelliiside D (**2**) (500 MHz, C_6D_6).	13
**Figure S7**: NOESY spectrum of natural dibenzyl pennelliiside D (**2**) (500 MHz, C_6D_6).	14
**Figure S8**: HRFD-MS spectrum of natural pennelliiside D (**1**).	15
**Figure S9**: ^{1}H NMR spectrum of natural pennelliiside D (**1**) (500 MHz, $CDCl_3$).	16
**Figure S10**: ^{13}C NMR spectrum of natural pennelliiside D (**1**) (126 MHz, $CDCl_3$).	17
**Figure S11**: COSY spectrum of natural pennelliiside D (**1**) (500 MHz, $CDCl_3$).	18
**Figure S12**: COSY spectrum of natural pennelliiside D (**1**) (500 MHz, $CDCl_3$).	19
**Figure S13**: HSQC spectrum of natural pennelliiside D (**1**) (500 MHz, $CDCl_3$).	20
**Figure S14**: HMBC spectrum of natural pennelliiside D (**1**) (500 MHz, $CDCl_3$).	21
**Figure S15**: HRFD-MS spectrum of **4**.	22
**Figure S16**: ^{1}H NMR spectrum of compound **4** (270 MHz, $CDCl_3$).	23
**Figure S17**: ^{13}C NMR spectrum of compound **4** (126 MHz, $CDCl_3$).	24
**Figure S18**: HRFD-MS spectrum of compound **5**.	25
**Figure S19**: ^{1}H NMR spectrum of compound **5** (270 MHz, CD_3OD).	26
**Figure S20**: ^{13}C NMR spectrum of compound **5** (126 MHz, CD_3OD).	27
**Figure S21**: HRFD-MS spectrum of compound **6**.	28
**Figure S22**: ^{1}H NMR spectrum of compound **6** (500 MHz, $CDCl_3$).	29
**Figure S23**: ^{13}C NMR spectrum of compound **6** (126 MHz, $CDCl_3$).	30

**Figure S24**: COSY spectrum of compound **6** (500 MHz, $CDCl_3$).	31
**Figure S25**: HSQC spectrum of compound **6** (500 MHz, $CDCl_3$).	32
**Figure S26**: HMBC spectrum of compound **6** (500 MHz, $CDCl_3$).	33
**Figure S27**: HRFD-MS spectrum of compound **7**.	34
**Figure S28**: ^{1}H NMR spectrum of compound **7** (270 MHz, C_6D_6).	35
**Figure S29**: ^{13}C NMR spectrum of compound **7** (126 MHz, C_6D_6).	36
**Figure S30**: HRFD-MS spectrum of compound **8**.	37
**Figure S31**: ^{1}H NMR spectrum of compound **8** (500 MHz, C_6D_6).	38
**Figure S32**: ^{13}C NMR spectrum of compound **8** (126 MHz, C_6D_6).	39
**Figure S33**: COSY spectrum of compound **8** (500 MHz, C_6D_6).	40
**Figure S34**: HSQC spectrum of compound **8** (500 MHz, C_6D_6).	41
**Figure S35**: HMBC spectrum of compound **8** (500 MHz, C_6D_6).	42
**Figure S36**: NOESY spectrum of compound **8** (500 MHz, C_6D_6).	43
**Figure S37**: HRFD-MS spectrum of compound **9**.	44
**Figure S38**: ^{1}H NMR spectrum of compound **9** (500 MHz, C_6D_6).	45
**Figure S39**: ^{13}C NMR spectrum of compound **9** (126 MHz, C_6D_6).	46
**Figure S40**: COSY spectrum of compound **9** (500 MHz, C_6D_6)..	47
**Figure S41**: HSQC spectrum of compound **9** (500 MHz, C_6D_6).	48
**Figure S42**: HMBC spectrum of compound **9** (500 MHz, C_6D_6).	49
**Figure S43**: NOESY spectrum of compound **9** (500 MHz, C_6D_6).	50
**Figure S44**: HRFD-MS spectrum of synthesized dibenzyl pennelliiside D (**2**).	51
**Figure S45**: ^{1}H NMR spectrum of synthesized dibenzyl pennelliiside D (**2**) (500 MHz, C_6D_6).	52
**Figure S46**: ^{13}C NMR spectrum of synthesized dibenzyl pennelliiside D (**2**) (126 MHz, C_6D_6).	53
**Figure S47**: COSY spectrum of synthesized dibenzyl pennelliiside D (**2**) (500 MHz, C_6D_6).	54
**Figure S48**: HSQC spectrum of synthesized dibenzyl pennelliiside D (**2**) (500 MHz, C_6D_6).	55
**Figure S49**: HMBC spectrum of synthesized dibenzyl pennelliiside D (**2**) (500 MHz, C_6D_6).	56
**Figure S50**: NOESY spectrum of synthesized dibenzyl pennelliiside D (**2**) (500 MHz, C_6D_6).	57
**Figure S51**: HRFD-MS spectrum of synthesized pennelliiside D (**1**).	58

**Figure S52**: ^{1}H NMR spectrum of synthesized pennelliiside D (**1**) (500 MHz, $CDCl_3$).	59
**Figure S53**: ^{13}C NMR spectrum of synthesized pennelliiside D (**1**) (126 MHz, $CDCl_3$).	60
**Figure S54**: COSY spectrum of synthesized pennelliiside D (**1**) (500 MHz, $CDCl_3$).	61
**Figure S55**: COSY spectrum of synthesized pennelliiside D (**1**) (500 MHz, $CDCl_3$).	62
**Figure S56**: HSQC spectrum of synthesized pennelliiside D (**1**) (500 MHz, $CDCl_3$).	63
**Figure S57**: HMBC spectrum of synthesized pennelliiside D (**1**) (500 MHz, $CDCl_3$).	64
**Figure S58**: ^{1}H NMR spectra of natural and synthesized pennelliiside D (**1**) (500 MHz, $CDCl_3$).	65
**Figure S59**: ^{13}C NMR spectrum of natural and synthesized pennelliiside D (**1**) (126 MHz, $CDCl_3$).	66
**Figure S60**: HRFD-MS spectrum of compound **10**.	67
**Figure S61**: ^{1}H NMR spectrum of compound **10** (500 MHz, C_6D_6).	68
**Figure S62**: ^{13}C NMR spectrum of compound **10** (126 MHz, C_6D_6).	69
**Figure S63**: COSY spectrum of compound **10** (500 MHz, C_6D_6).	70
**Figure S64**: HSQC spectrum of compound **10** (500 MHz, C_6D_6).	71
**Figure S65**: HMBC spectrum of compound **10** (500 MHz, C_6D_6).	72
**Figure S66**: HRFD-MS spectrum of compound **11**.	73
**Figure S67**: ^{1}H NMR spectrum of compound **11** (270 MHz, C_6D_6).	74
**Figure S68**: ^{13}C NMR spectrum of compound **11** (126 MHz, C_6D_6).	75
**Figure S69**: HRFD-MS spectrum of compound **12**.	76
**Figure S70**: ^{1}H NMR spectrum of compound **12** (500 MHz, C_6D_6).	77
**Figure S71**: ^{13}C NMR spectrum of compound **12** (126 MHz, C_6D_6).	78
**Figure S72**: COSY spectrum of compound **12** (500 MHz, C_6D_6).	79
**Figure S73**: HSQC spectrum compound **12** (500 MHz, C_6D_6).	80
**Figure S74**: HMBC spectrum of compound **12** (500 MHz, C_6D_6).	81
**Figure S75**: NOESY spectrum of compound **12** (500 MHz, C_6D_6).	82

**Figure S76**: HRFD-MS spectrum of compound **13**.	83
**Figure S77**: ^{1}H NMR spectra of compound **13** (500 MHz, C_6D_6).	84
**Figure S78**: ^{13}C NMR spectrum of compound **13** (126 MHz, C_6D_6).	85
**Figure S79**: HRFD-MS spectrum of compound **16a**.	86
**Figure S80**: ^{1}H NMR spectra of compound **16a** (500 MHz, C_6D_6).	87
**Figure S81**: ^{13}C NMR spectrum of compound **16a** (126 MHz, C_6D_6).	88
**Figure S82**: HRFD-MS spectrum of compound **17a**.	89
**Figure S83**: ^{1}H NMR spectra of compound **17a** (500 MHz, C_6D_6).	90
**Figure S84**: ^{13}C NMR spectrum of compound **17a** (126 MHz, C_6D_6).	91
**Figure S85**: COSY spectrum of compound **17a** (500 MHz, C_6D_6).	92
**Figure S86**: HSQC spectrum of compound **17a** (500 MHz, C_6D_6).	93
**Figure S87**: HMBC spectrum of compound **17a** (500 MHz, C_6D_6).	94
**Figure S88**: HRFD-MS spectrum of compound **14**.	95
**Figure S89**: ^{1}H NMR spectra of compound **14** (500 MHz, $CDCl_3$).	96
**Figure S90**: ^{13}C NMR spectrum of compound **14** (126 MHz, C_6D_6).	97
**Figure S91**: HRFD-MS spectrum of compound **15**.	98
**Figure S92**: ^{1}H NMR spectra of compound **15** (500 MHz, $CDCl_3$).	99
**Figure S93**: ^{13}C NMR spectrum of compound **15** (126 MHz, $CDCl_3$).	100
**Figure S94**: HRFD-MS spectrum of compound **16b**.	101
**Figure S95**: ^{1}H NMR spectrum of compound **16b** (500 MHz, C_6D_6).	102
**Figure S96**: ^{13}C NMR spectrum of compound **16b** (126 MHz, C_6D_6).	103
**Figure S97**: HRFD-MS spectrum of compound **17b**.	104
**Figure S98**: ^{1}H NMR spectra of compound **17b** (500 MHz, C_6D_6).	105
**Figure S99**: ^{13}C NMR spectrum of compound **17b** (126 MHz, C_6D_6).	106
**Figure S100**: COSY spectrum of compound **17b** (500 MHz, C_6D_6).	107
**Figure S101**: HSQC spectrum compound **17b** (500 MHz, C_6D_6).	108
**Figure S102**: HMBC spectrum of compound **17b** (500 MHz, C_6D_6).	109

Scheme S1: Synthesis of 1,6-*O*-dibenzyl-3,4-*O*-diisobutyryl-2-*O*-((*R*)-2-methylbutyryl)-*β*-D-glucose (**12**).

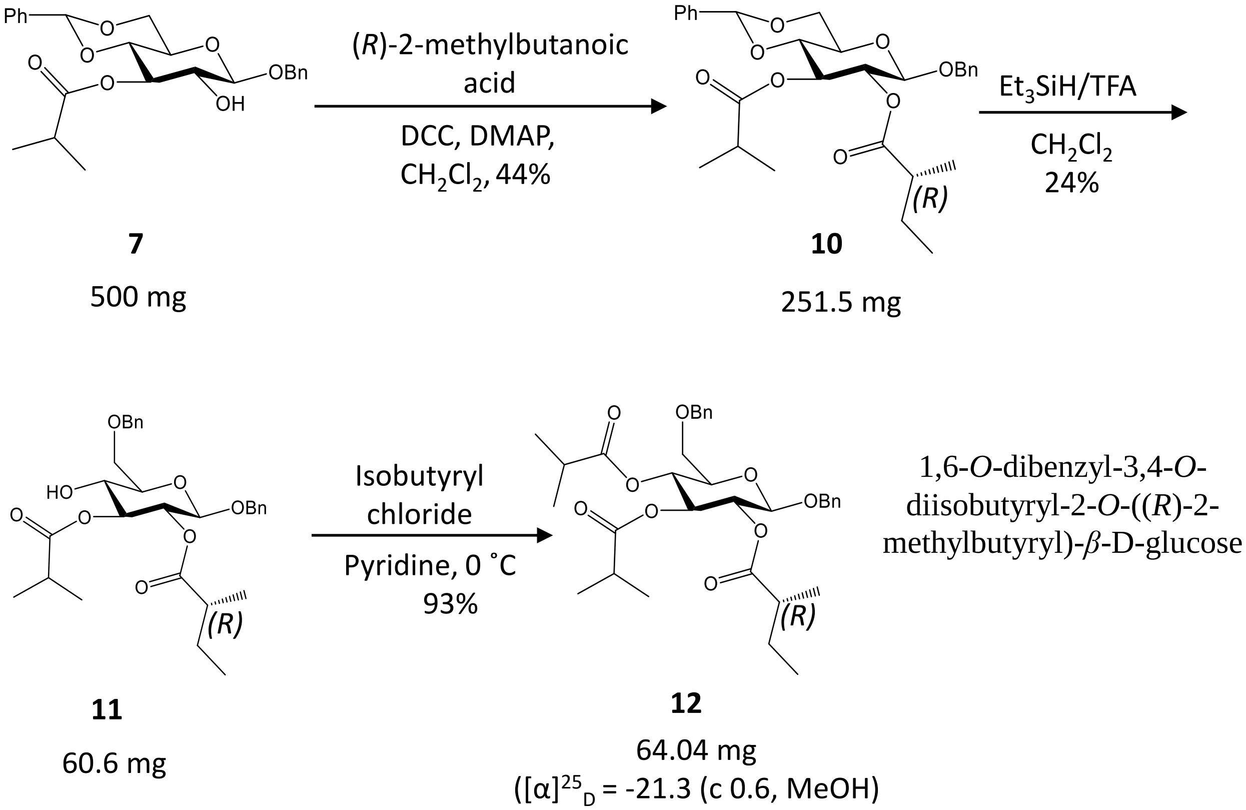

Synthesis procedures were similar to the synthesis of compound **8**, **9**, and **2**. Here, (*R*)-2-methylbutanoic acid was used instead of (*S*)-2-methylbutanoic acid in scheme 2.

Table S1: ^{1}H NMR (500 MHz) and ^{13}C NMR (126 MHz) spectroscopic data of synthesized (*S*) and (*R*) isomers of dibenzyl pennelliiside D (**2**) in C_6D_6 (δ in ppm, *J* in Hz).

Position	Type	Synthesized (*S*) isomer of dibenzyl pennelliiside D (2)		Synthesized (*R*) isomer of dibenzyl pennelliiside D (2)	
		δ_C	δ_H (*J* in Hz)	δ_C	δ_H (*J* in Hz)
1	CH	100.3	4.38, d (7.6)	100.2	4.38, d (7.9)
2	CH	71.8	5.46, m	71.9	5.45, m
3	CH	73.5	5.48, m	73.6	5.47, m
4	CH	70.1	5.30, dd (9.6, 9.5)	70.0	5.30, dd (9.8, 9.5)
5	CH	74.3	3.42, m	74.3	3.42, m
6	CH_2	69.9	3.47, m	69.9	3.47, m
1a'	CH_2	70.7	4.75, d (12.2)	70.7	4.75, d (12.2)
1b'			4.45, d (12.2)		4.45, d (12.2)
2'	C	138.01		138.0	
3'	CH	128.1-128.6	7.26, t (7.3)	128.1-128.7	7.26, t (7.3)
4'	CH	128.1-128.6	7.12-7.19, m	128.1-128.7	7.12-7.19, m
5'	CH	128.1-128.6	7.08, t (7.3)	128.1-128.7	7.08, t (7.0)
6'	CH	128.1-128.6	7.12-7.19, m	128.1-128.7	7.12-7.19, m
7'	CH	128.1-128.6	7.26, t (7.3)	128.1-128.7	7.26, t (7.3)
A1	C	174.7		174.9	
A2	CH	41.9	2.28 m	41.6	2.29, m
A3	CH_3	17.2	1.08, d (7.1)	17.1-19.6	1.07, d (7.1)
A4	CH_2	27.2	1.32, 1.69, m, m	27.3	1.33, 1.68, m, m
A5	CH_3	12.2	0.81, t (7.4)	12.1	0.81, t (7.4)
B1	C	176.2		175.3	
B2	CH	34.6	2.41, m	34.5	2.41, m
B3	CH_3	19.2-19.5	1.08, d (7.0)	17.1-19.6	1.07, d (7.1)
B4	CH_3	19.2-19.5	1.08, d (7.0)	17.1-19.6	1.07, d (7.1)
C1	C	175.3		175.3	
C2	CH	34.5	2.31, m	34.5	2.30, m
C3	CH_3	19.2-19.5	1.02, d (7.0)	17.1-19.6	1.02, d (7.0)
C4	CH_3	19.2-19.5	0.98, d (7.0)	17.1-19.6	0.98, d (7.0)
1a''	CH_2	73.9	4.33, d (5.5)	73.9	4.33, d (5.4)
1b''			4.33, d (5.5)		4.33, d (5.4)
2''	C	139.0		139.0	
3''	CH	128.1-128.7	7.26, t (7.3)	128.1-128.7	7.26, t (7.3)
4''	CH	128.1-128.7	7.12-7.19, m	128.1-128.7	7.12-7.19, m
5''	CH	128.1-128.7	7.08, t (7.3)	128.1-128.7	7.08, t (7.0)
6''	CH	128.1-128.7	7.12-7.19, m	128.1-128.7	7.12-7.19, m
7''	CH	128.1-128.7	7.26, t (7.3)	128.1-128.7	7.26, t (7.3)

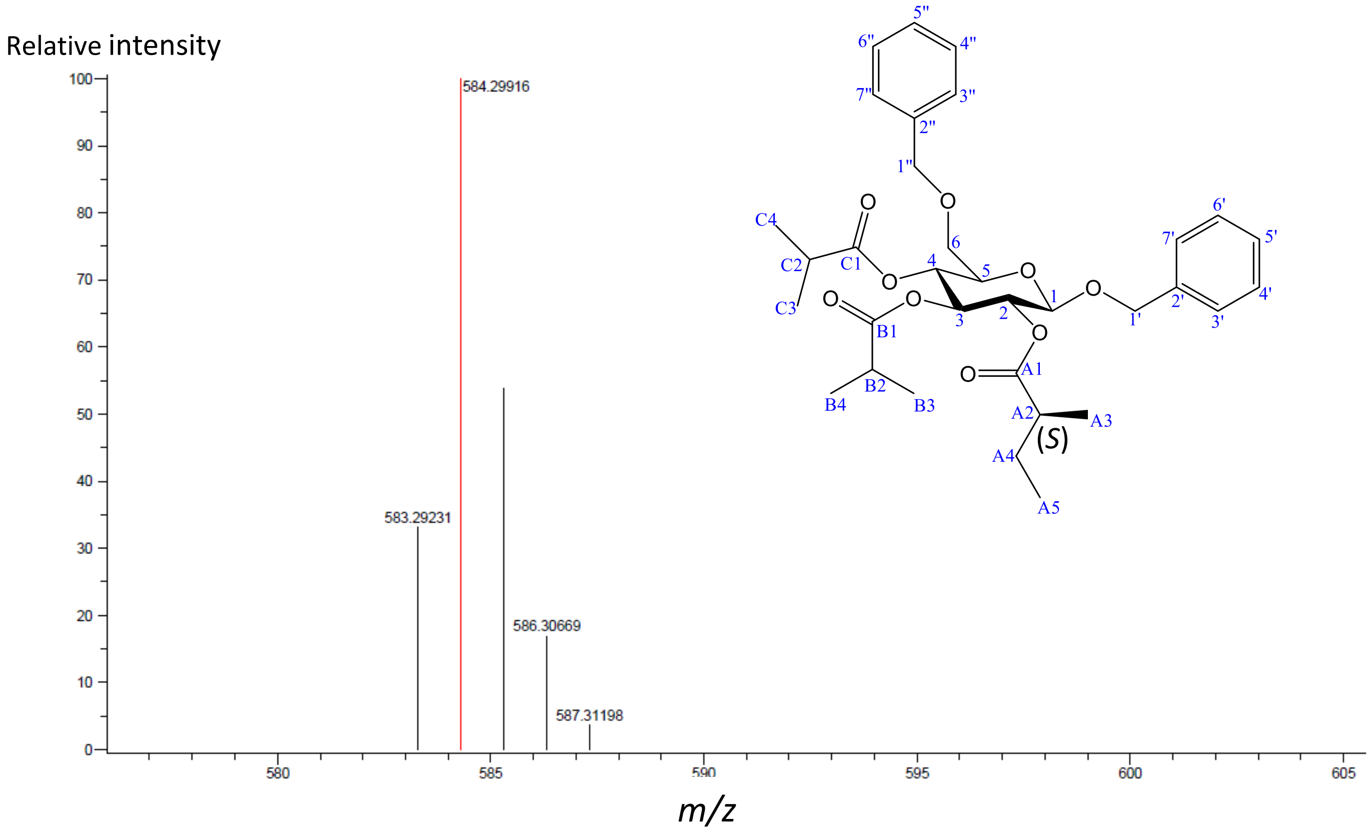

Figure S1: HRFD-MS spectrum of natural dibenzyl pennelliiside D (**2**).

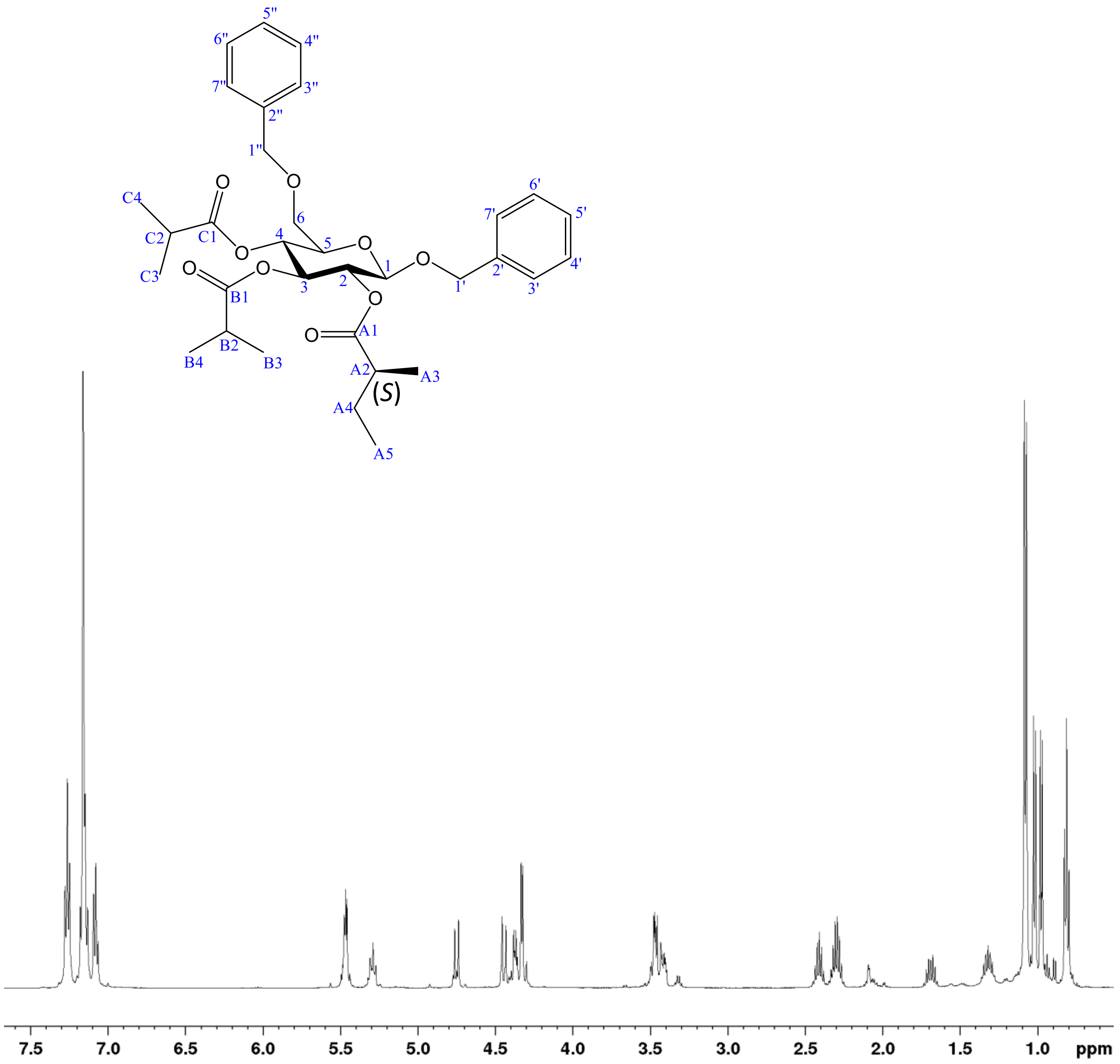

Figure S2: ^{1}H NMR spectrum of natural dibenzyl pennelliiside D (**2**) (500 MHz, C_6D_6).

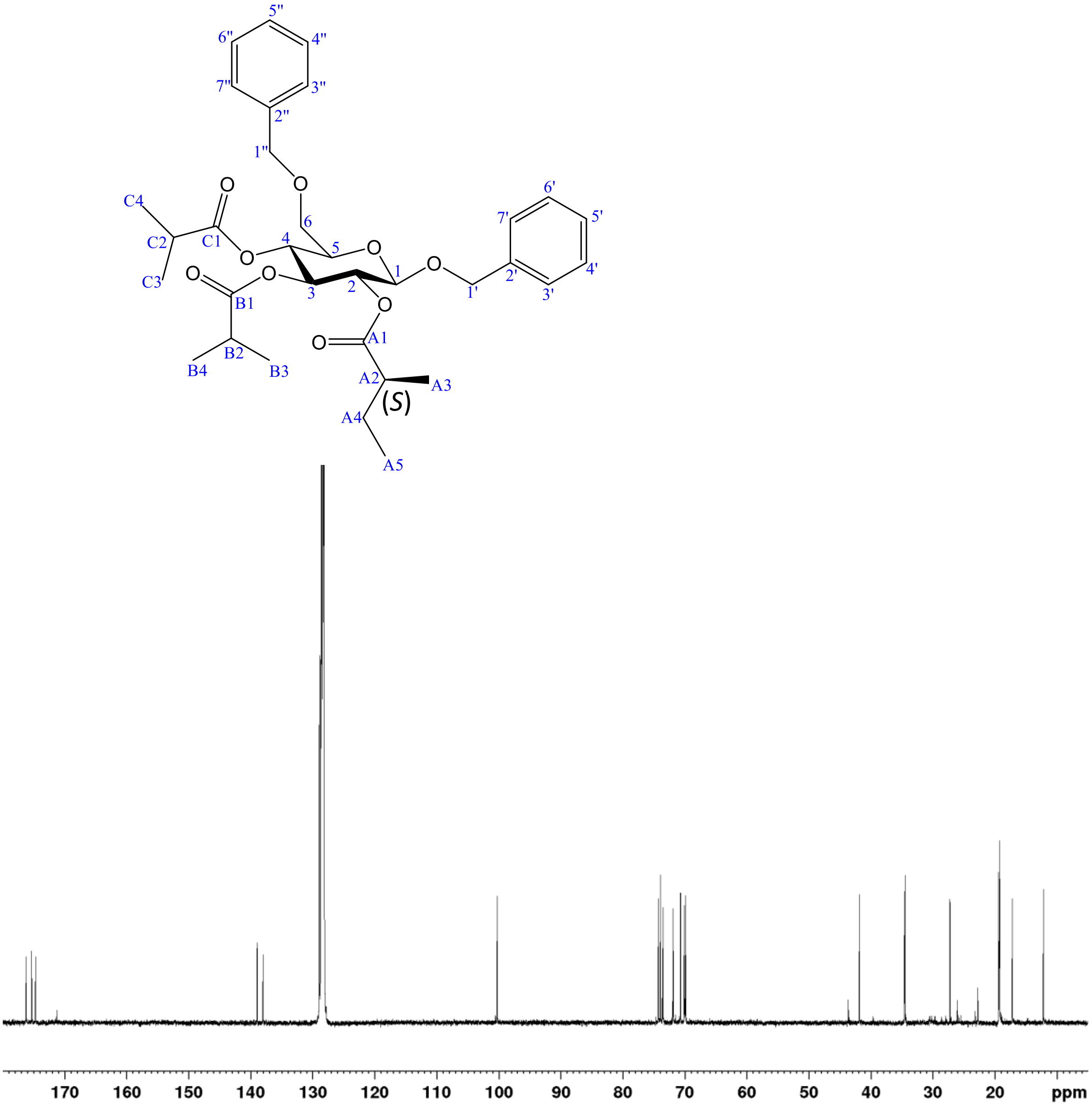

Figure S3: ^{13}C NMR spectrum of natural dibenzyl pennelliiside D (**2**) (126 MHz, C_6D_6).

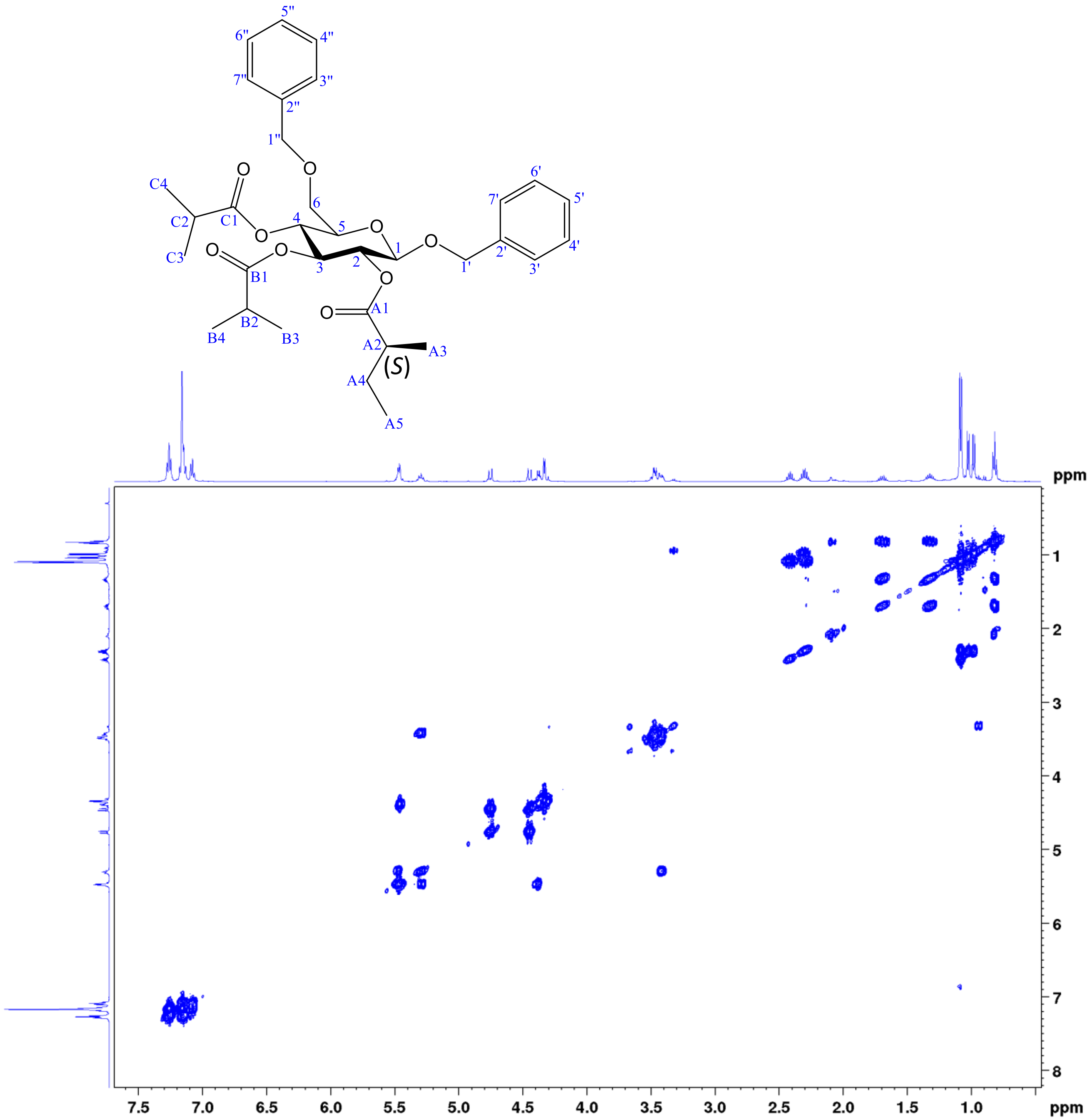

Figure S4: COSY spectrum of natural dibenzyl pennelliiside D (**2**) (500 MHz, C_6D_6).

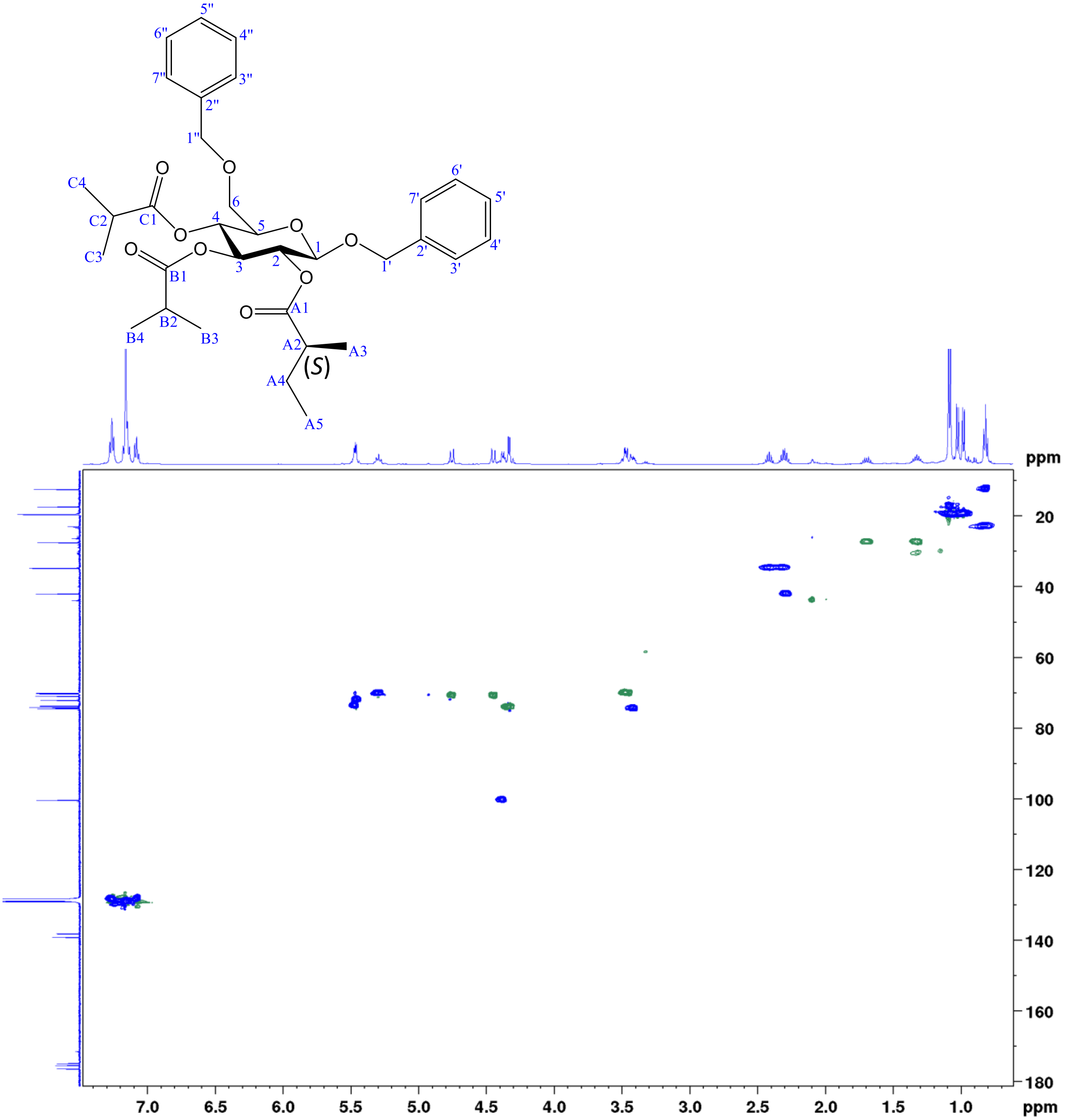

Figure S5: HSQC spectrum of natural dibenzyl pennelliiside D (**2**) (500 MHz, C_6D_6).

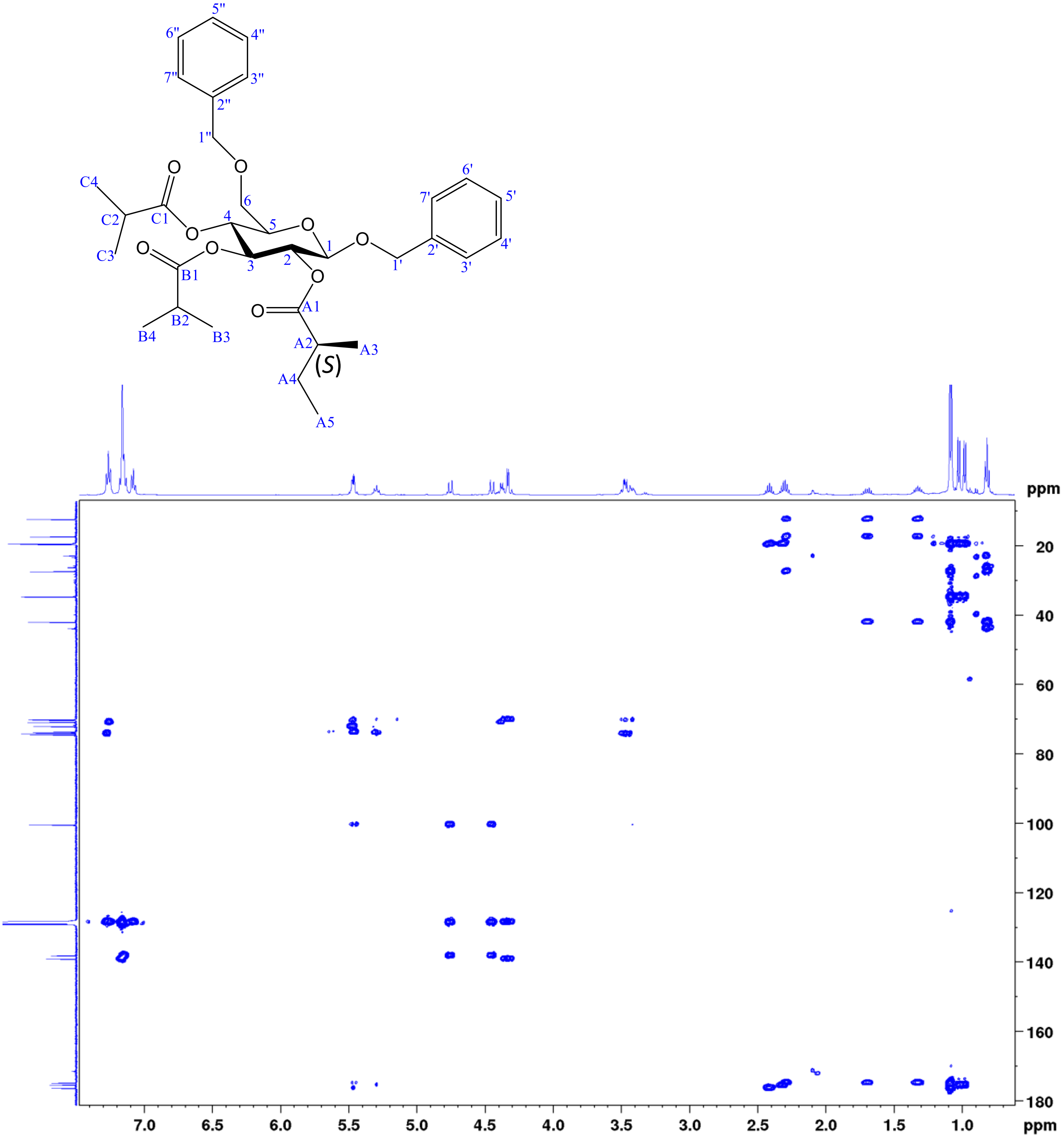

Figure S6: HMBC spectrum of natural dibenzyl pennelliiside D (**2**) (500 MHz, C_6D_6).

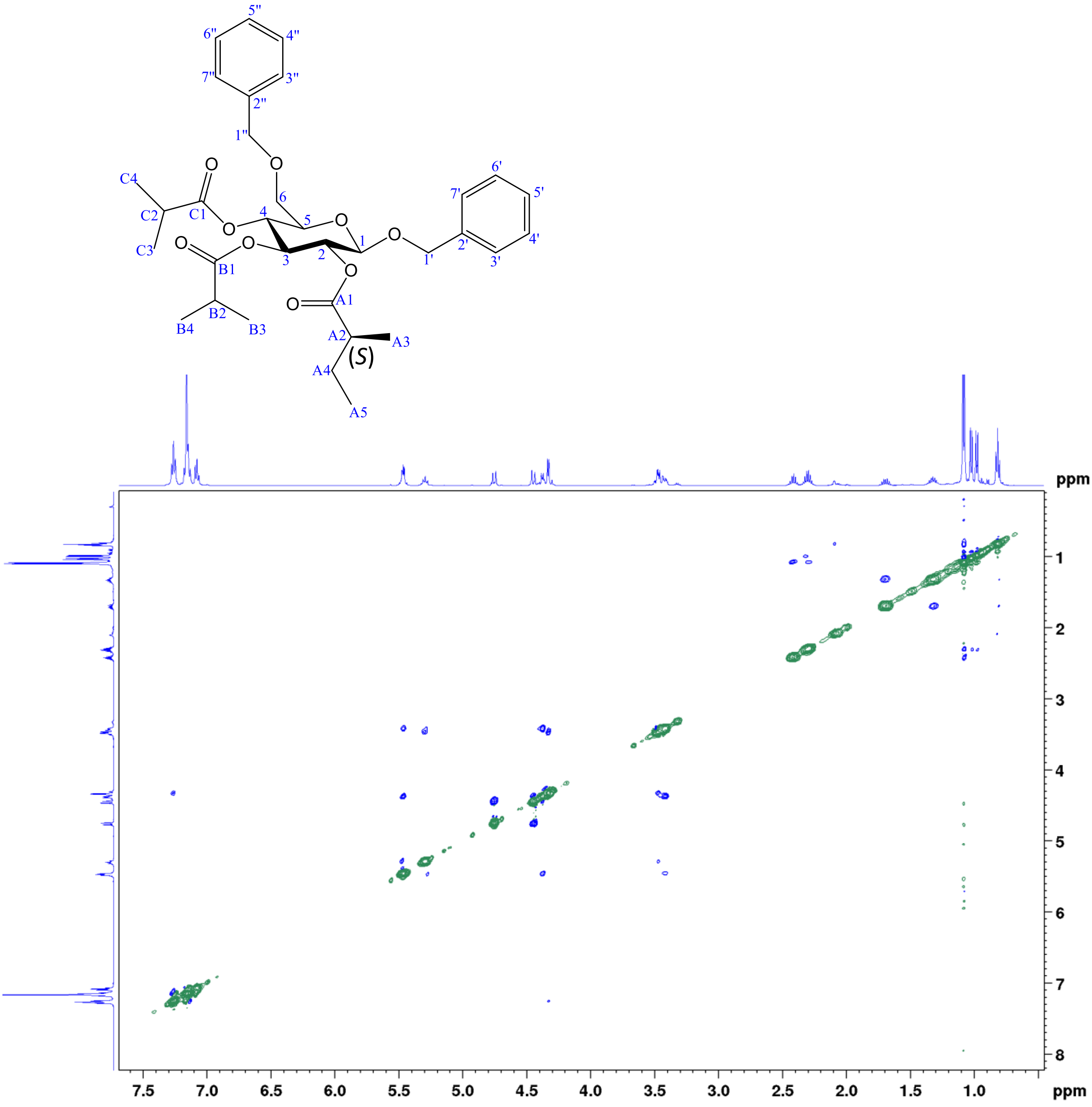

Figure S7: NOESY spectrum of natural dibenzyl pennelliiside D (**2**) (500 MHz, C_6D_6).

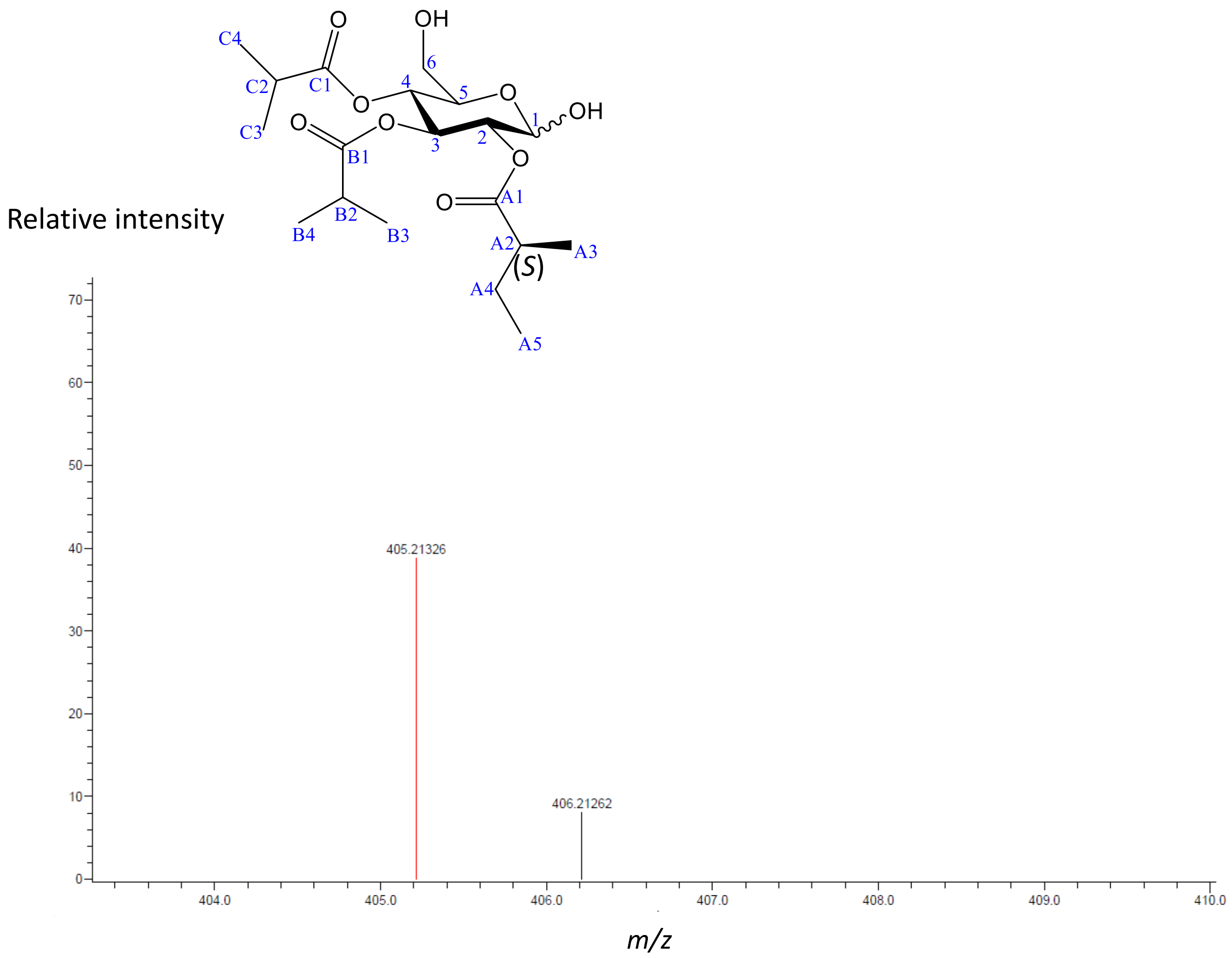

Figure S8: HRFD-MS spectrum of natural pennelliiside D (**1**).

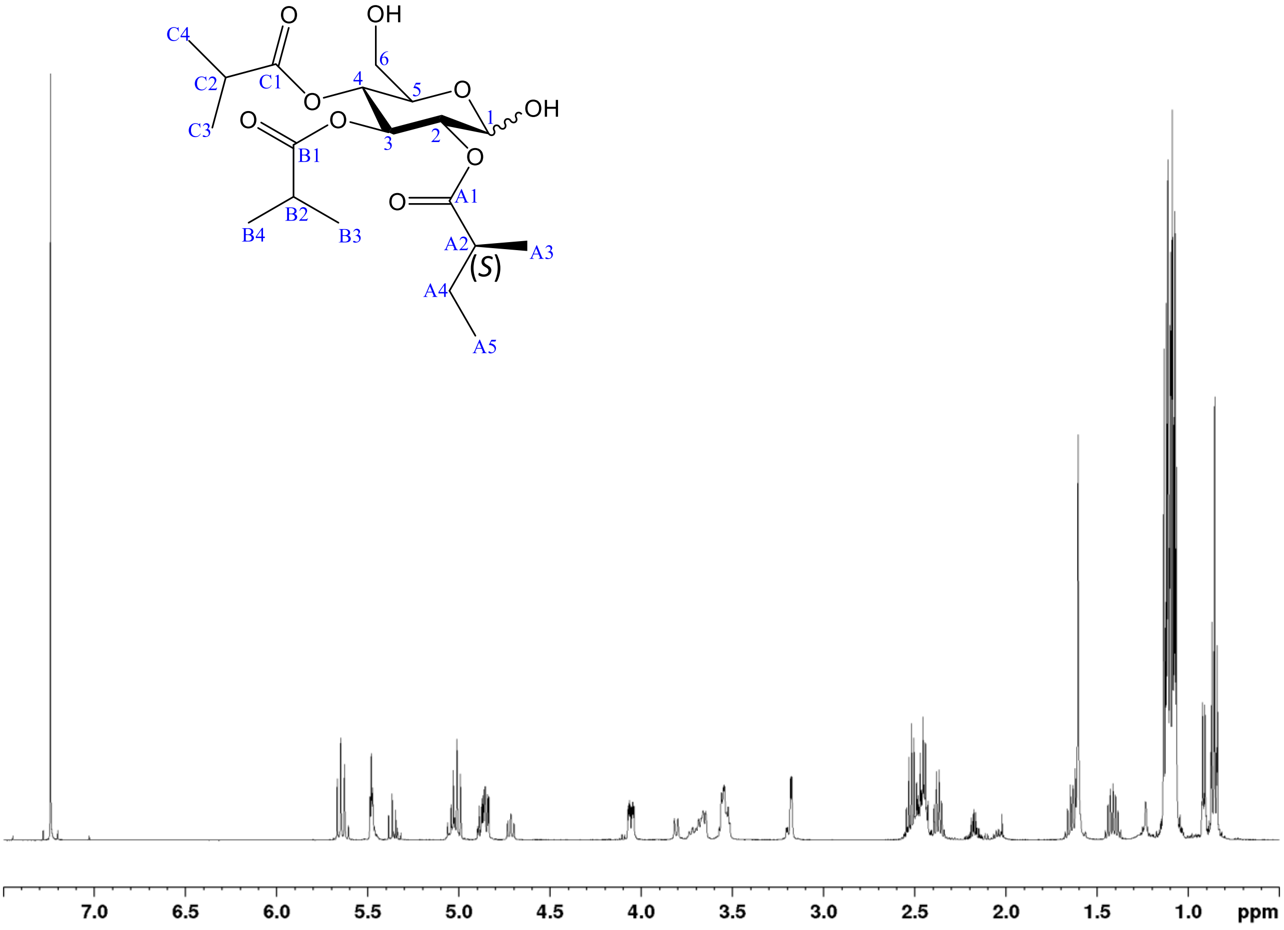

Figure S9: ^{1}H NMR spectrum of natural pennelliiside D (**1**) (500 MHz, $CDCl_3$).

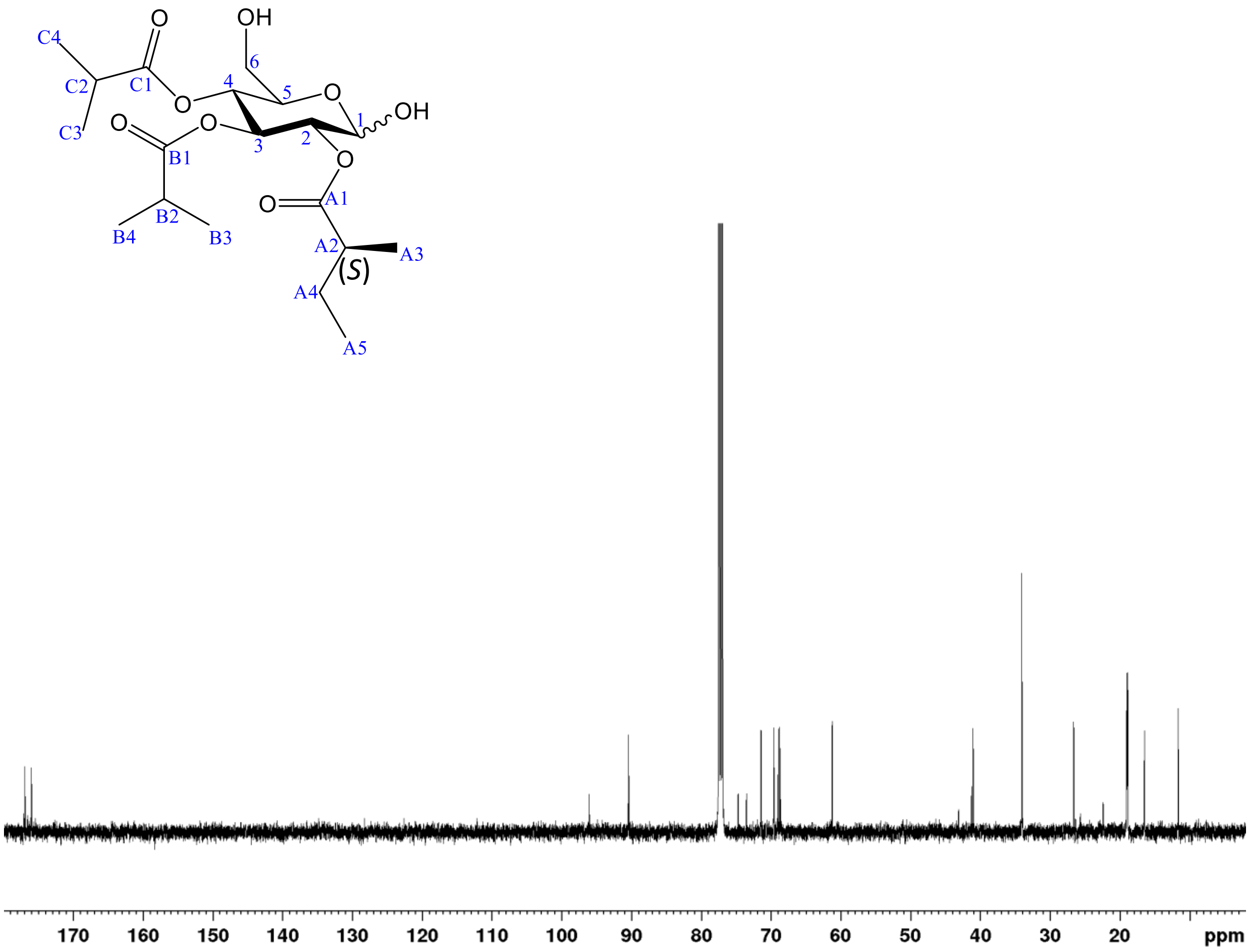

Figure S10: ^{13}C NMR spectrum of natural pennelliiside D (**1**) (126 MHz, $CDCl_3$).

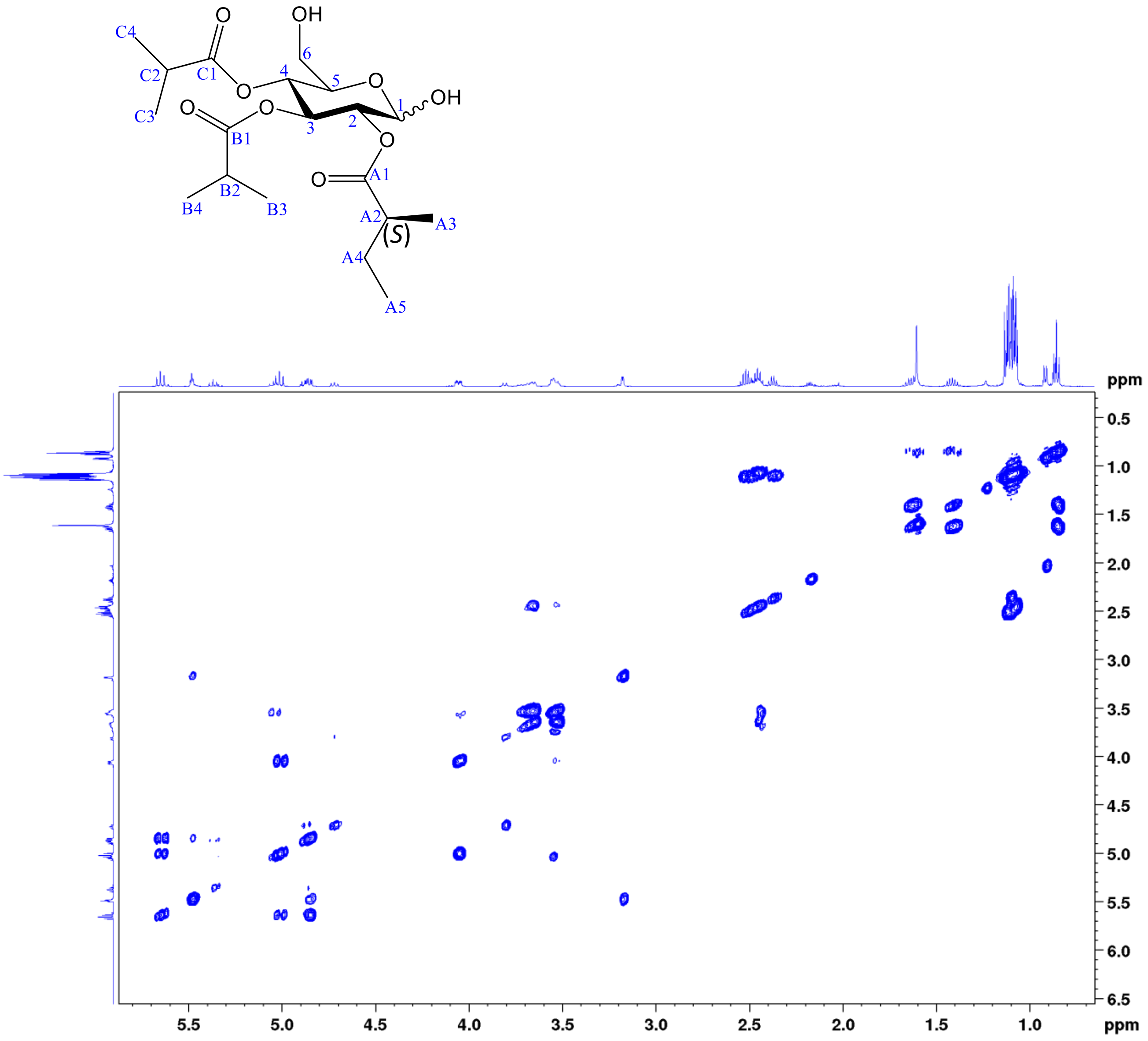

Figure S11: COSY spectrum of natural pennelliiside D (**1**) (500 MHz, $CDCl_3$).

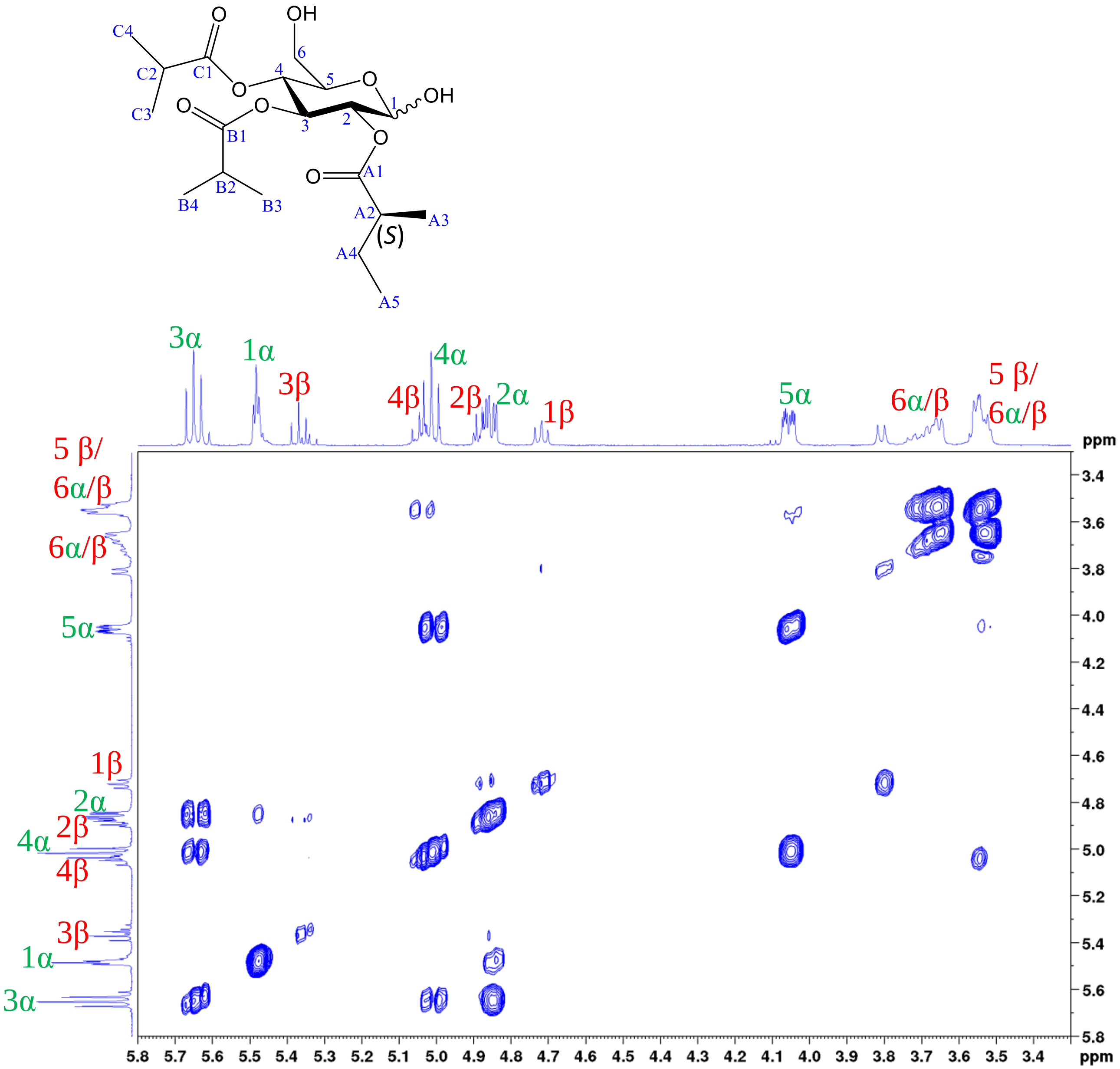

Figure S12: COSY spectrum of natural pennelliiside D (**1**) (500 MHz, $CDCl_3$).

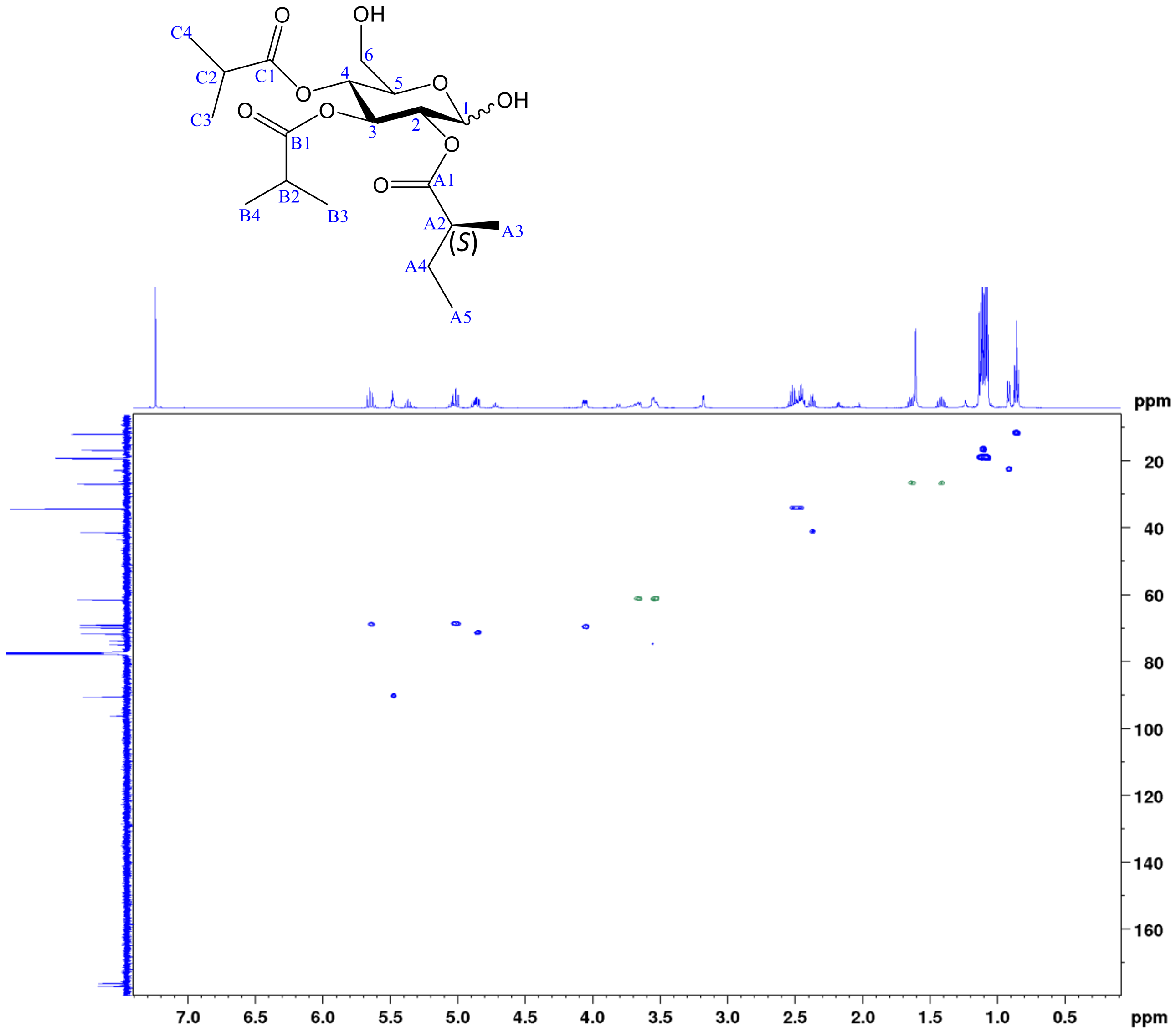

Figure S13: HSQC spectrum of natural pennelliiside D (**1**) (500 MHz, $CDCl_3$).

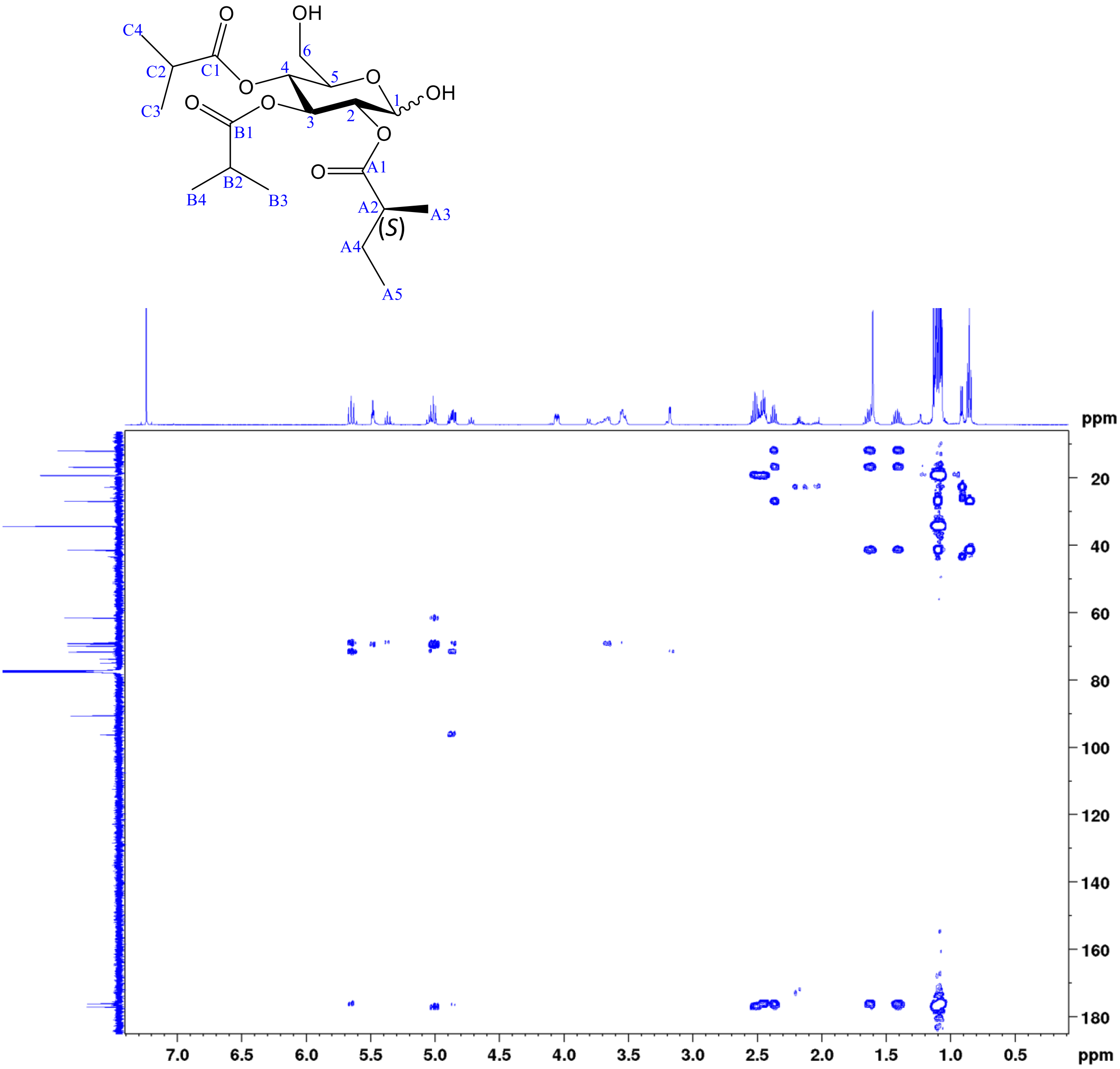

Figure S14: HMBC spectrum of natural pennelliiside D (**1**) (500 MHz, $CDCl_3$).

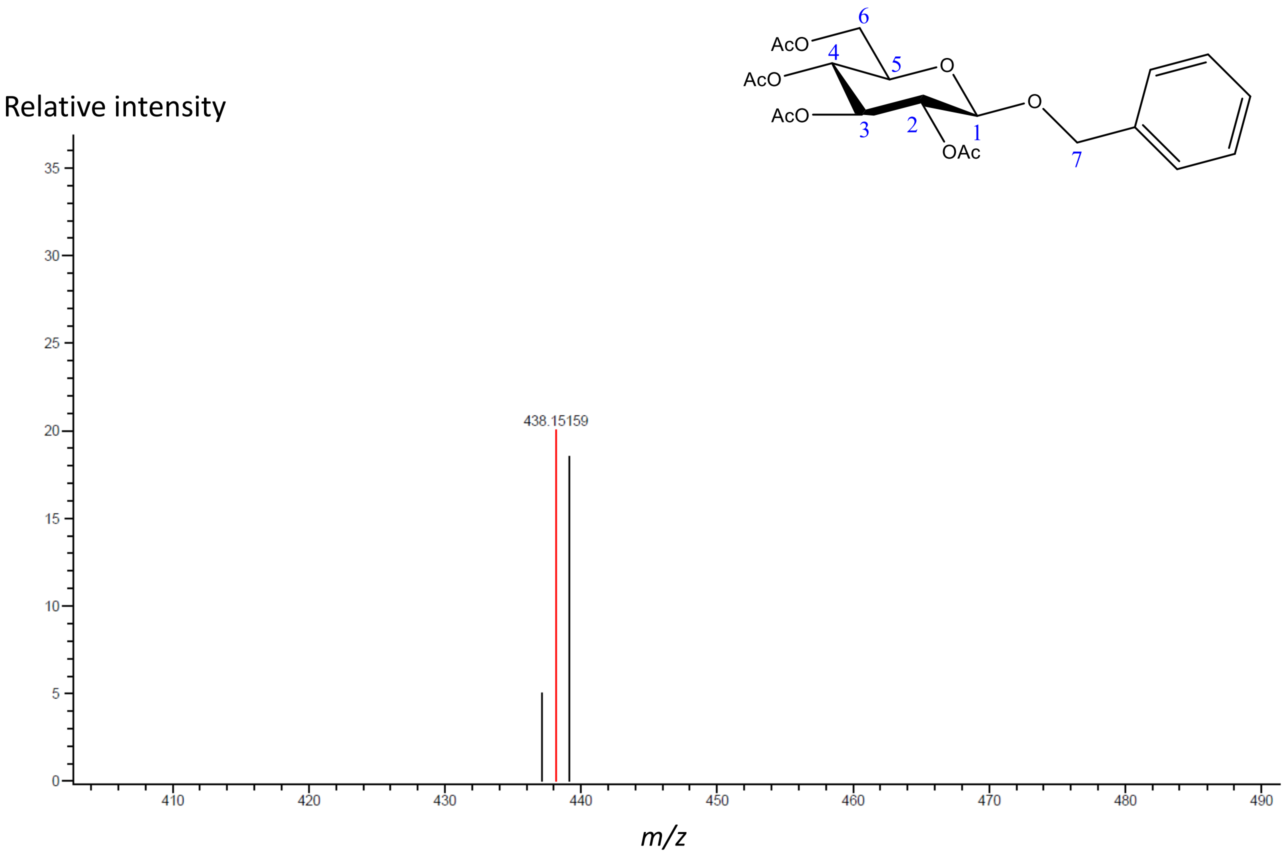

Figure S15: HRFD-MS spectrum of **4**.

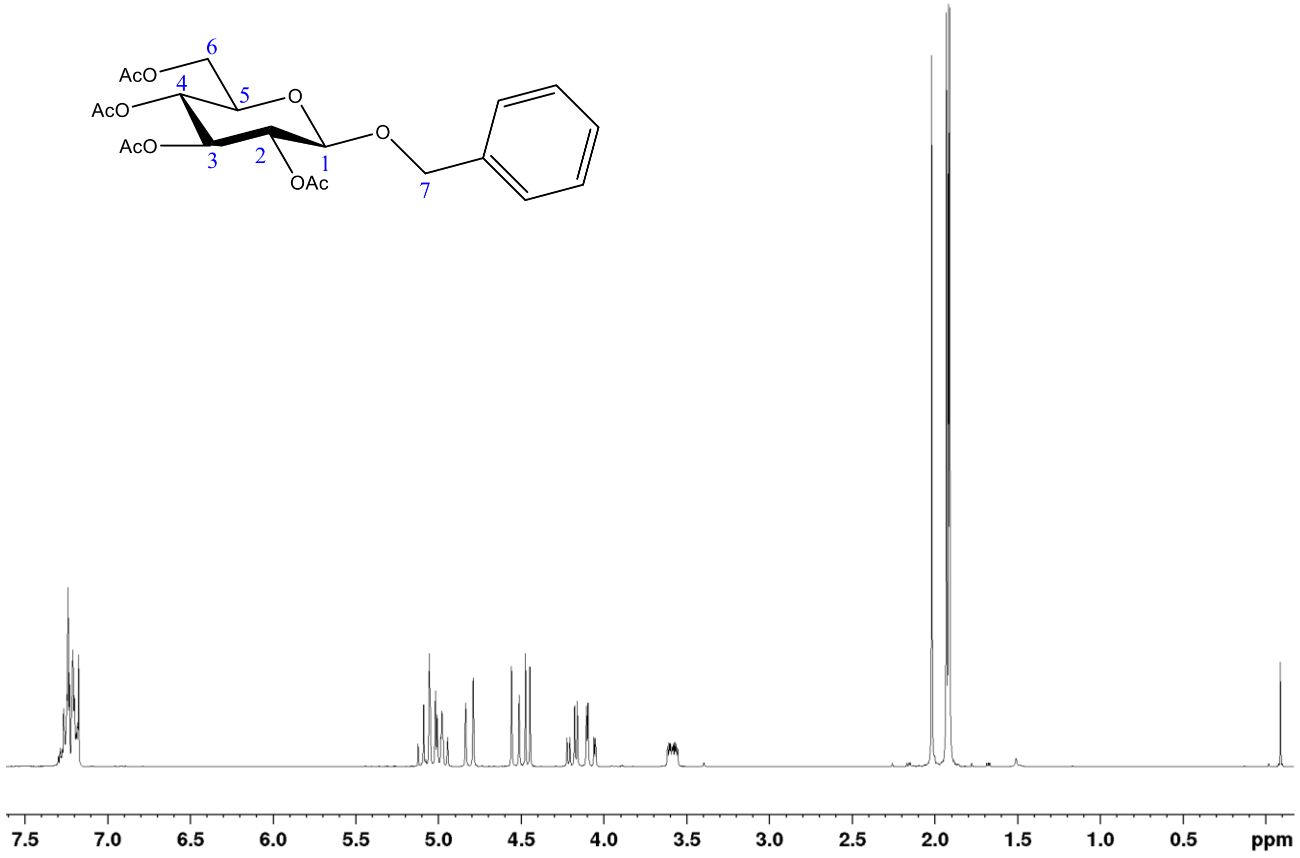

Figure S16: ^{1}H NMR spectrum of compound **4** (270 MHz, $CDCl_3$).

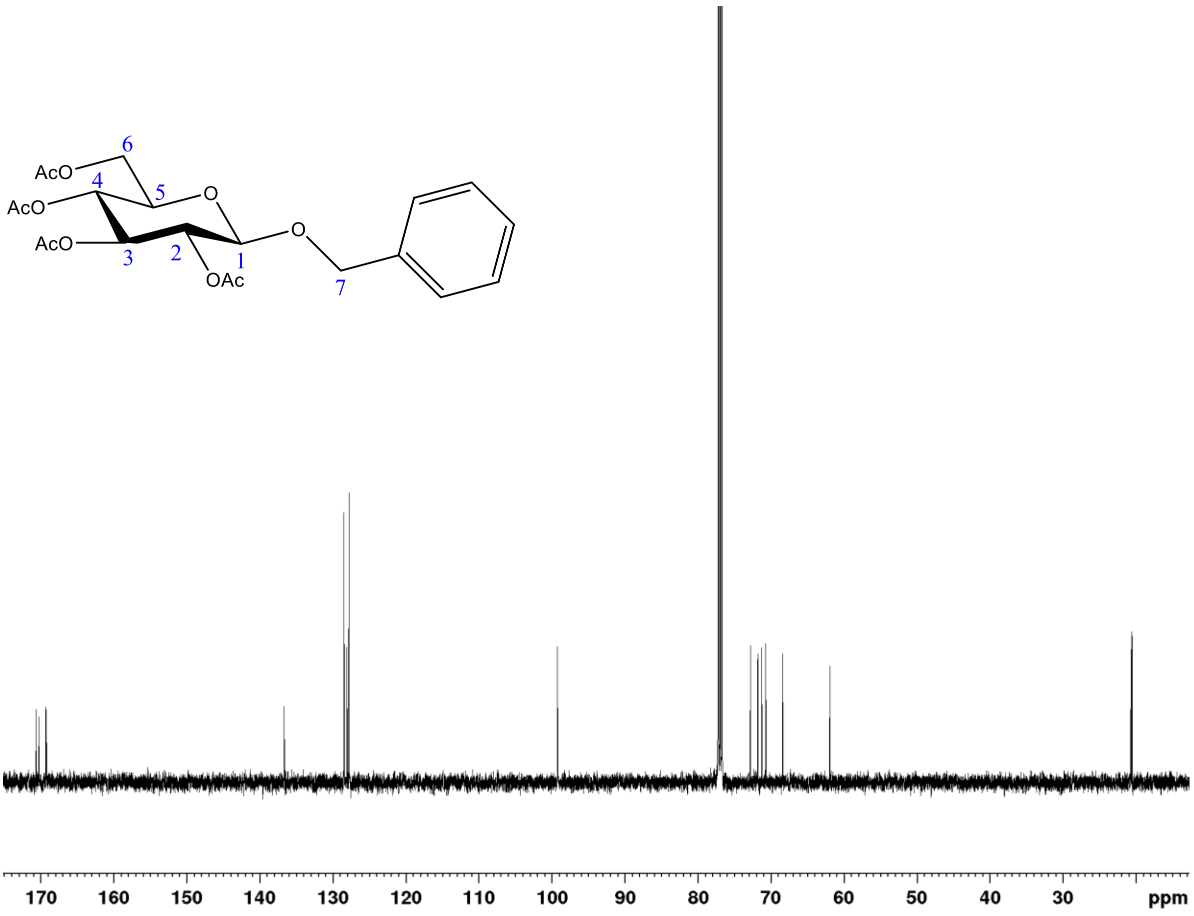

Figure S17: ^{13}C NMR spectrum of compound **4** (126 MHz, $CDCl_3$).

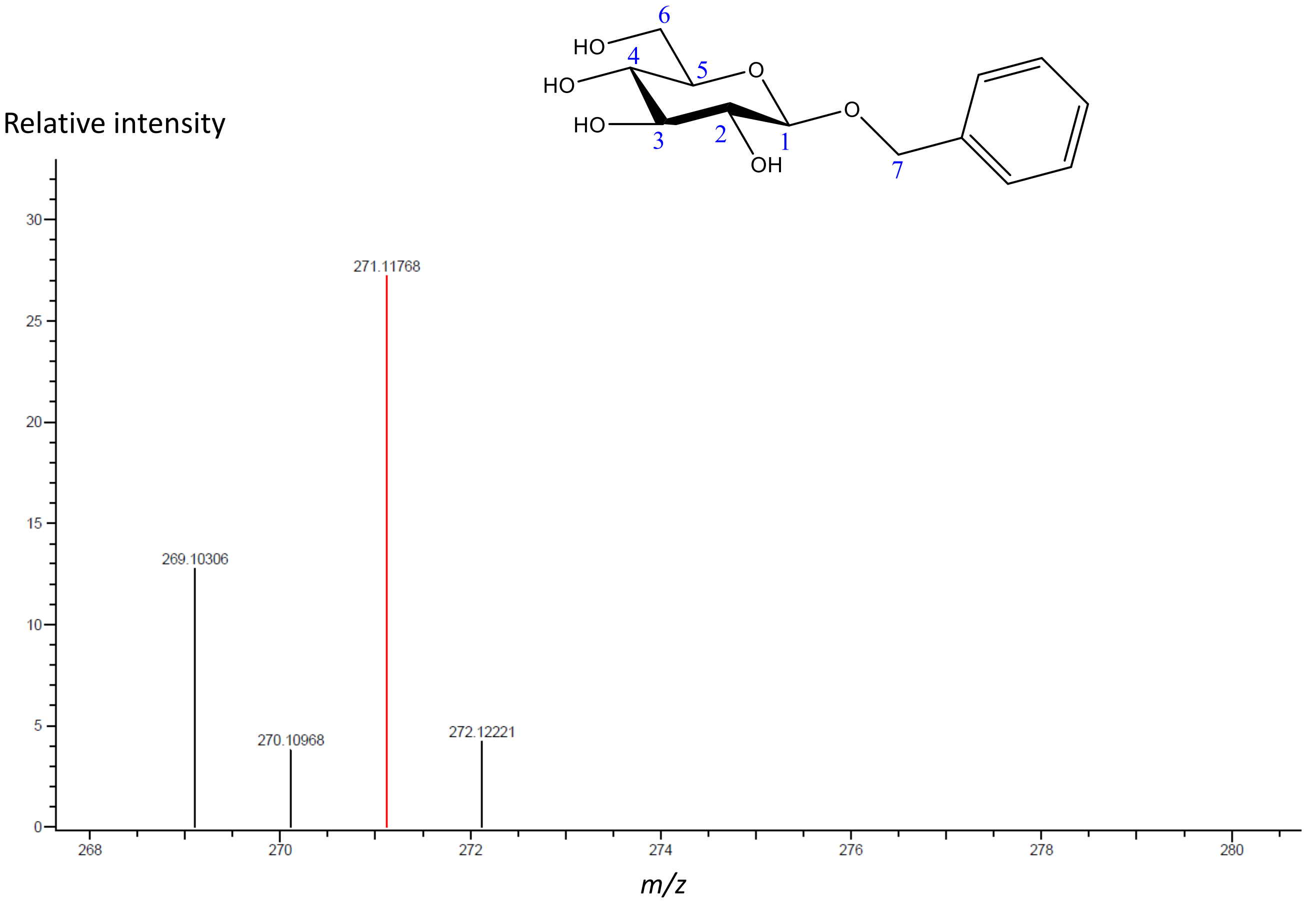

Figure S18: HRFD-MS spectrum of compound **5**.

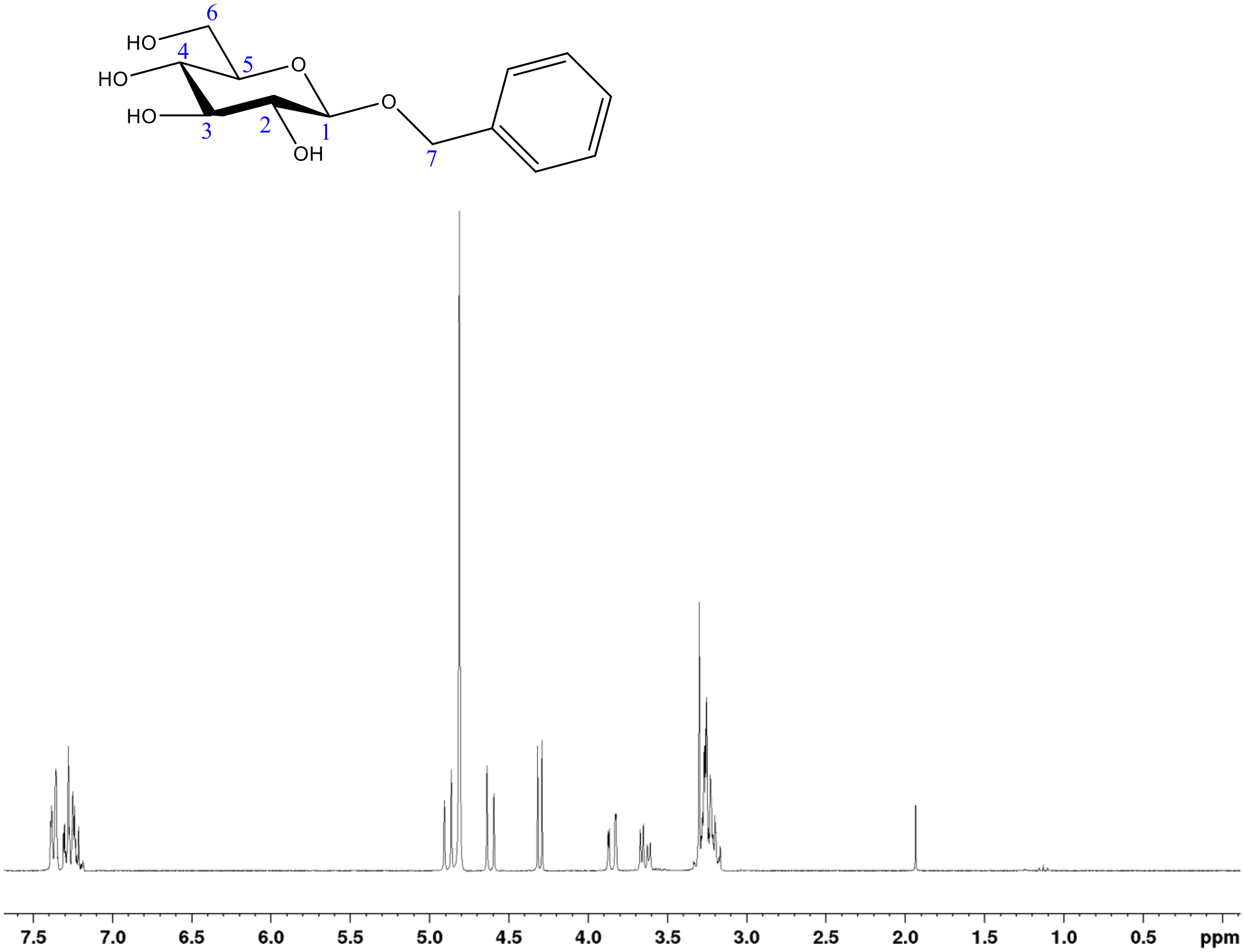

Figure S19: ^{1}H NMR spectrum of compound **5** (270 MHz, CD_3OD).

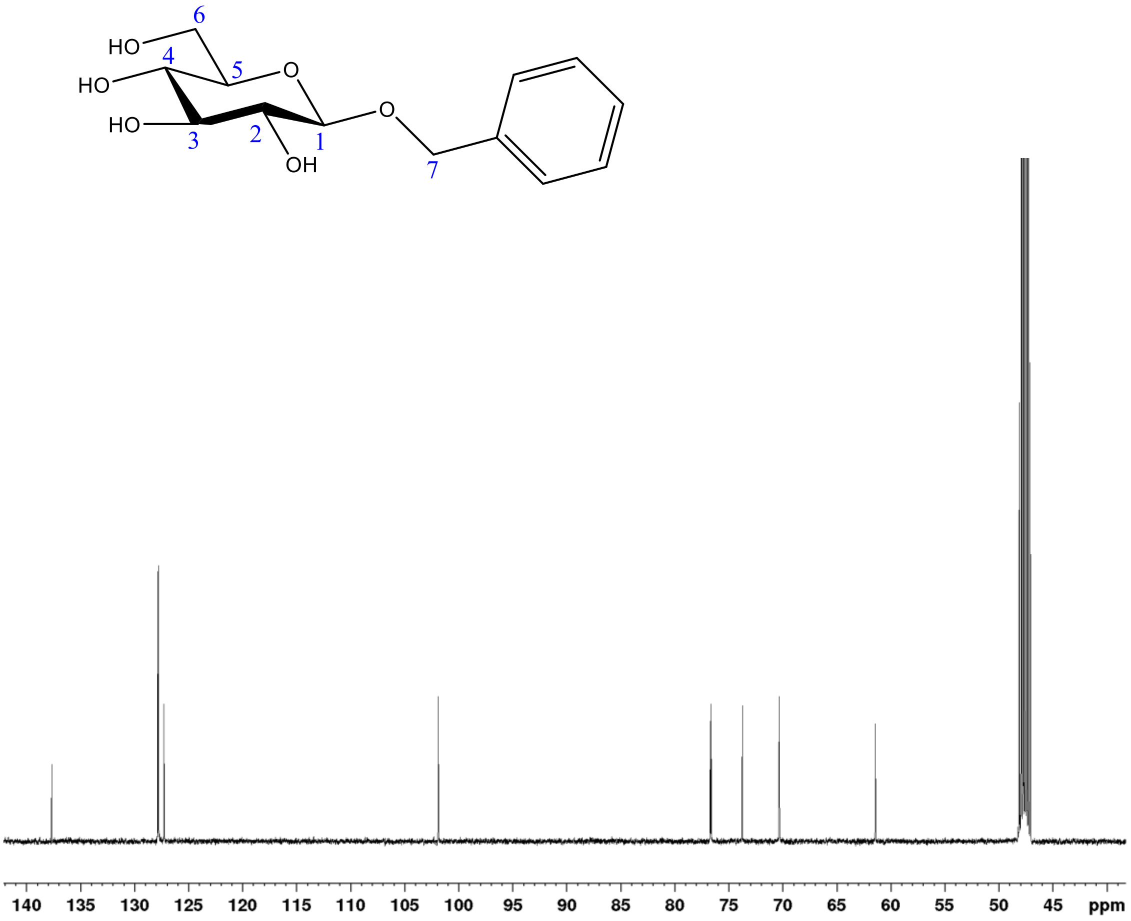

Figure S20: ^{13}C NMR spectrum of compound **5** (126 MHz, CD_3OD).

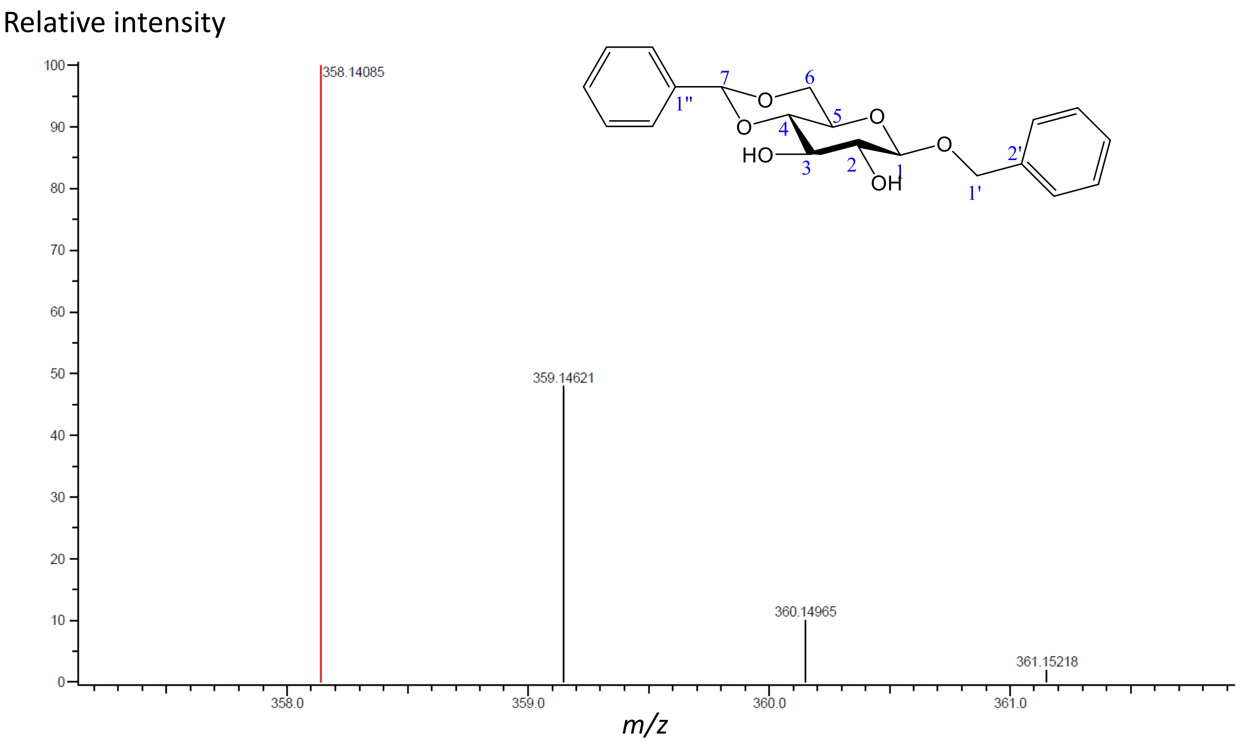

Figure S21: HRFD-MS spectrum of compound **6**.

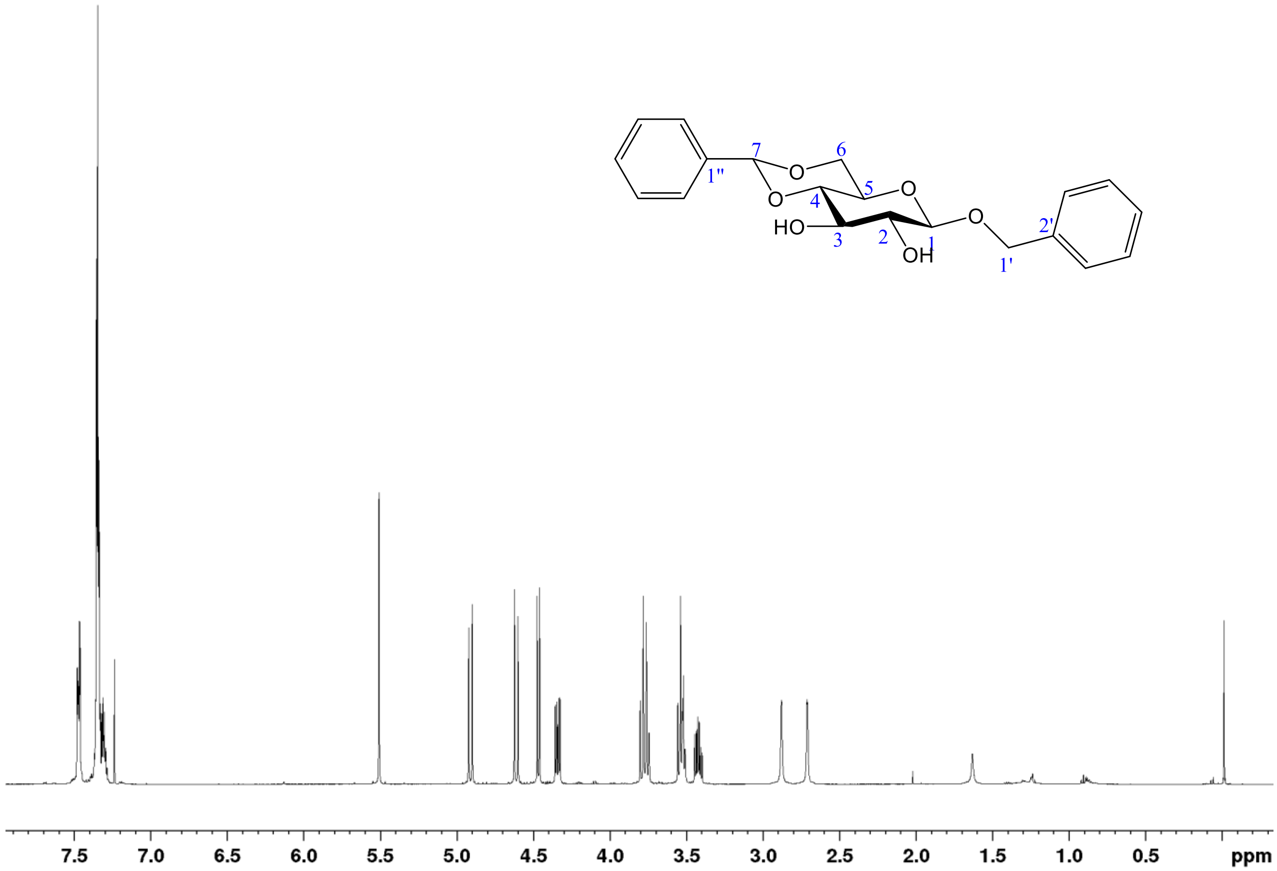

Figure S22: ^{1}H NMR spectrum of compound **6** (500 MHz, $CDCl_3$).

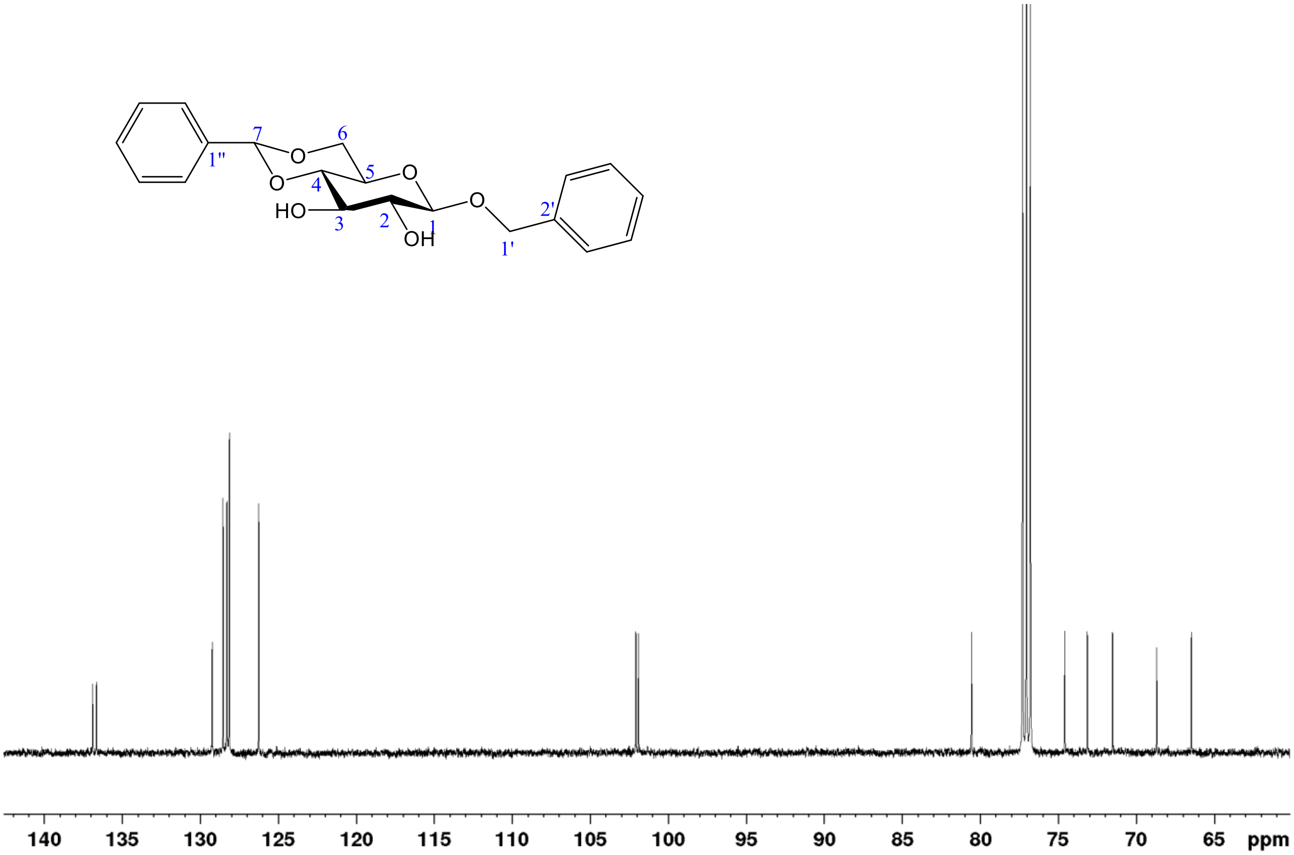

Figure S23: ^{13}C NMR spectrum of compound **6** (126 MHz, $CDCl_3$).

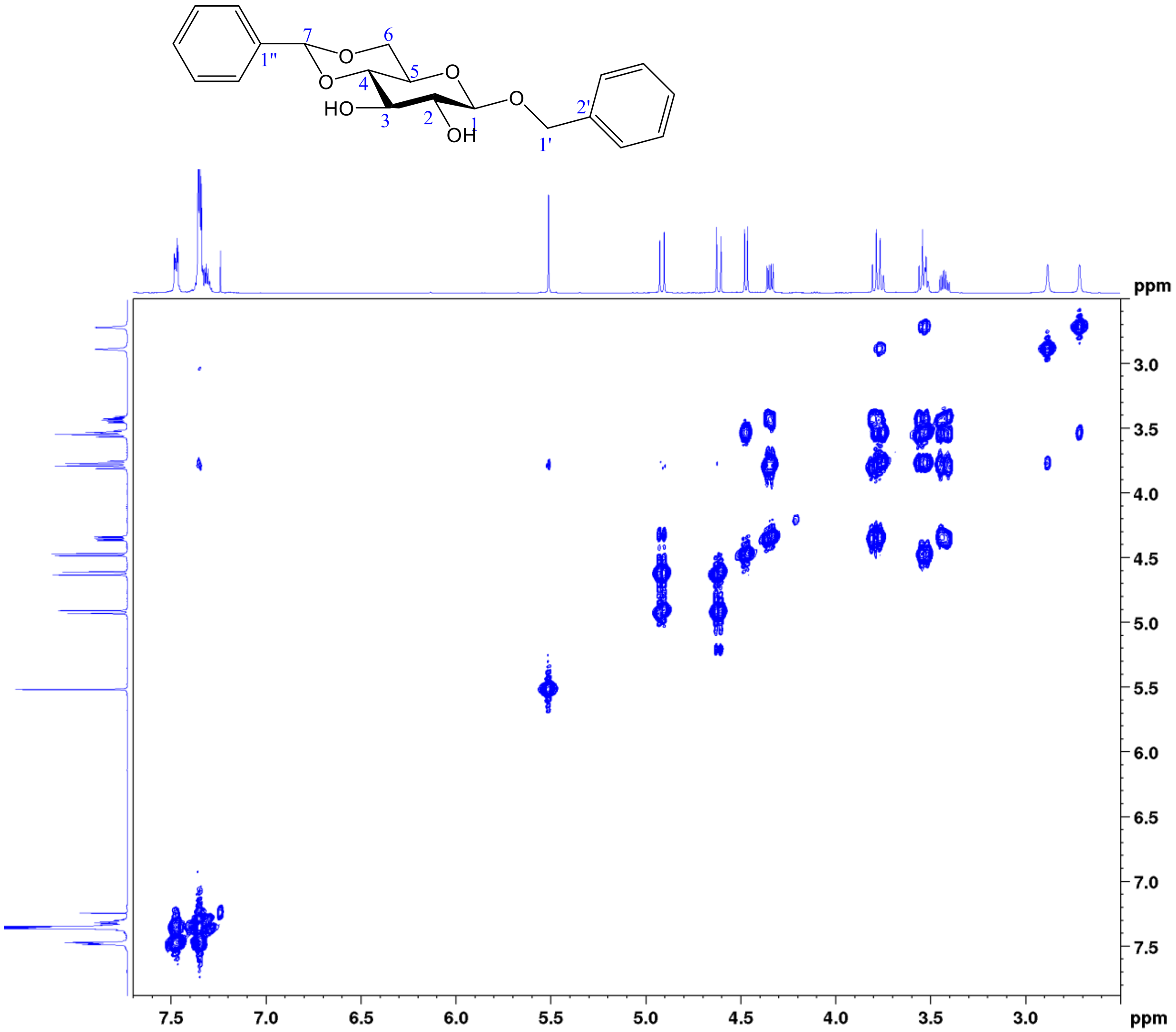

Figure S24: COSY spectrum of compound **6** (500 MHz, $CDCl_3$).

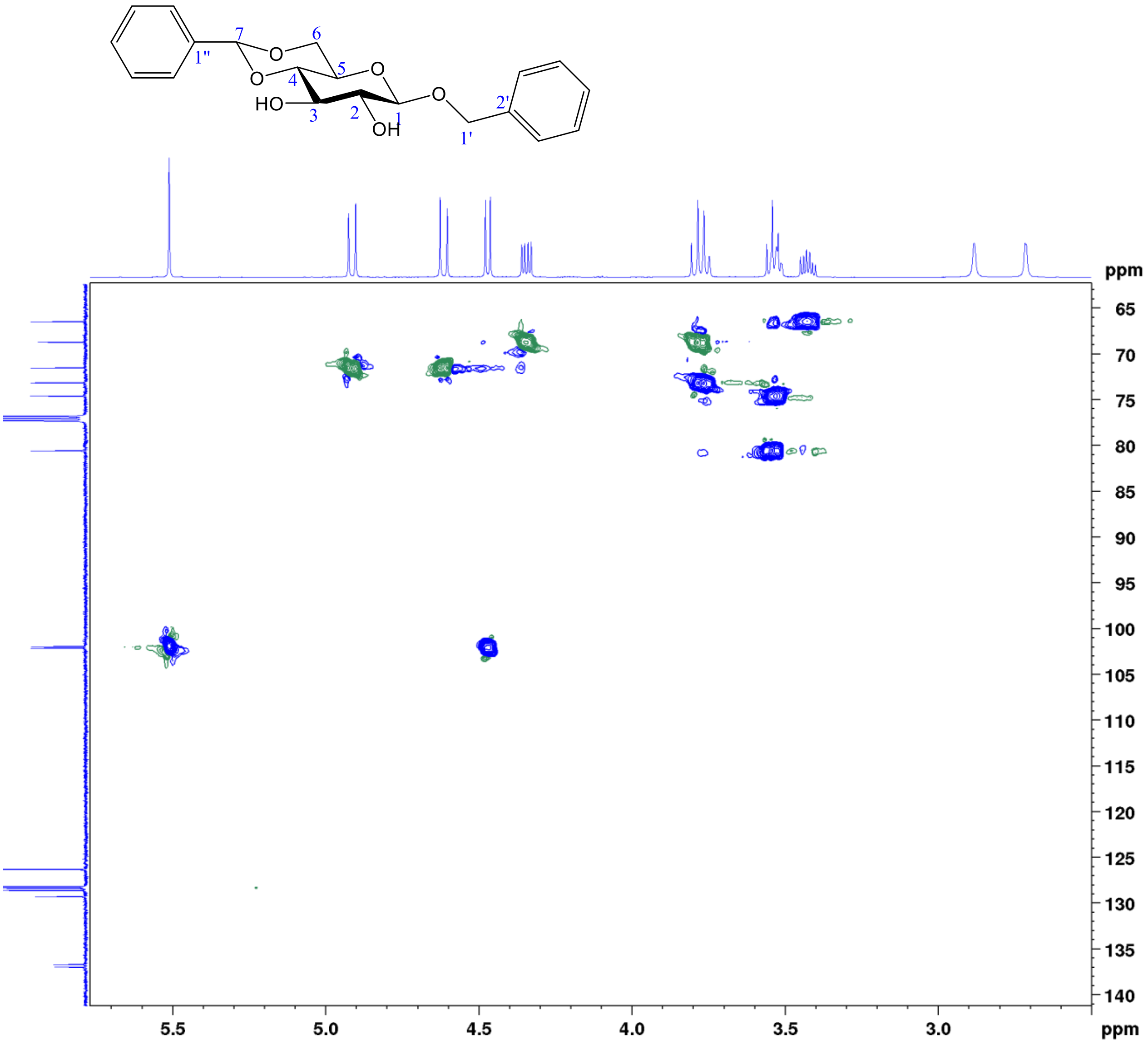

Figure S25: HSQC spectrum of compound **6** (500 MHz, $CDCl_3$).

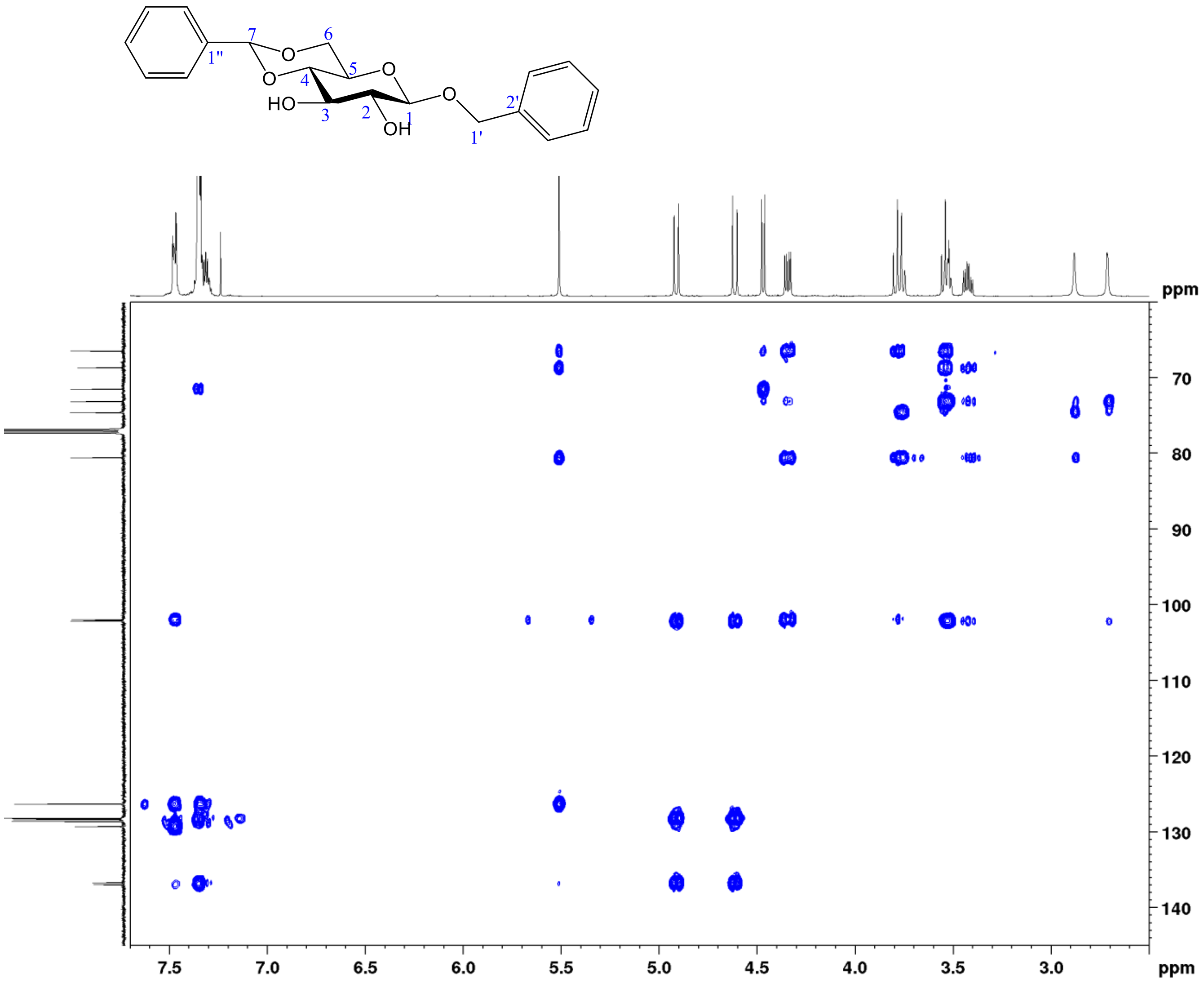

Figure S26: HMBC spectrum of compound **6** (500 MHz, $CDCl_3$).

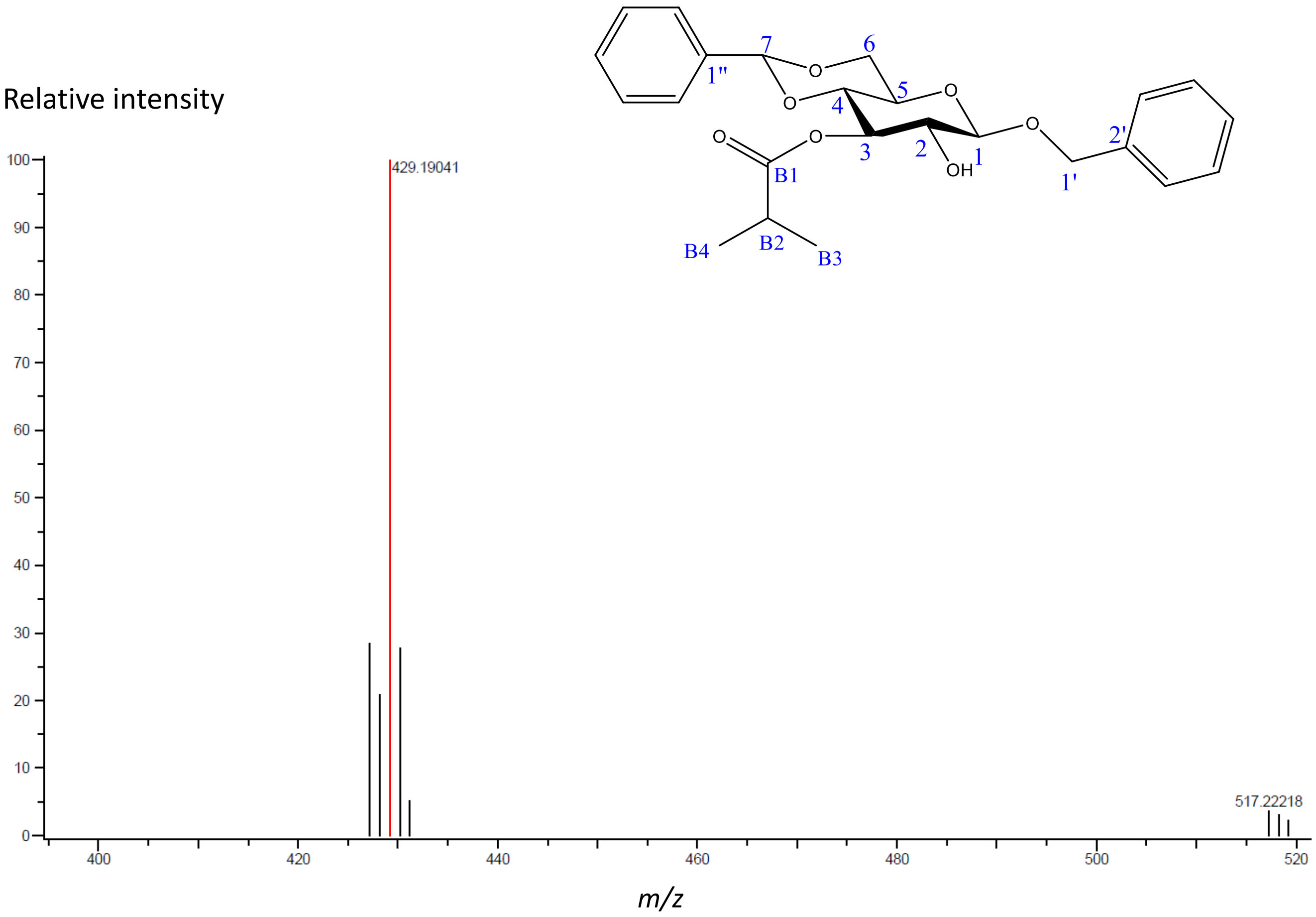

Figure S27: HRFD-MS spectrum of compound **7**.

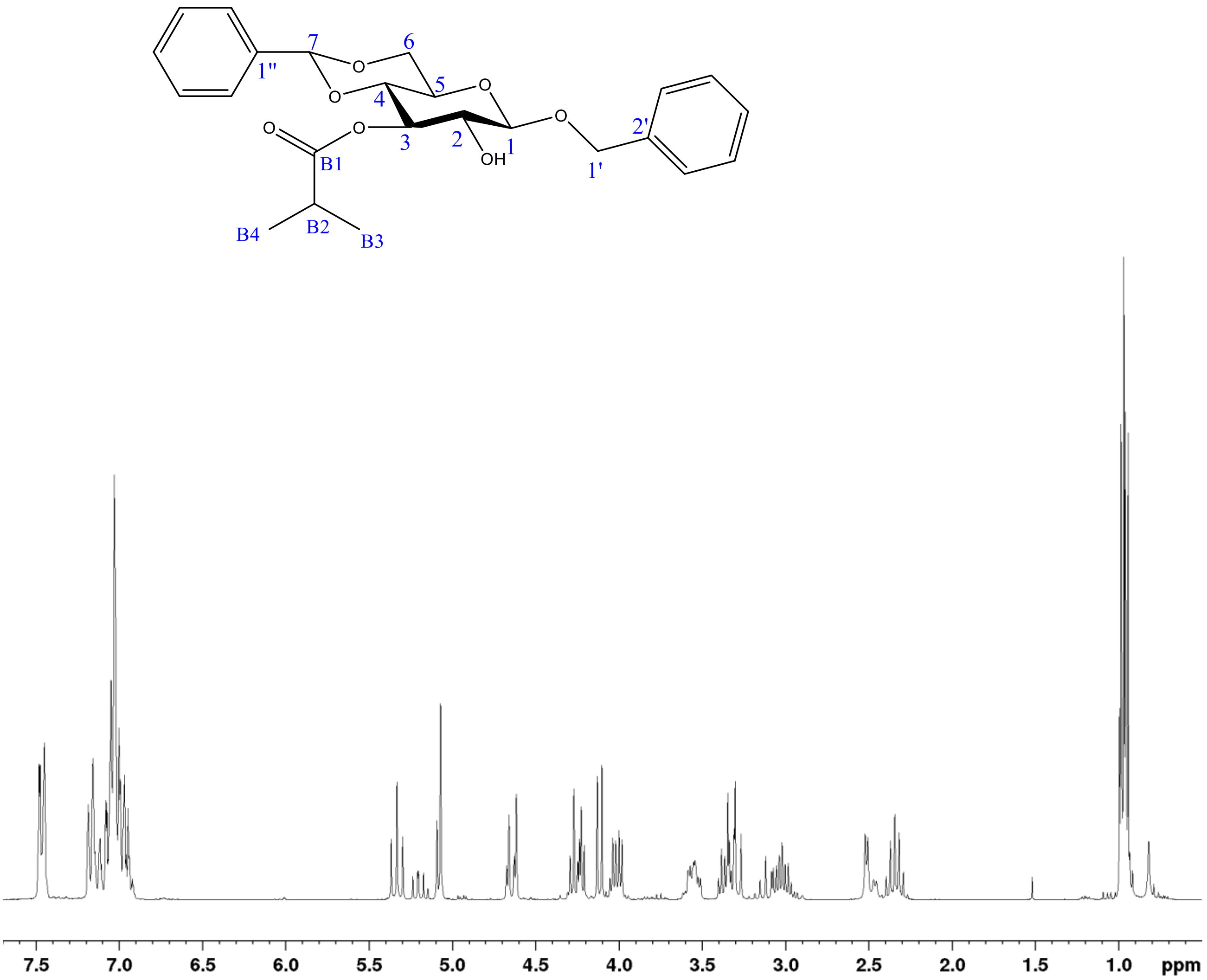

Figure S28: ^{1}H NMR spectrum of compound **7** (270 MHz, C_6D_6).

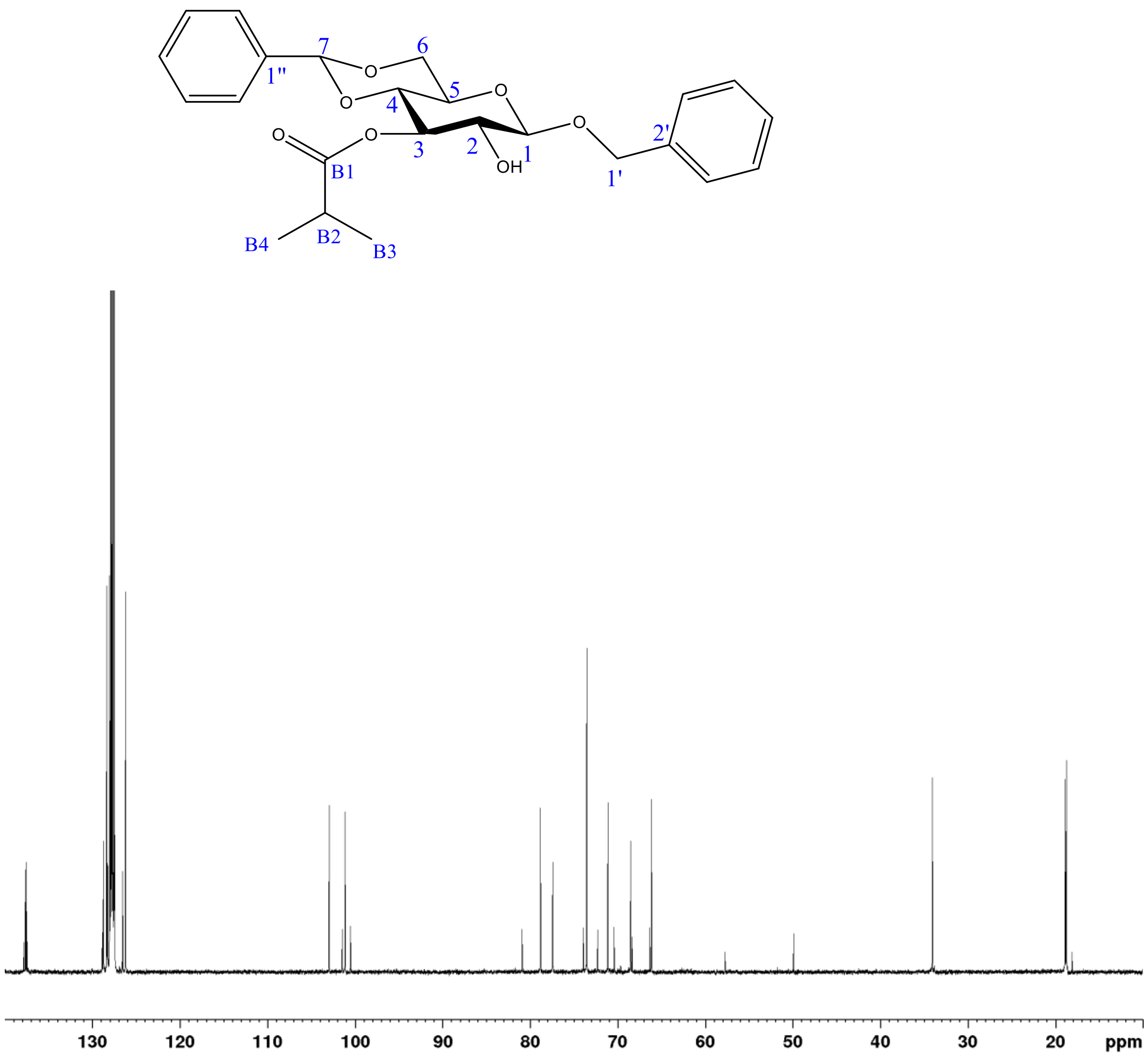

Figure S29: ^{13}C NMR spectrum of compound **7** (126 MHz, C_6D_6).

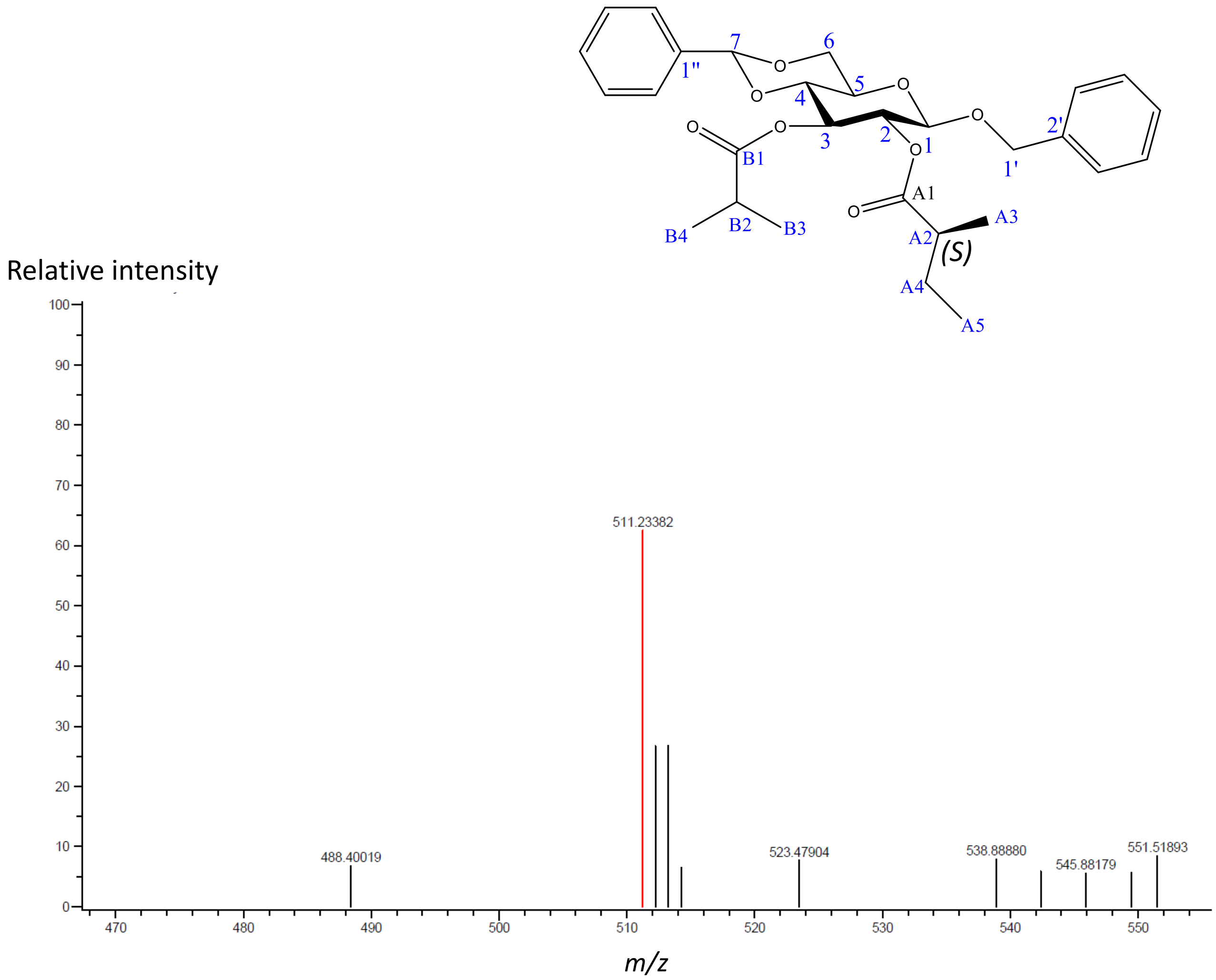

Figure S30: HRFD-MS spectrum of compound **8**.

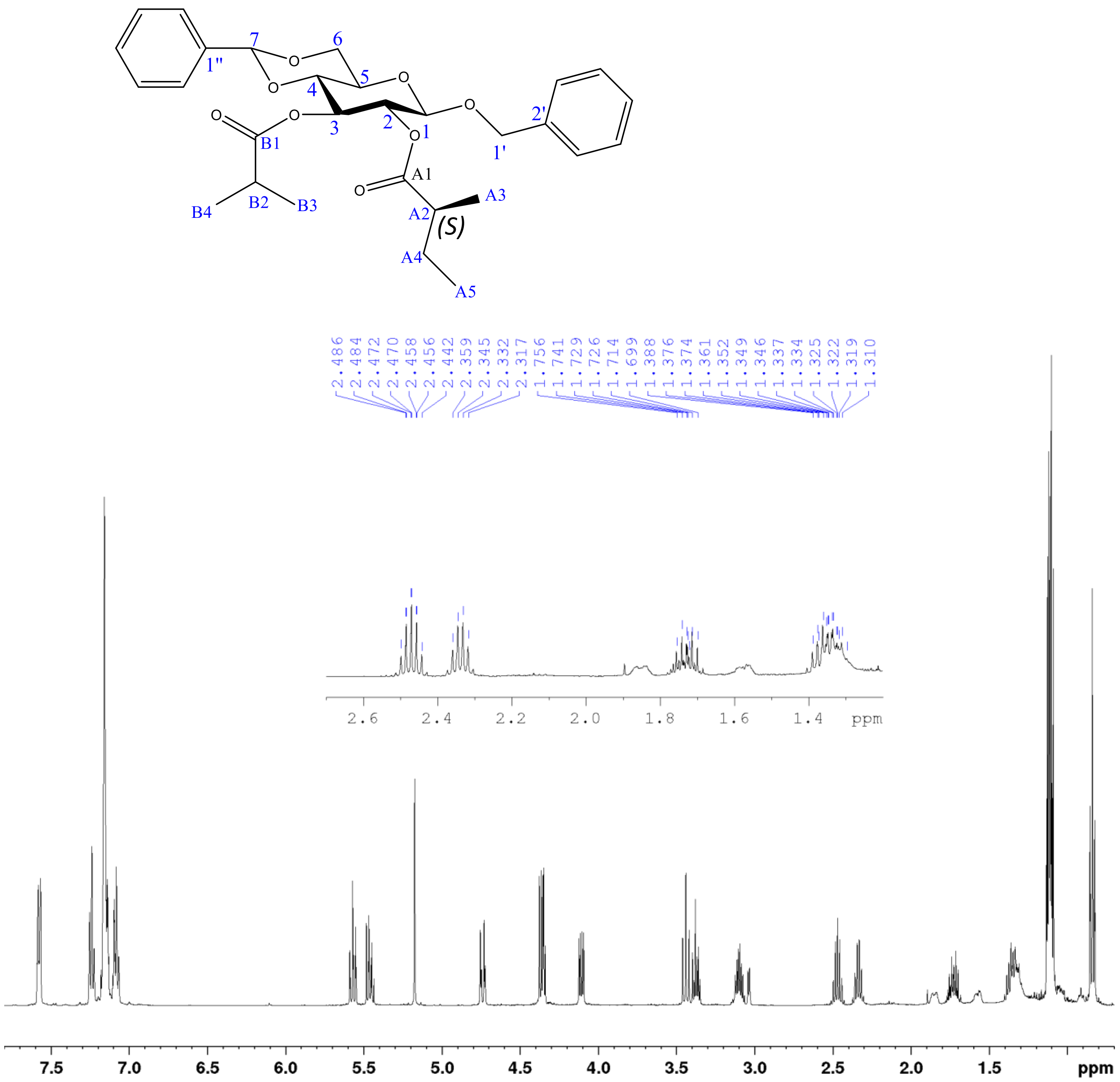

Figure S31: ^{1}H NMR spectrum of compound **8** (500 MHz, C_6D_6).

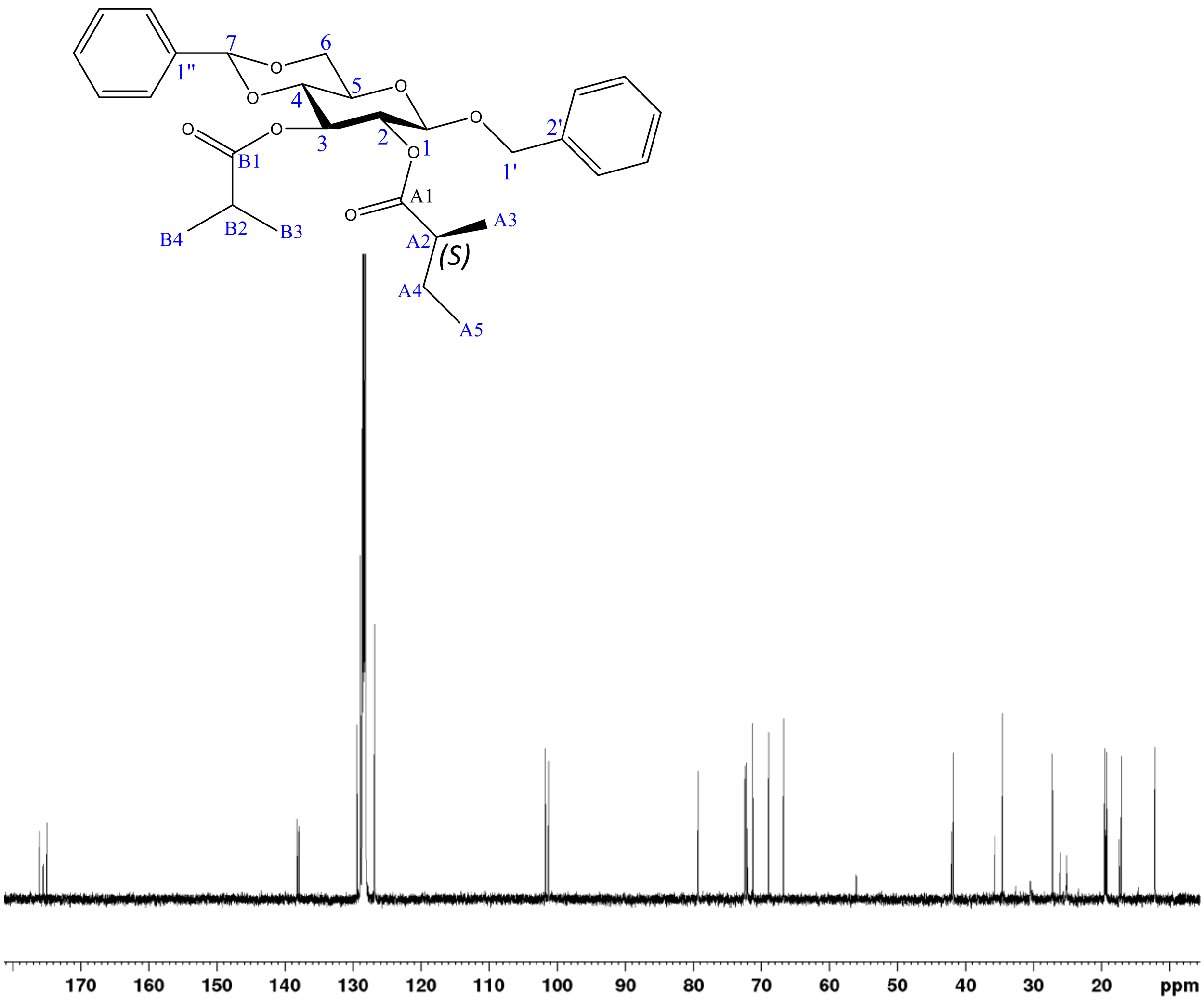

Figure S32: ^{13}C NMR spectrum of compound **8** (126 MHz, C_6D_6).

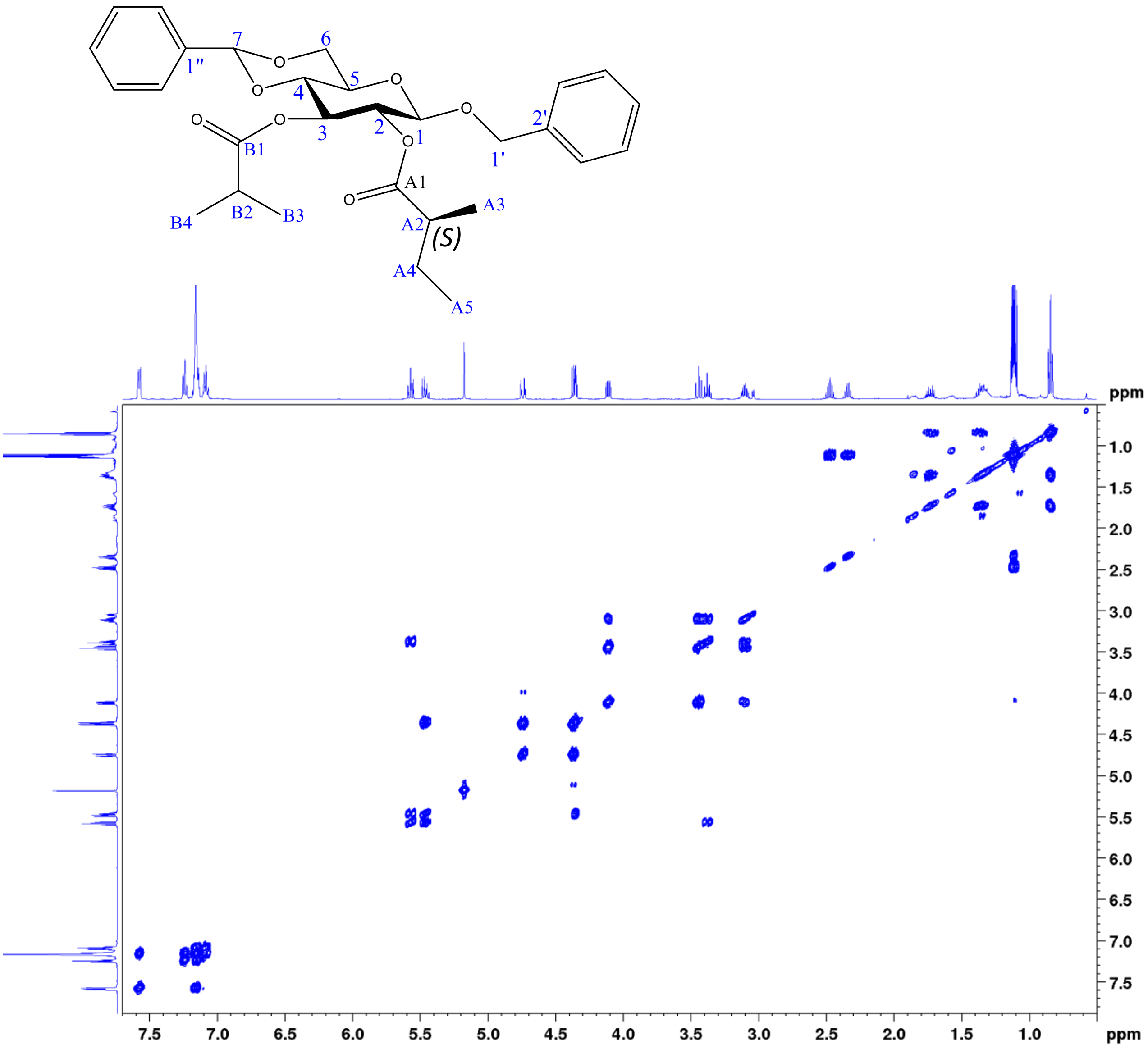

Figure S33: COSY spectrum of compound **8** (500 MHz, C_6D_6).

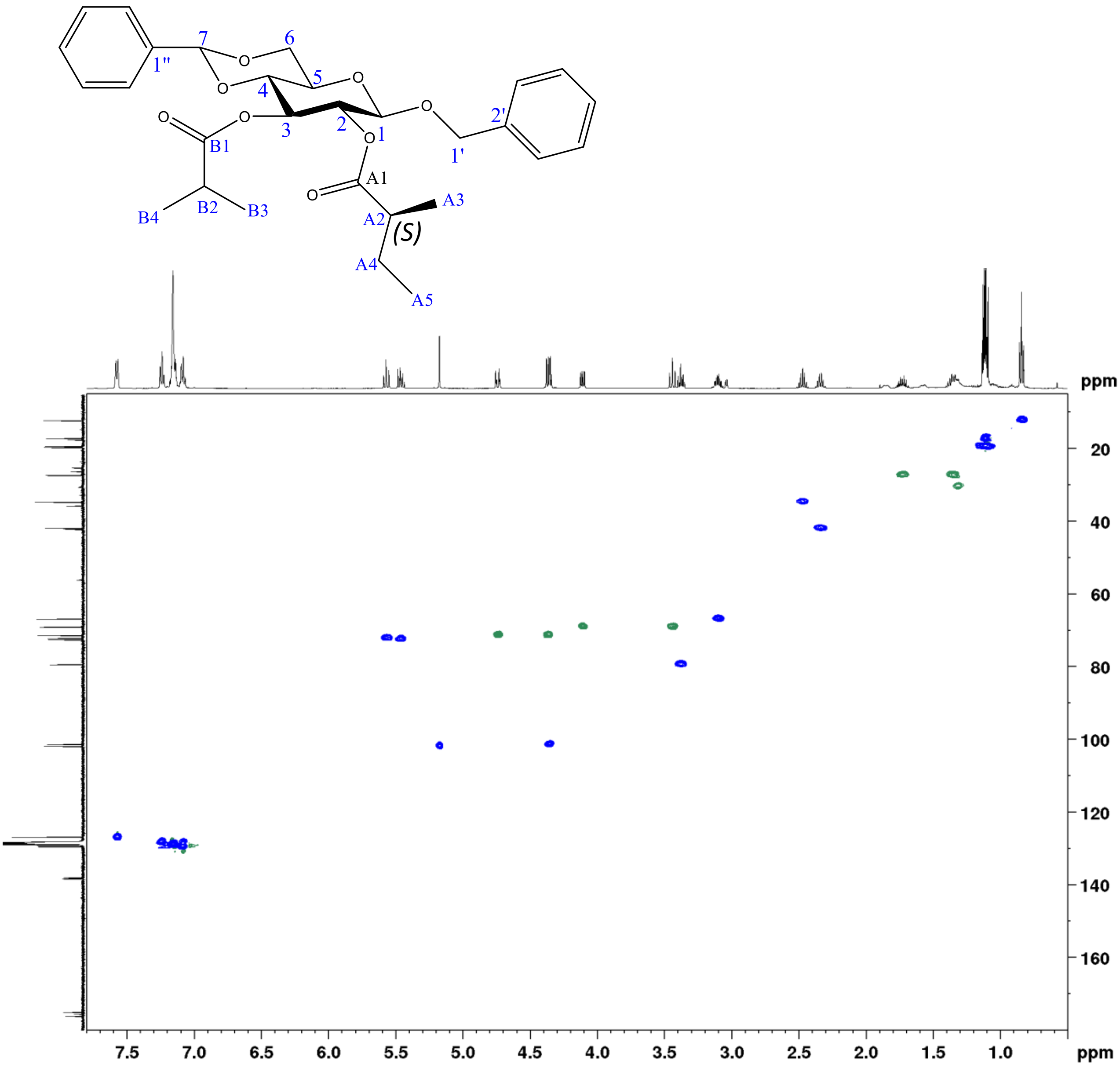

Figure S34: HSQC spectrum of compound **8** (500 MHz, C_6D_6).

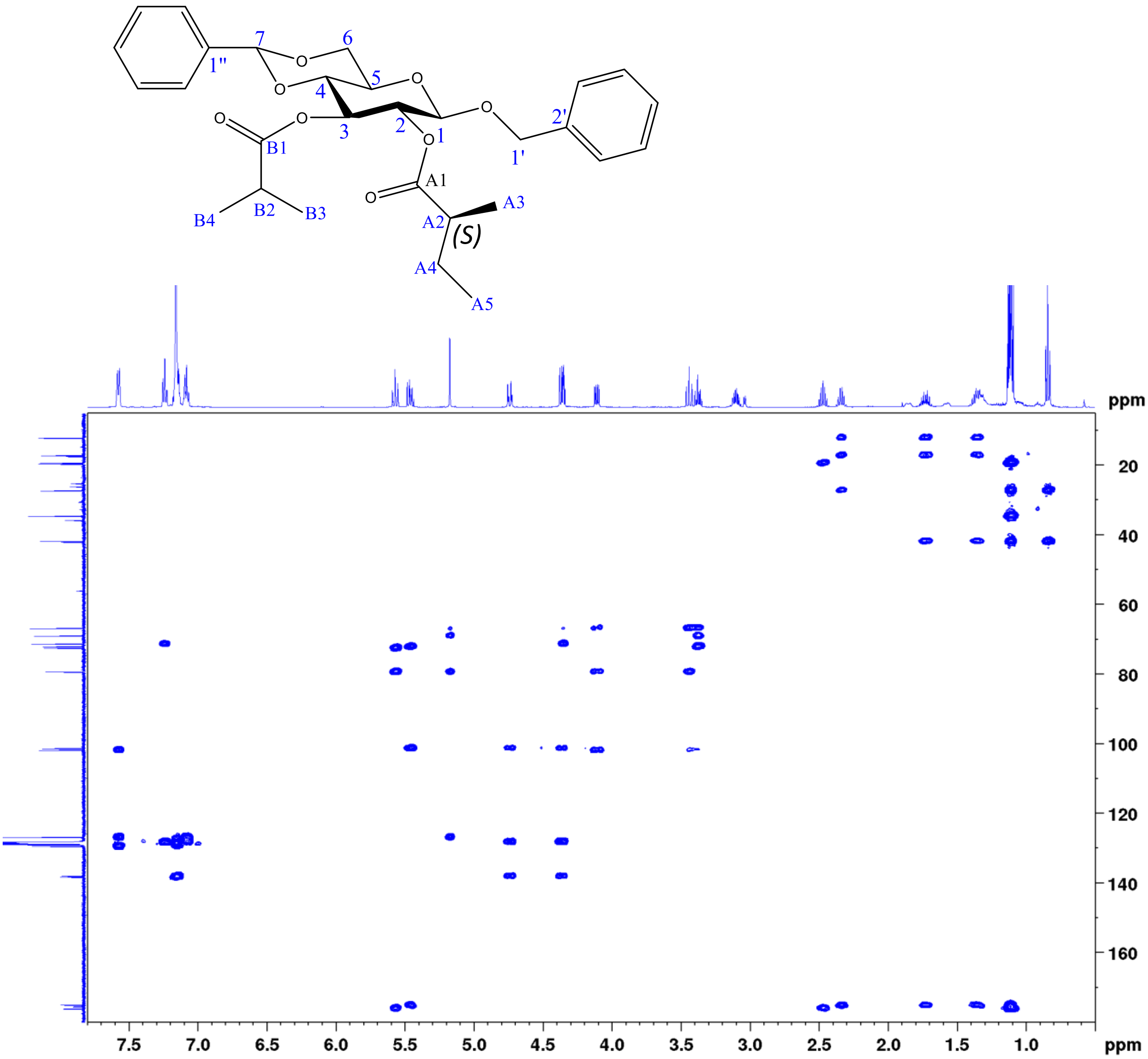

Figure S35: HMBC spectrum of compound **8** (500 MHz, C_6D_6).

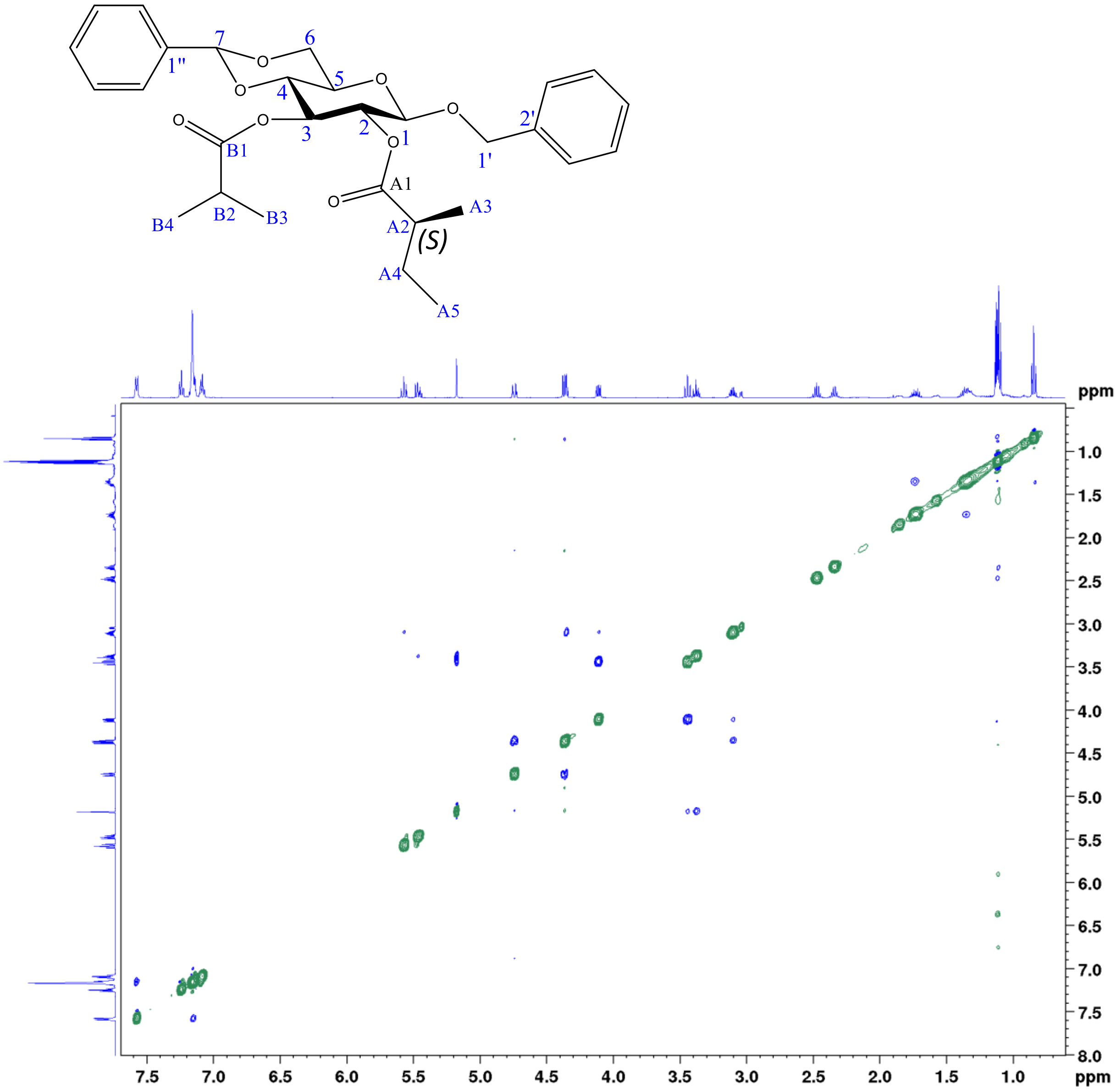

Figure S36: NOESY spectrum of compound **8** (500 MHz, C_6D_6).

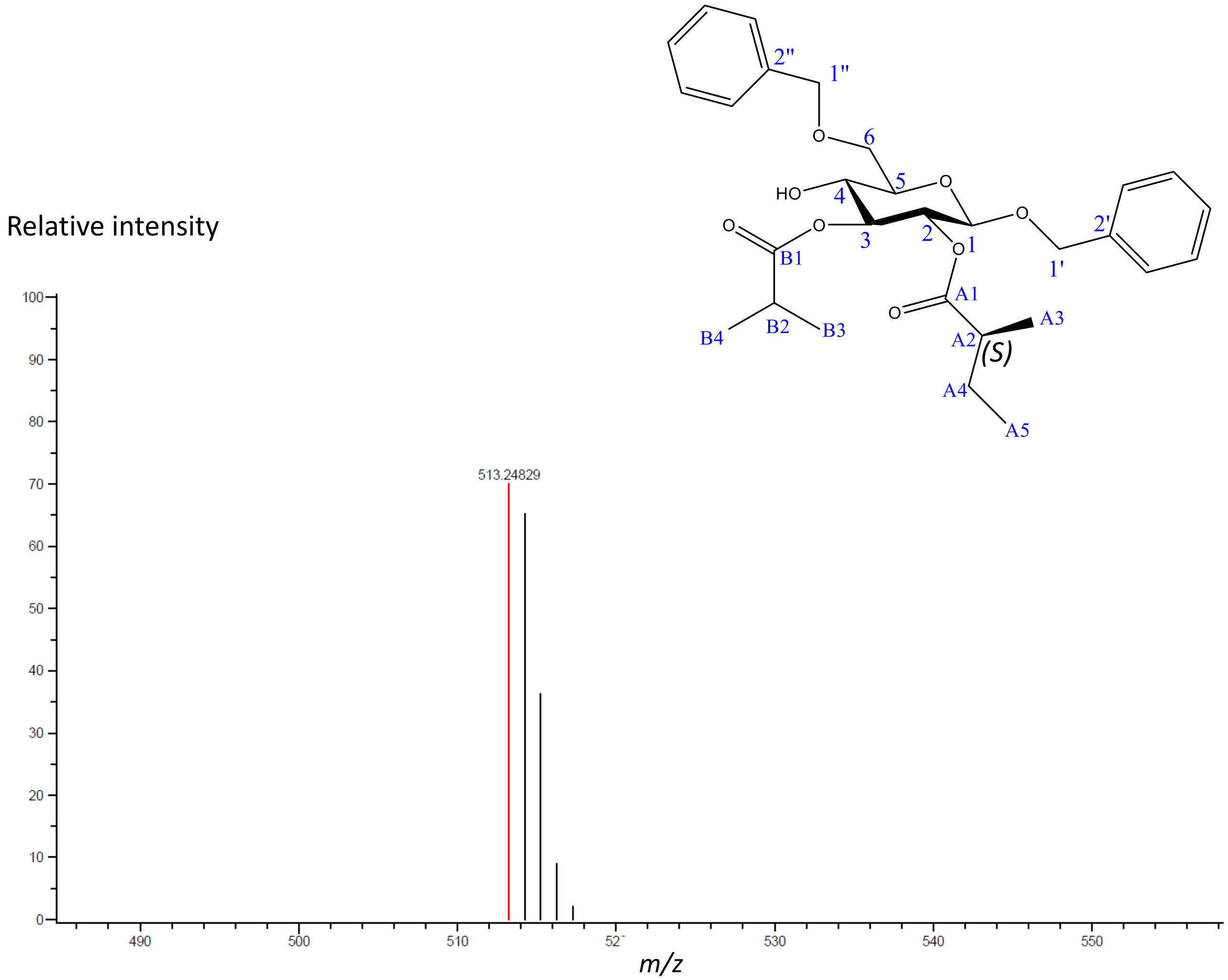

Figure S37: HRFD-MS spectrum of compound **9**.

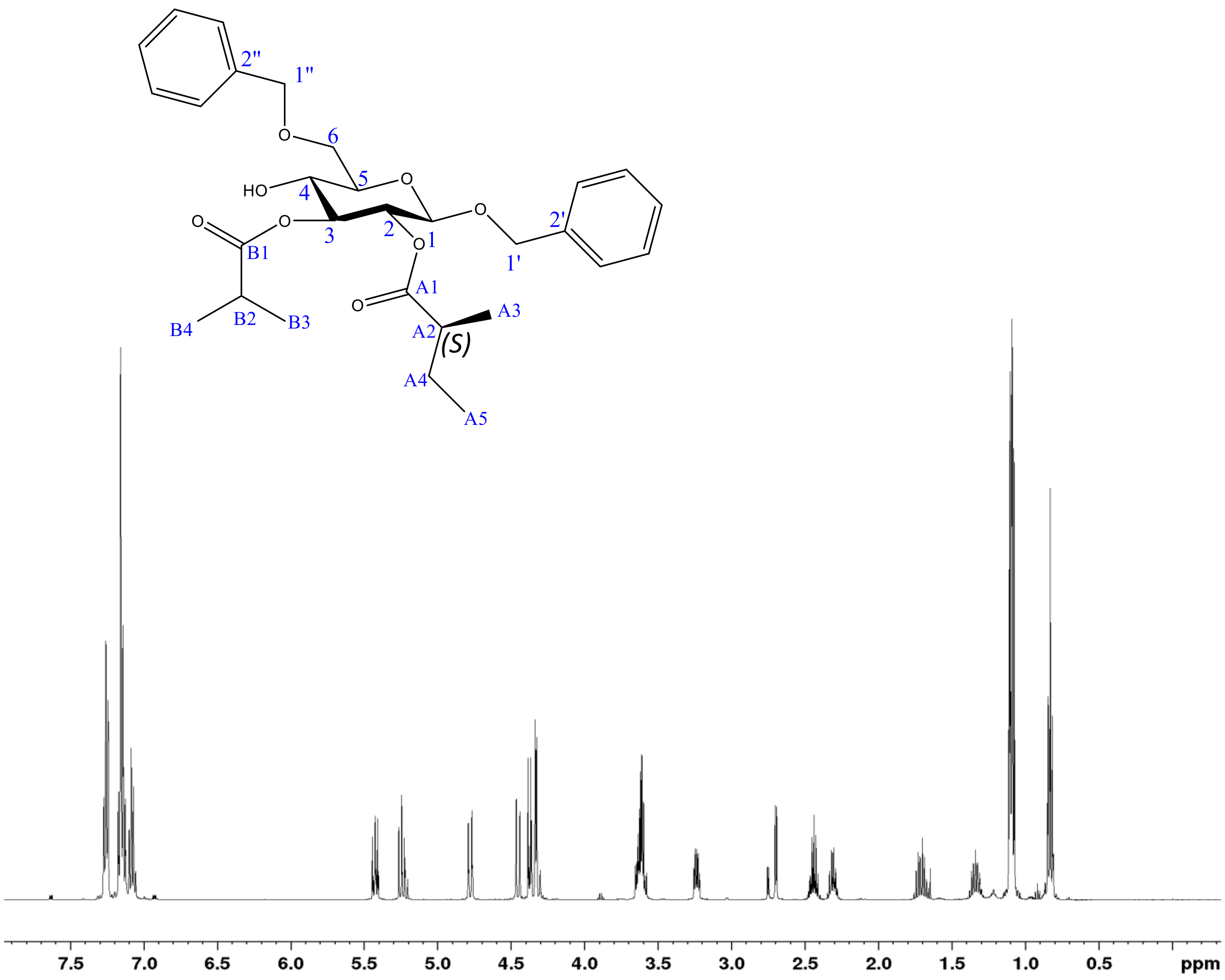

Figure S38: ^{1}H NMR spectrum of compound **9** (500 MHz, C_6D_6).

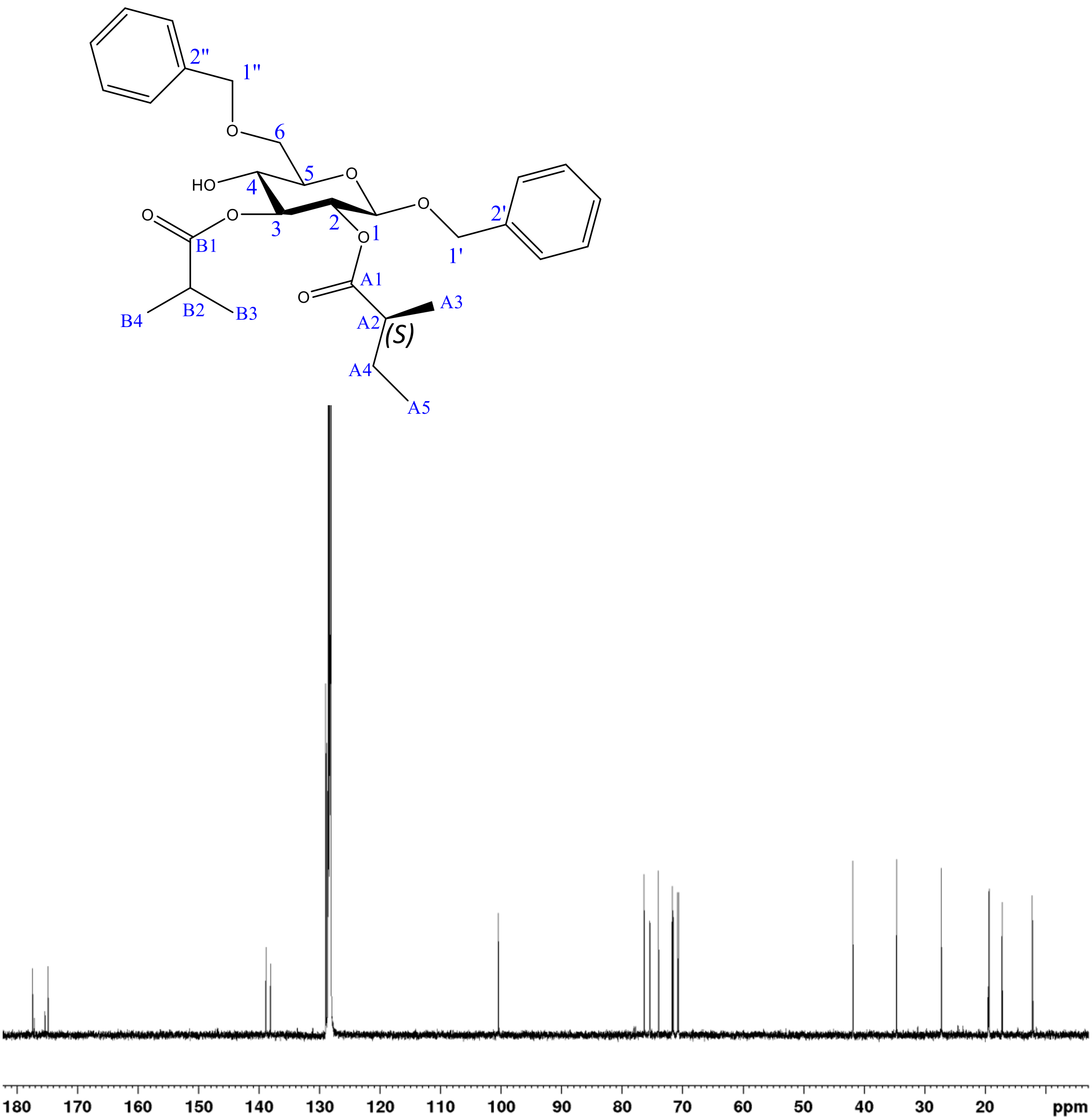

Figure S39: ^{13}C NMR spectrum of compound **9** (126 MHz, C_6D_6).

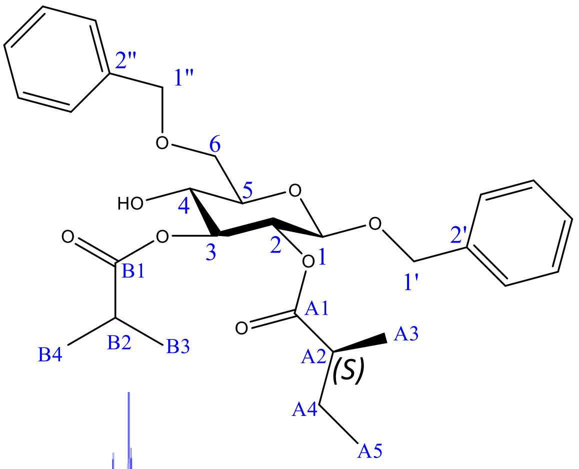

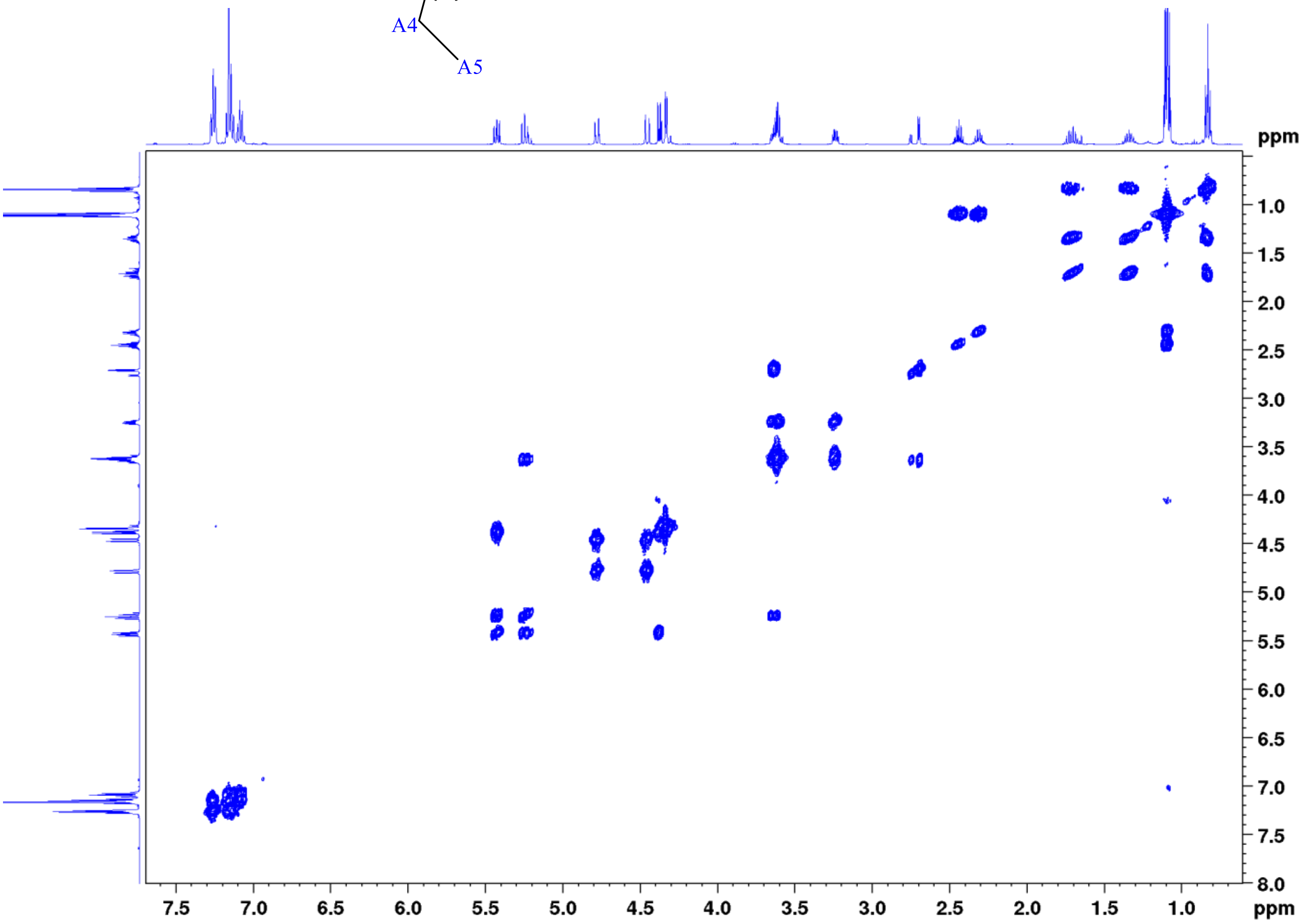

Figure S40: COSY spectrum of compound **9** (500 MHz, C_6D_6).

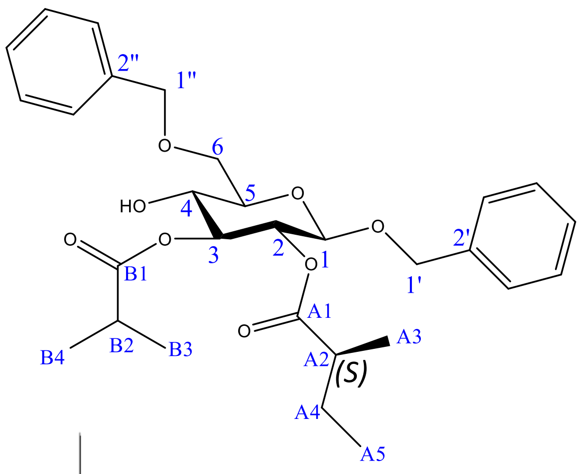

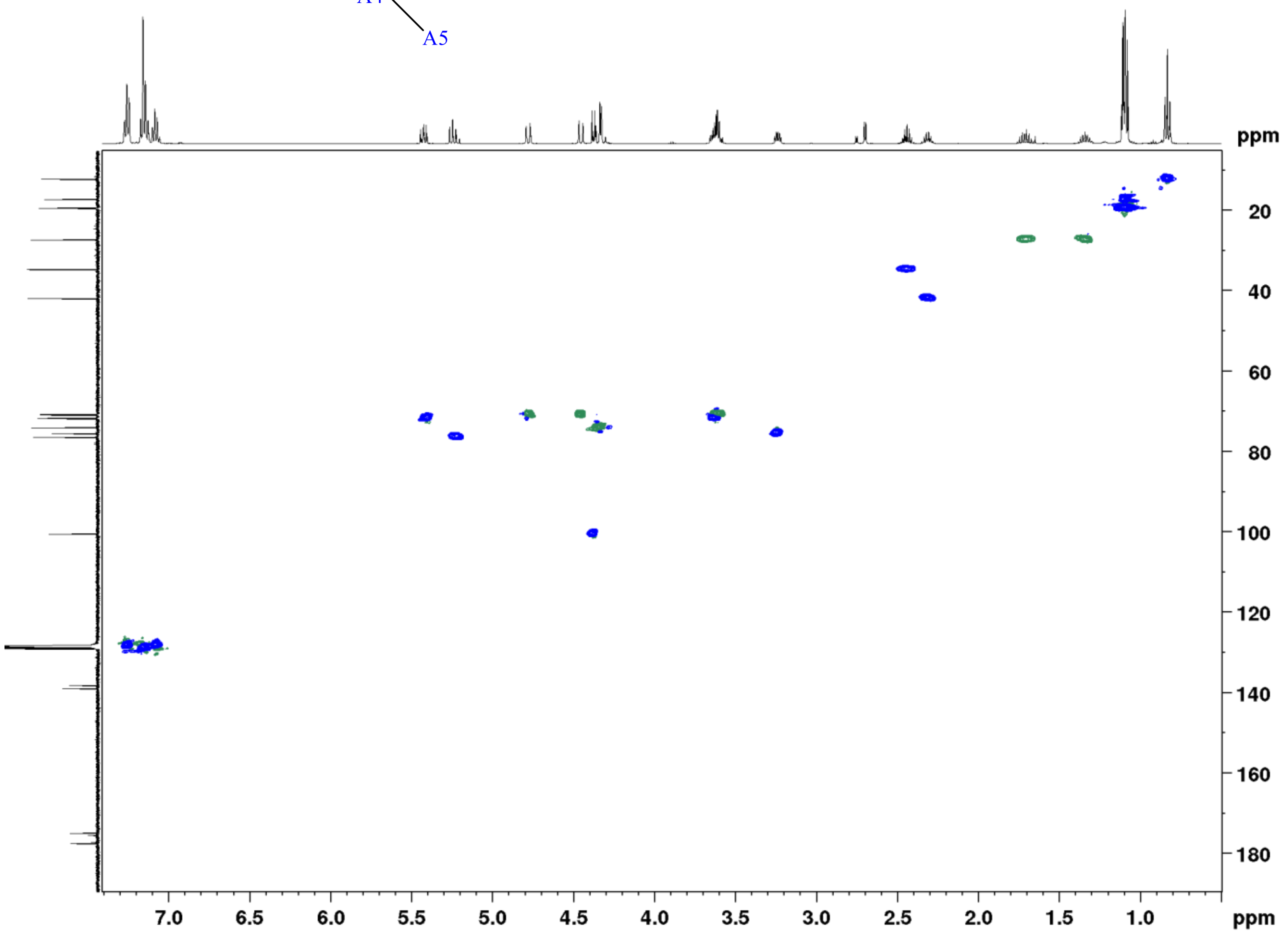

Figure S41: HSQC spectrum of compound **9** (500 MHz, C_6D_6).

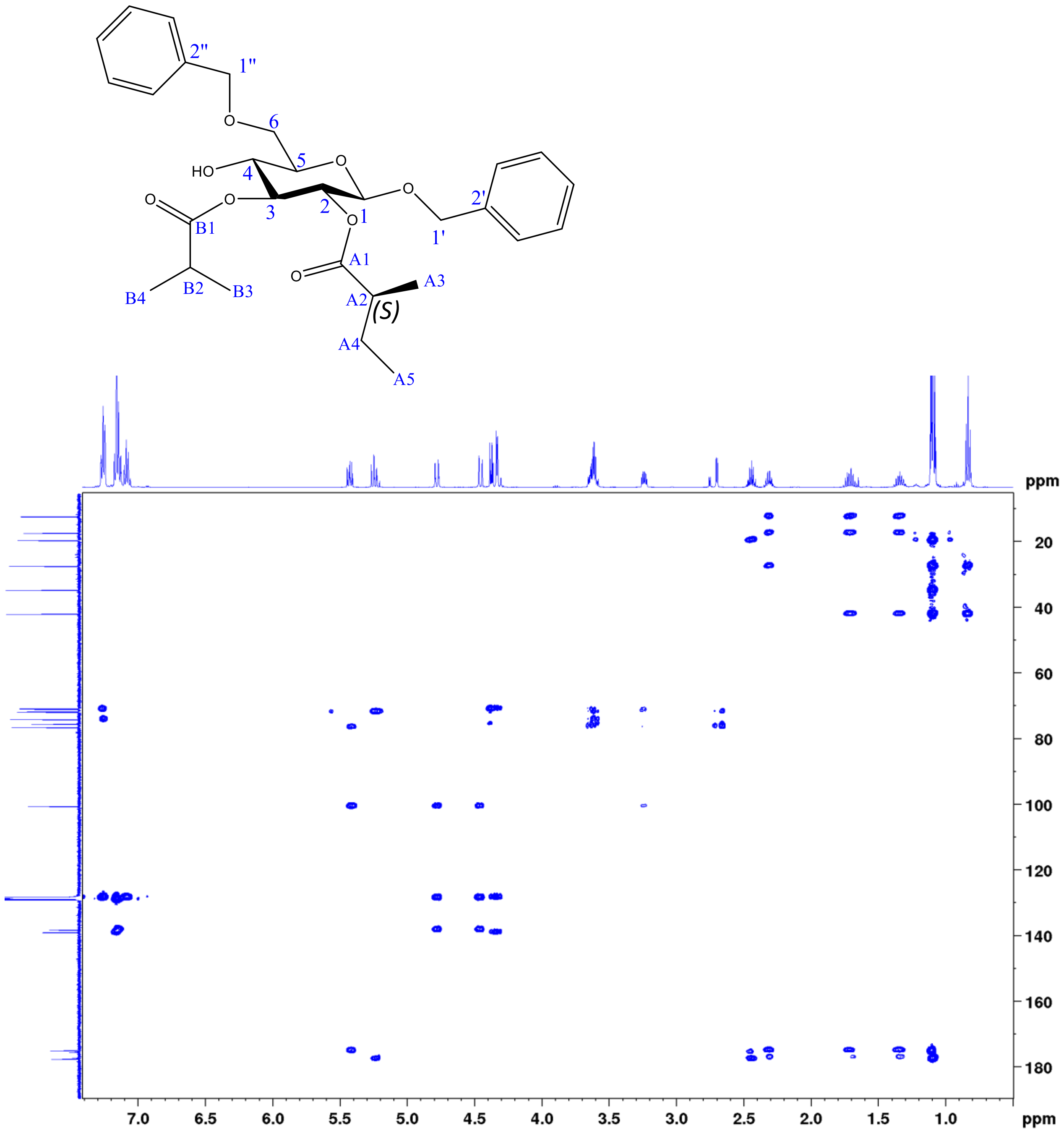

Figure S42: HMBC spectrum of compound **9** (500 MHz, C_6D_6).

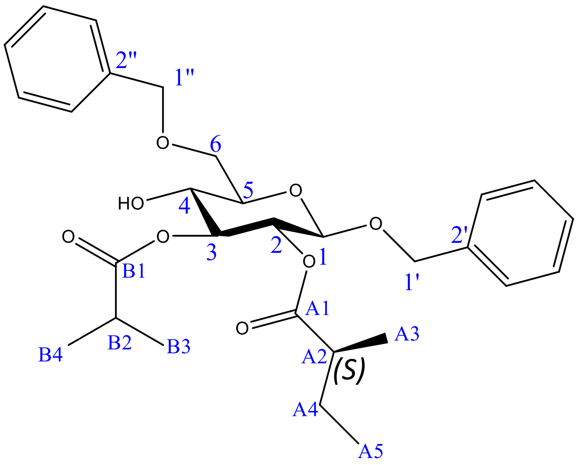

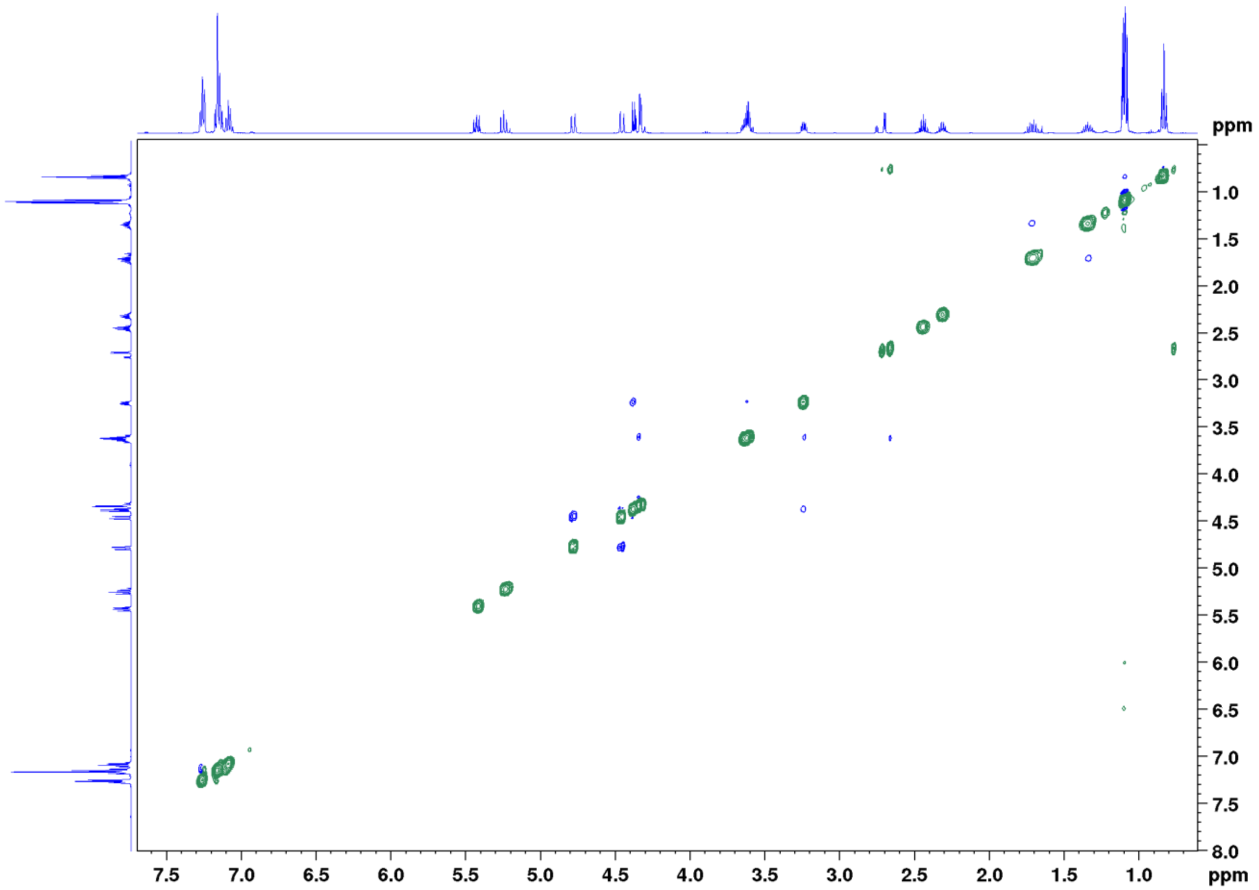

Figure S43: NOESY spectrum of compound **9** (500 MHz, C_6D_6).

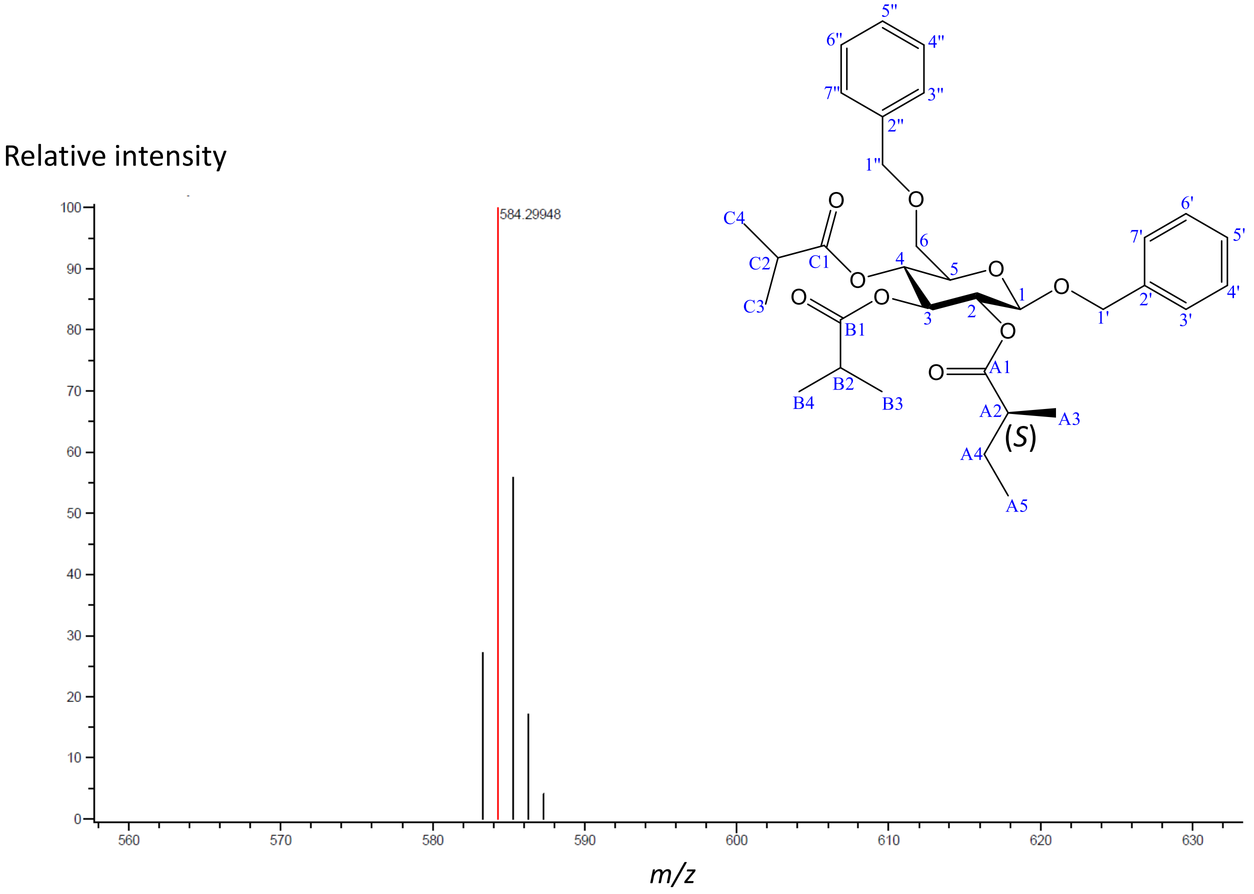

Figure S44: HRFD-MS spectrum of synthesized dibenzyl pennelliiside D (**2**).

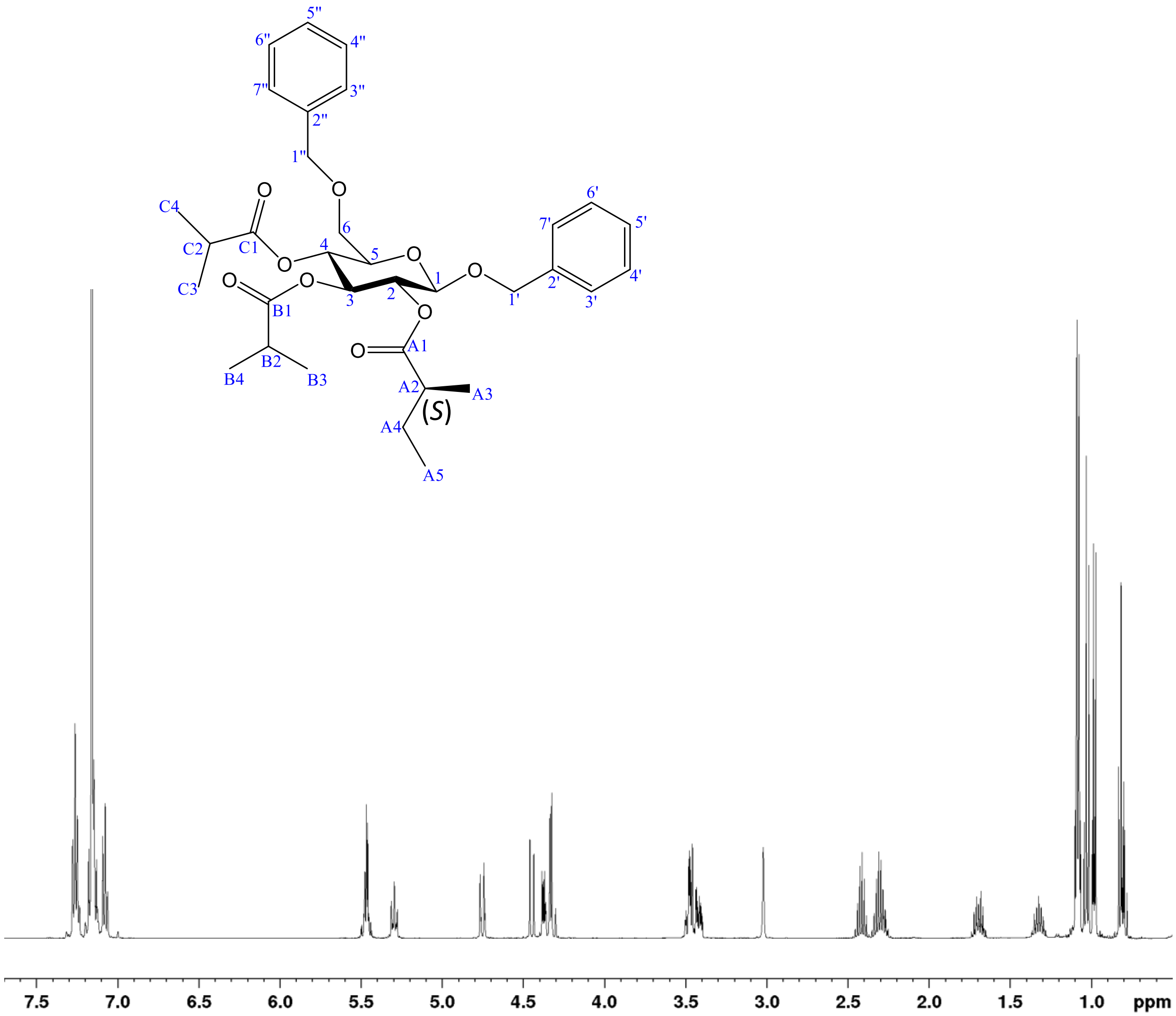

Figure S45: ^{1}H NMR spectrum of synthesized dibenzyl pennelliiside D (**2**) (500 MHz, C_6D_6).

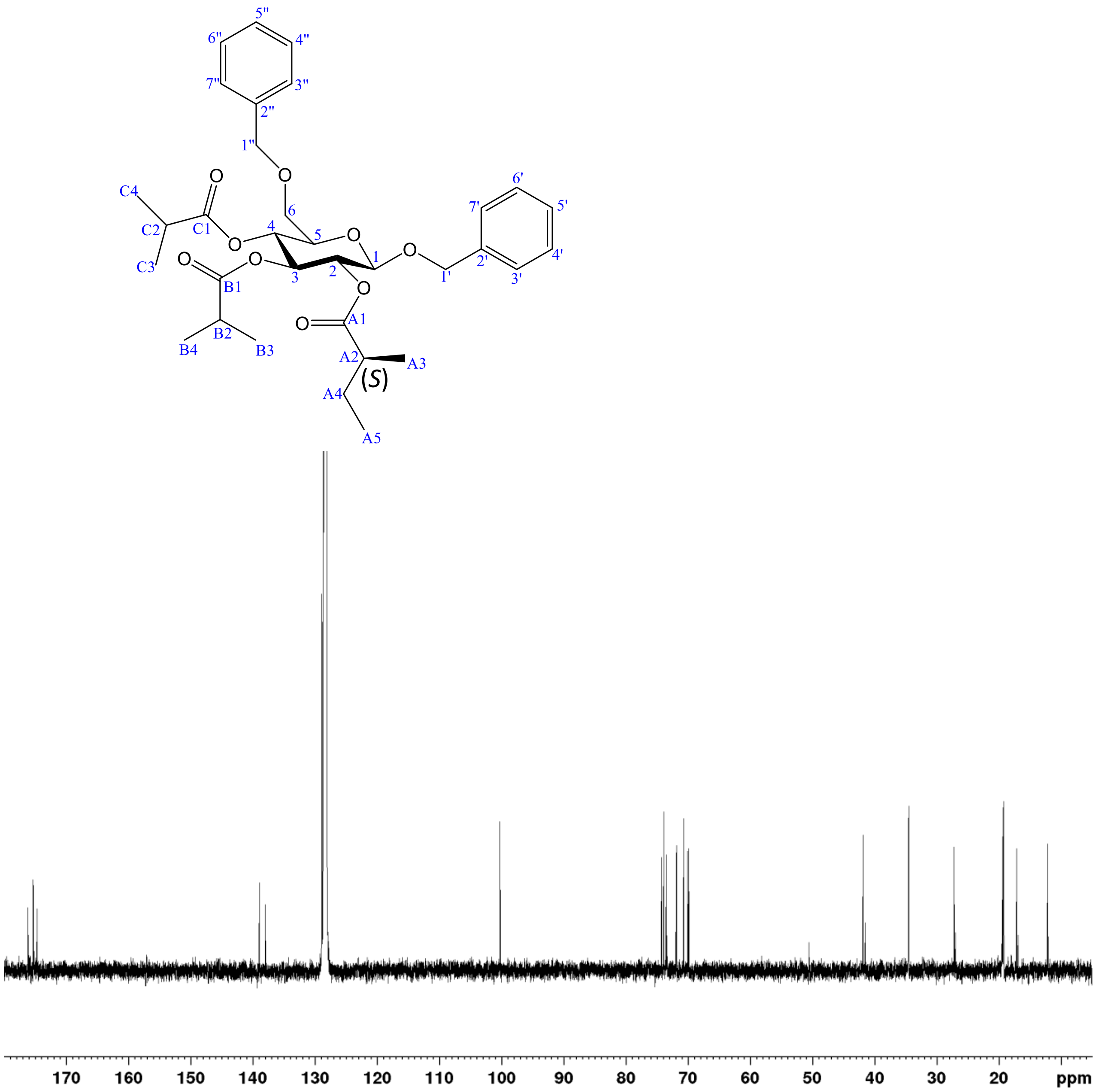

Figure S46: ^{13}C NMR spectrum of synthesized dibenzyl pennelliiside D (**2**) (126 MHz, C_6D_6).

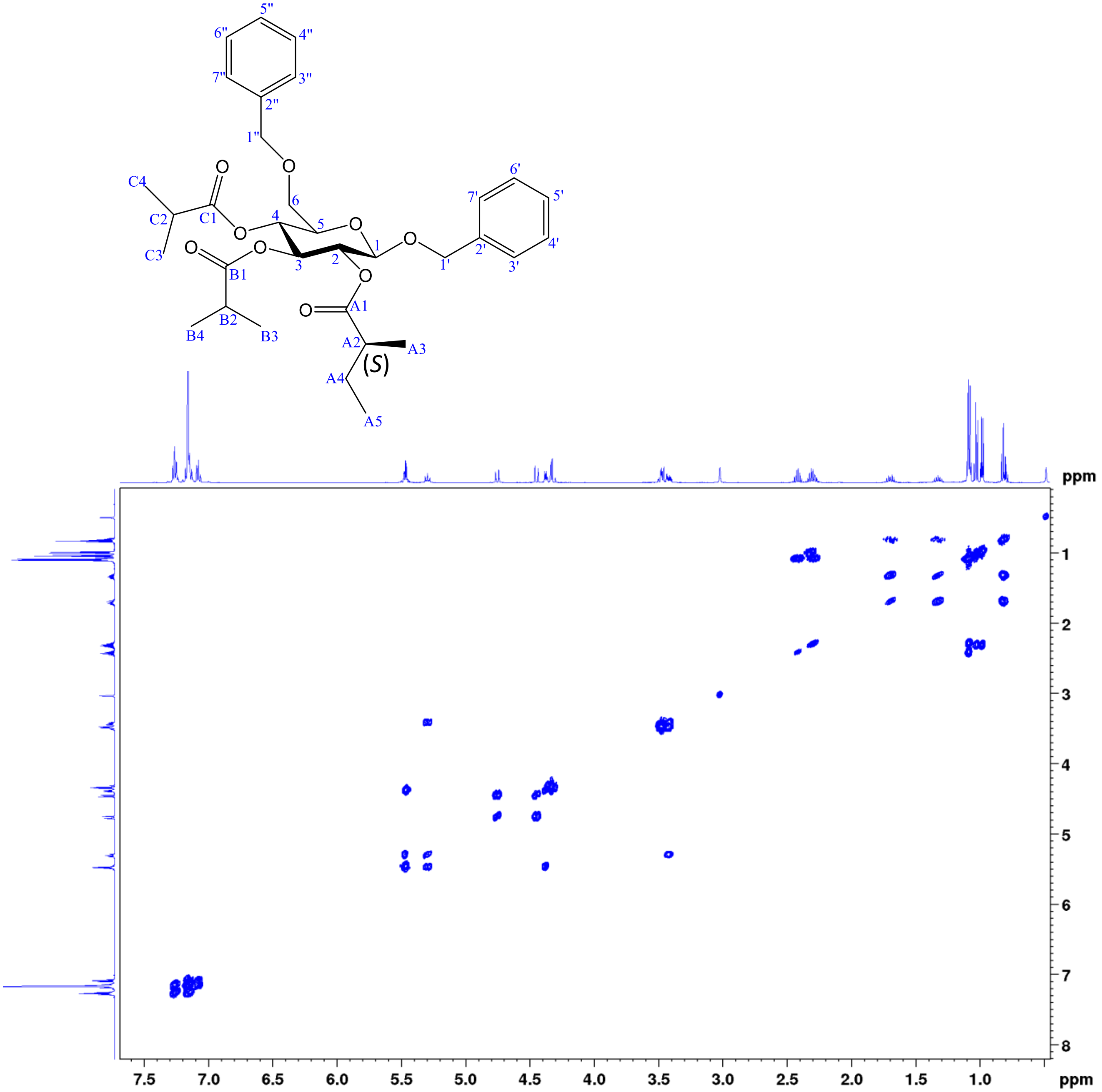

Figure S47: COSY spectrum of synthesized dibenzyl pennelliiside D (**2**) (500 MHz, C_6D_6).

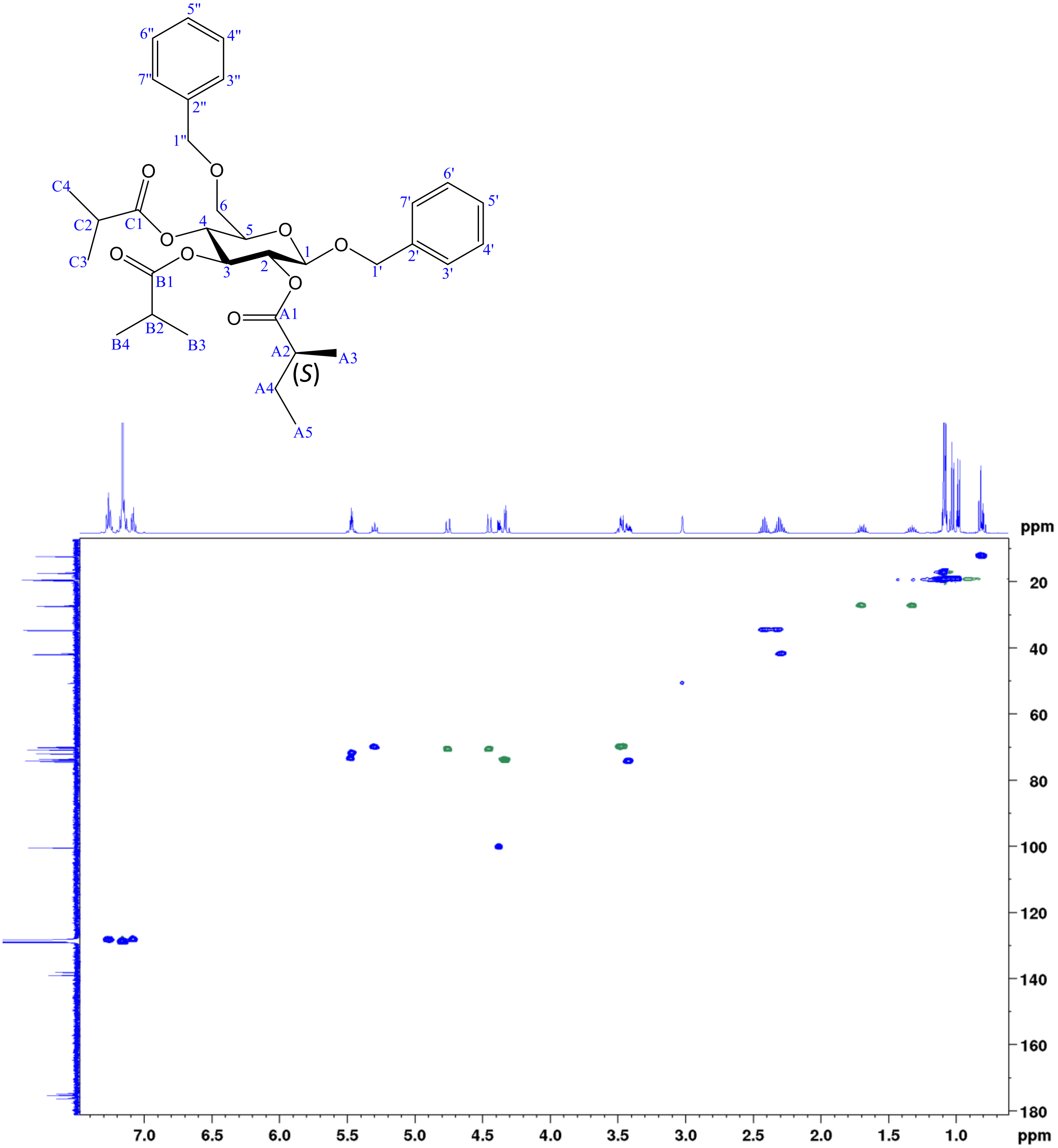

Figure S48: HSQC spectrum of synthesized dibenzyl pennelliiside D (**2**) (500 MHz, C_6D_6).

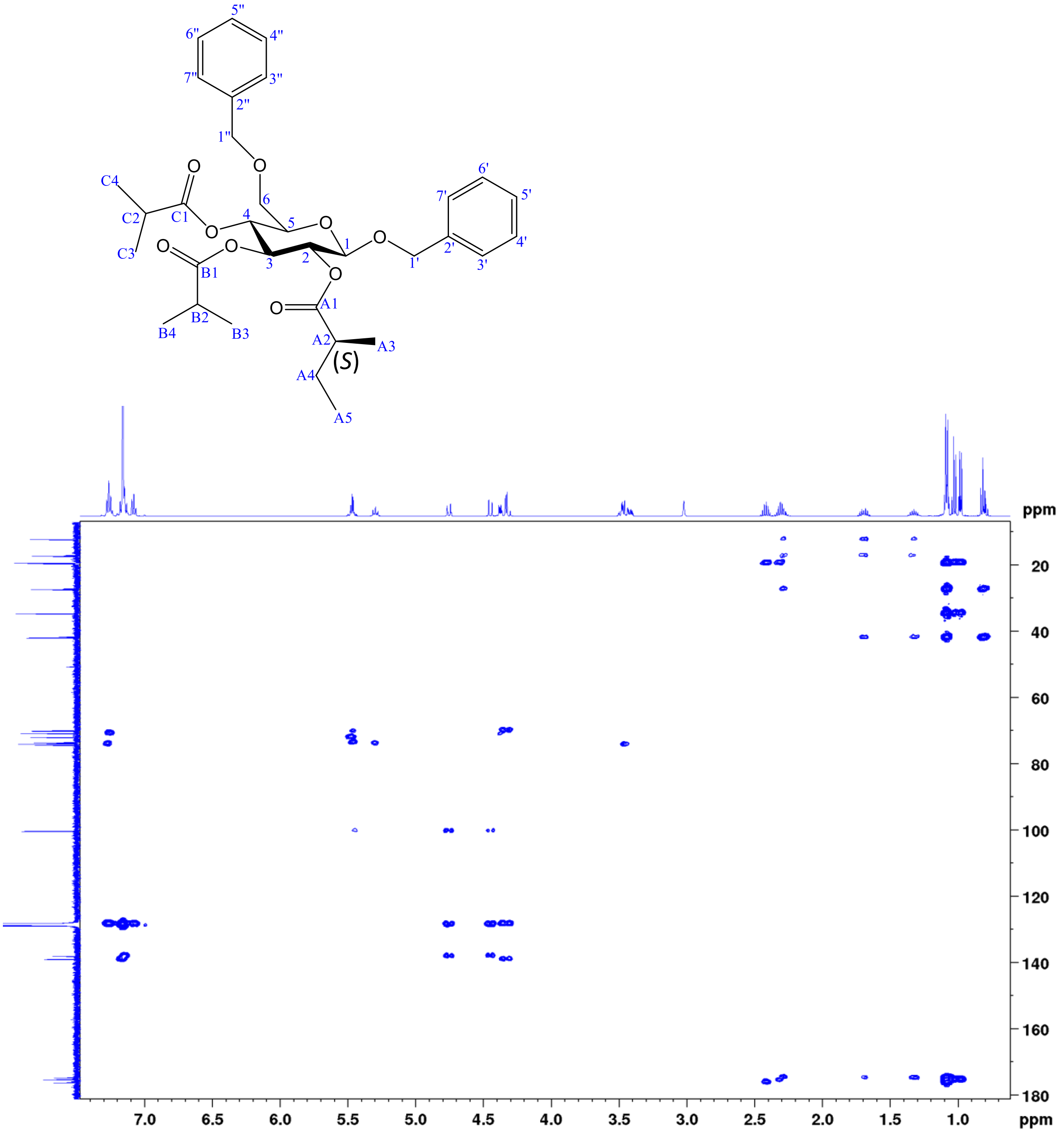

Figure S49: HMBC spectrum of synthesized dibenzyl pennelliiside D (**2**) (500 MHz, C_6D_6).

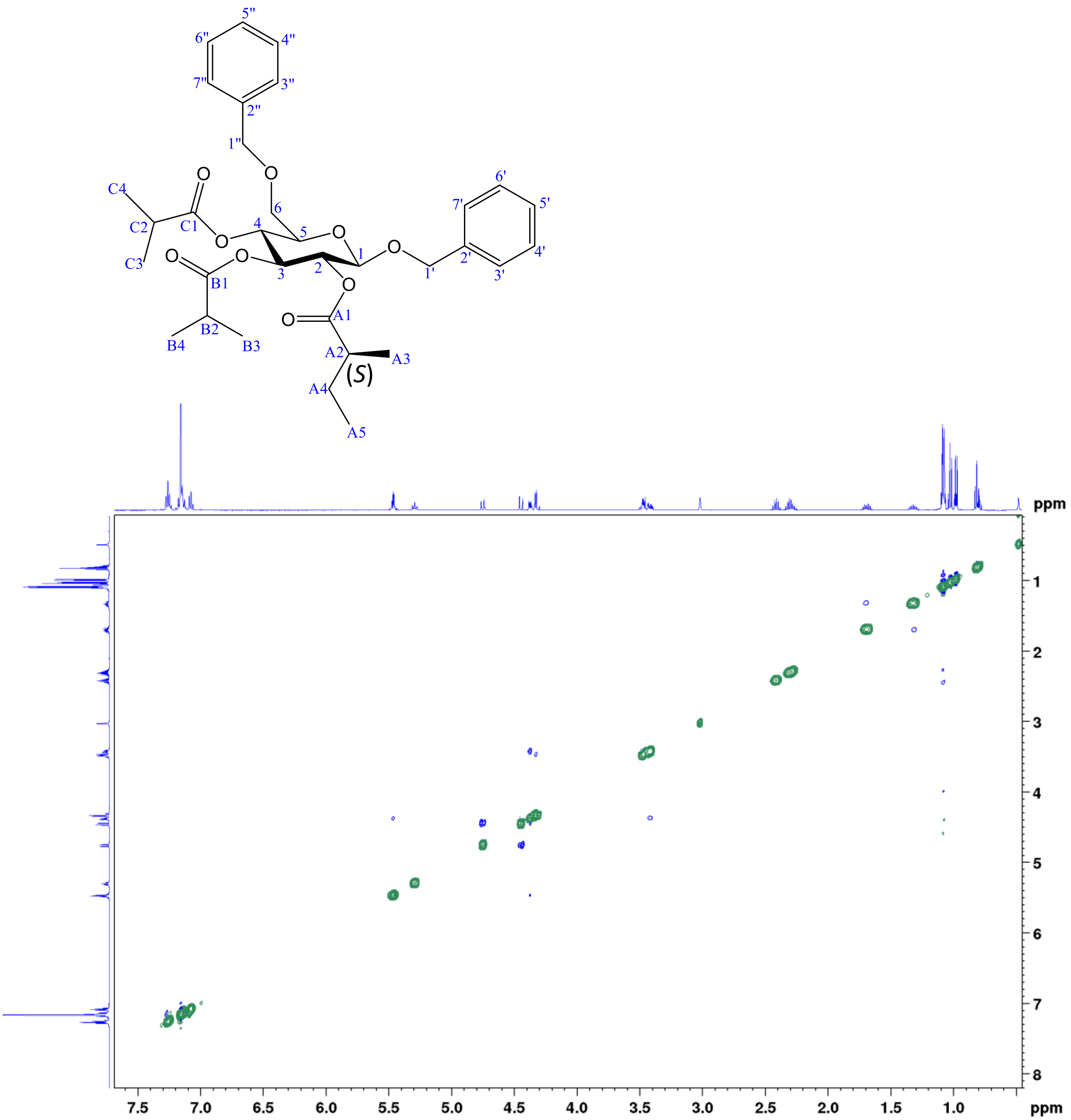

Figure S50: NOESY spectrum of synthesized dibenzyl pennelliiside D (**2**) (500 MHz, C_6D_6).

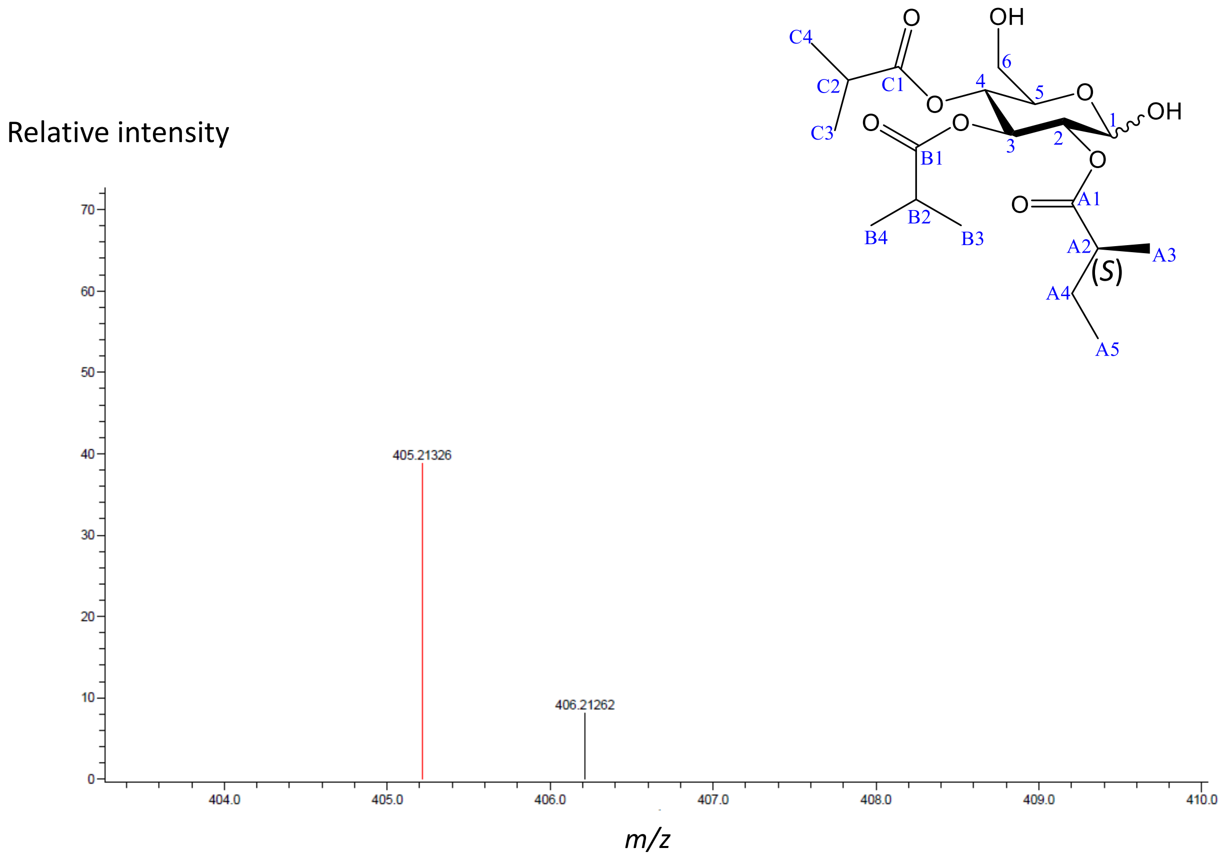

Figure S51: HRFD-MS spectrum of synthesized pennelliiside D (**1**).

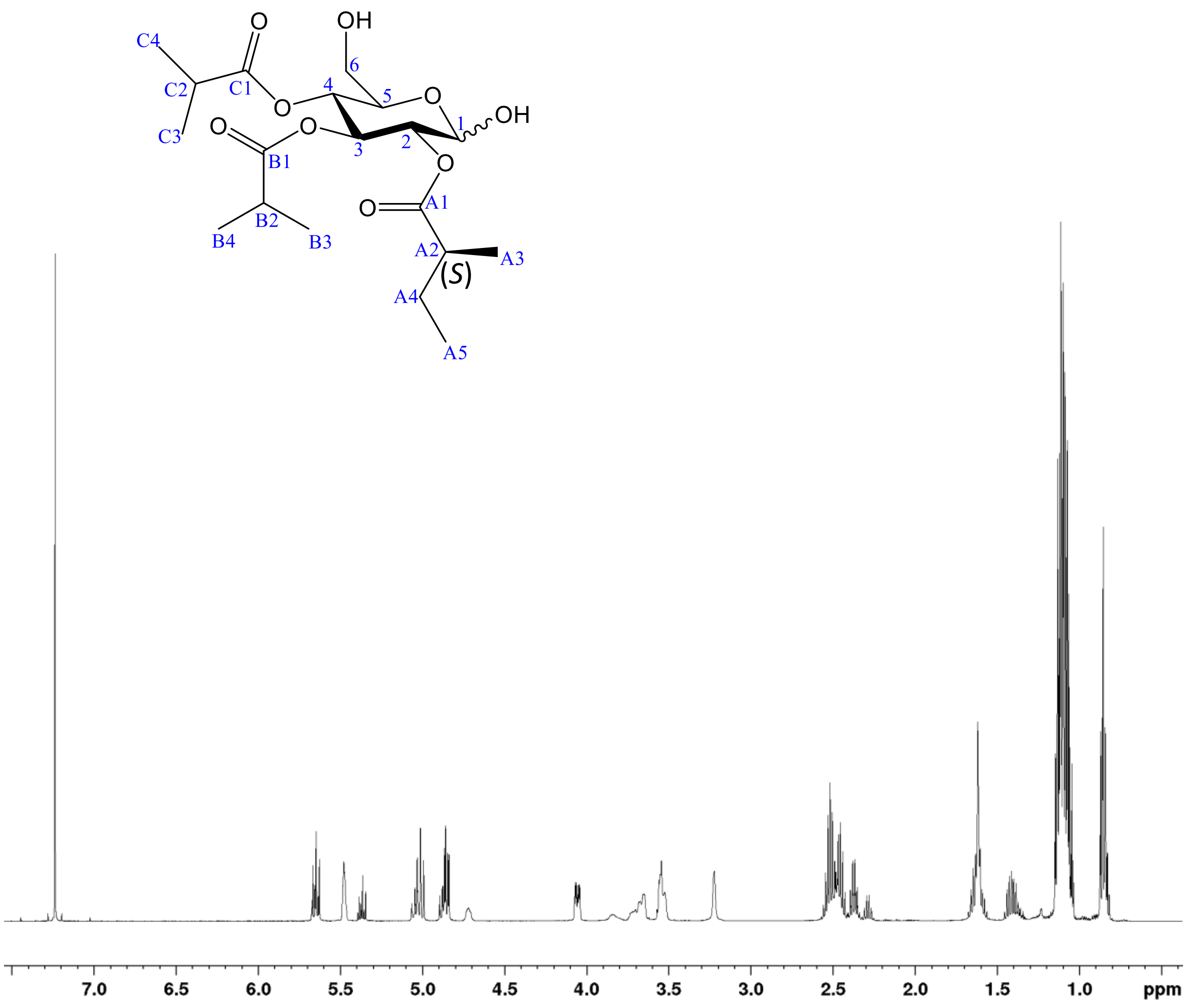

Figure S52: ^{1}H NMR spectrum of synthesized pennelliiside D (**1**) (500 MHz, $CDCl_3$).

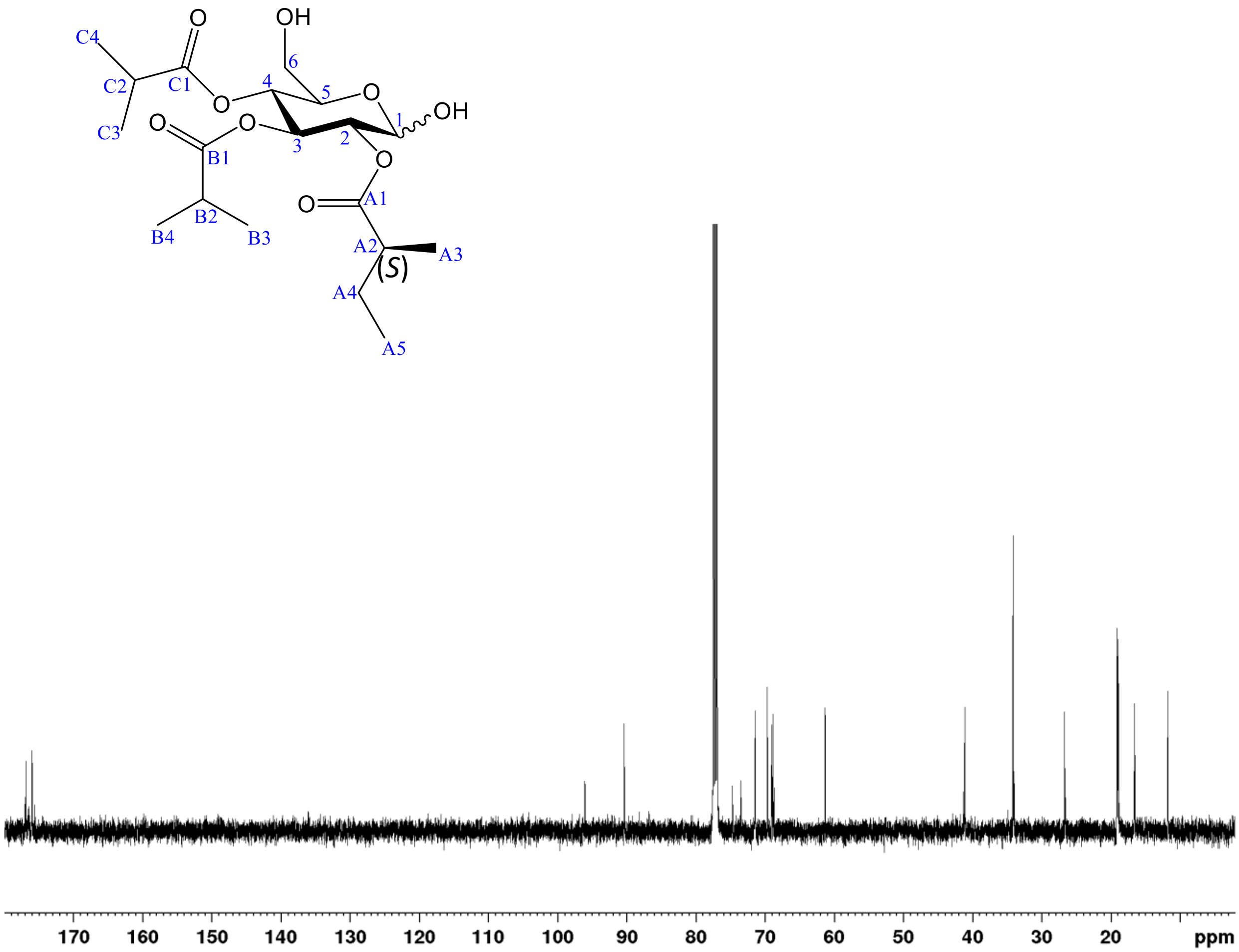

Figure S53: ^{13}C NMR spectrum of synthesized pennelliiside D (**1**) (126 MHz, $CDCl_3$).

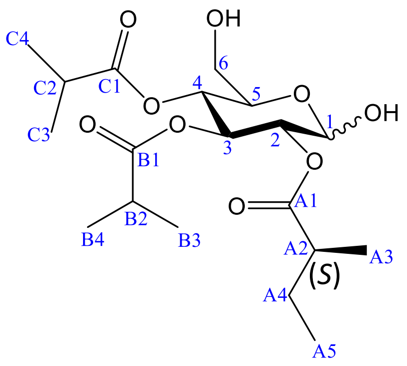

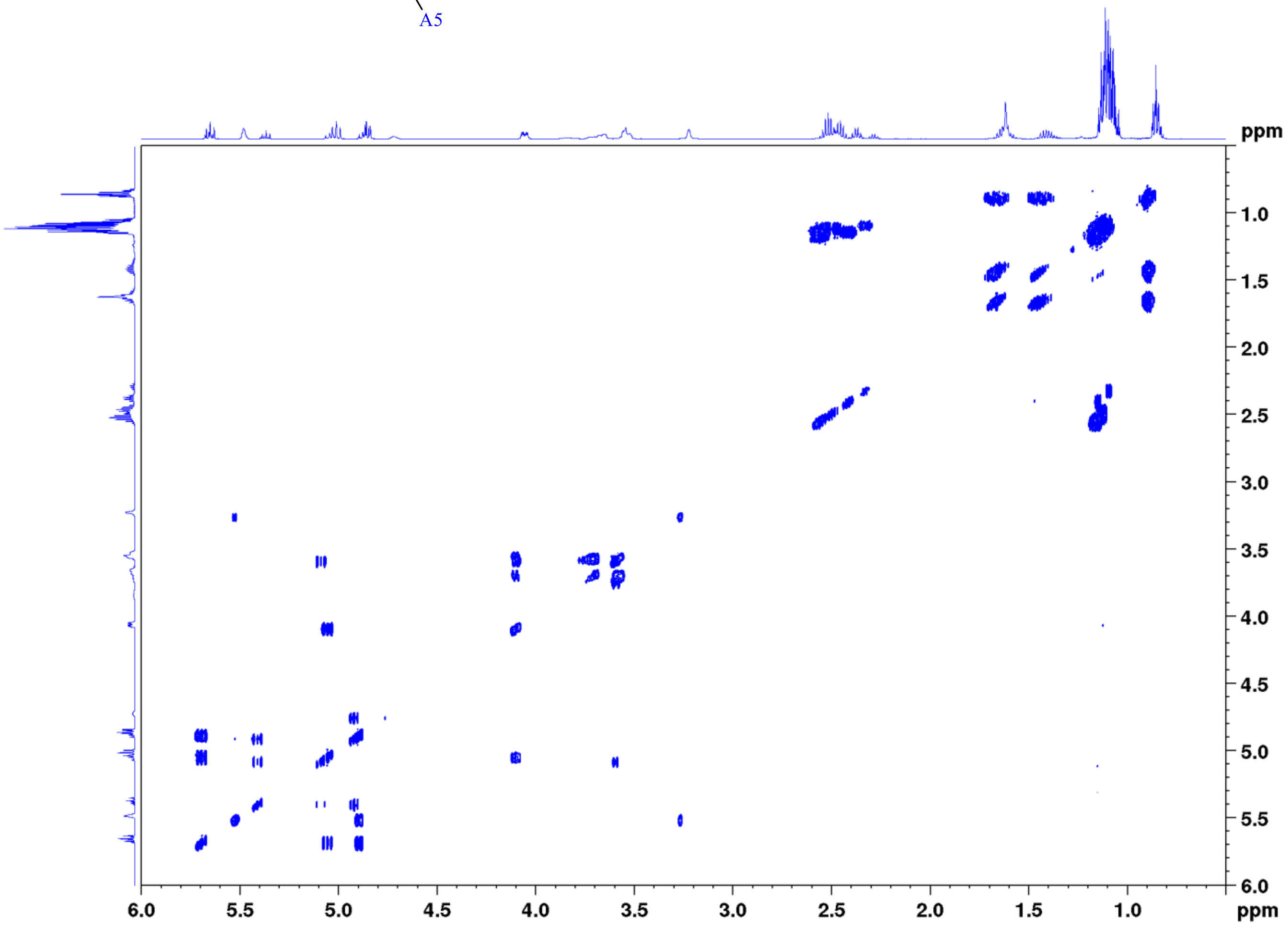

Figure S54: COSY spectrum of synthesized pennelliiside D (**1**) (500 MHz, $CDCl_3$).

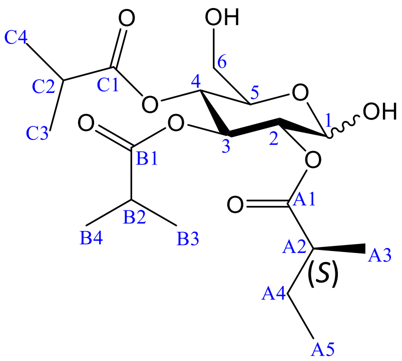

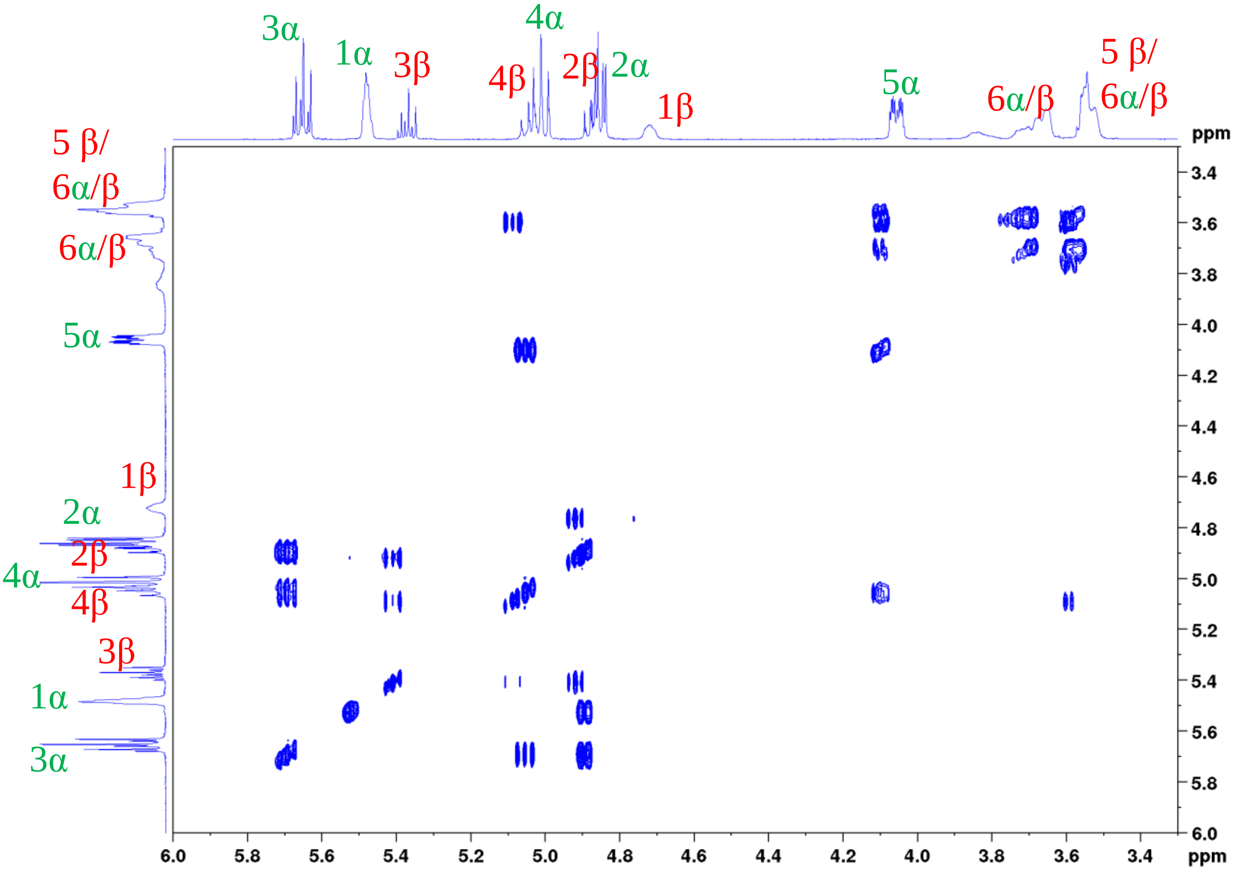

Figure S55: COSY spectrum of synthesized pennelliiside D (**1**) (500 MHz, $CDCl_3$).

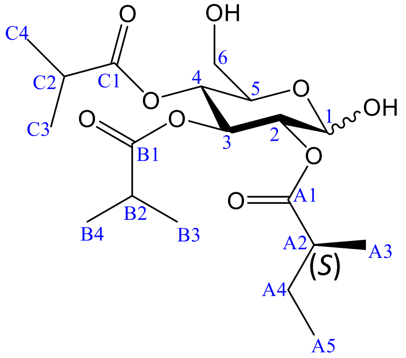

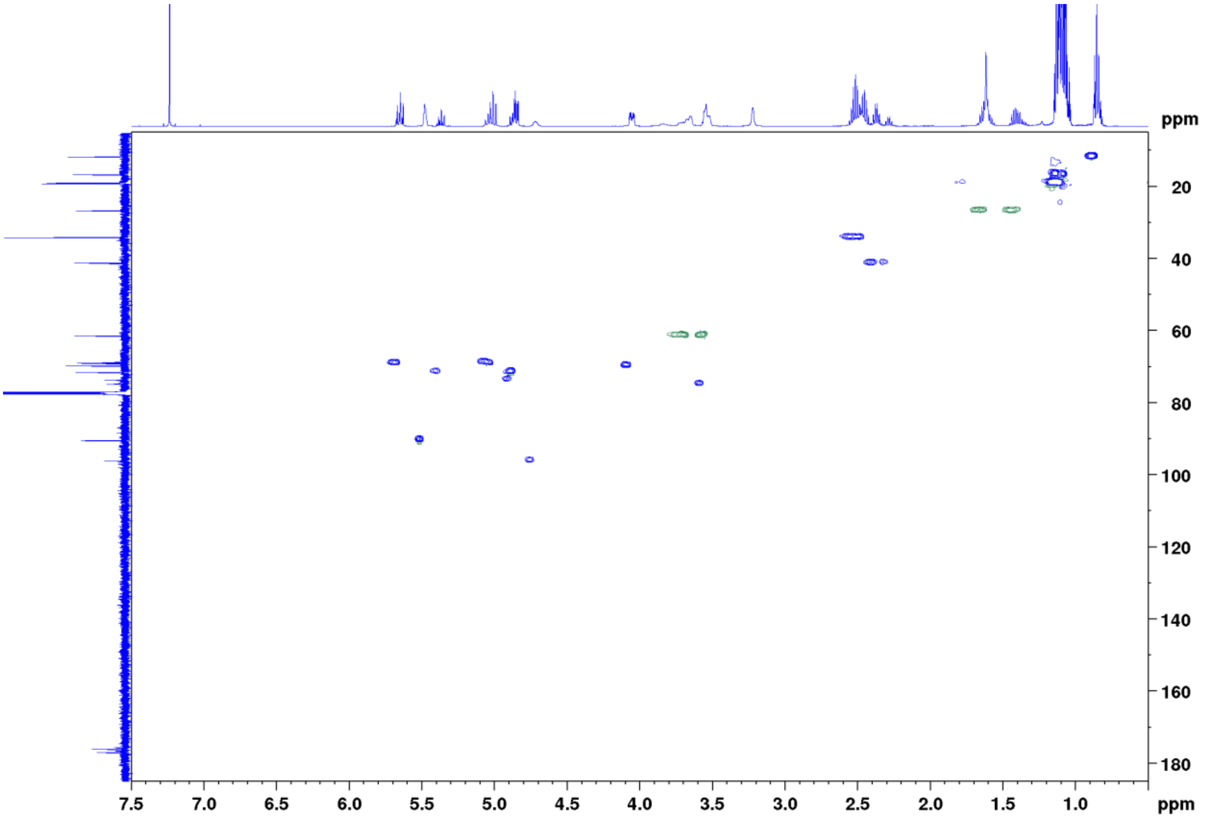

Figure S56: HSQC spectrum of synthesized pennelliiside D (**1**) (500 MHz, $CDCl_3$).

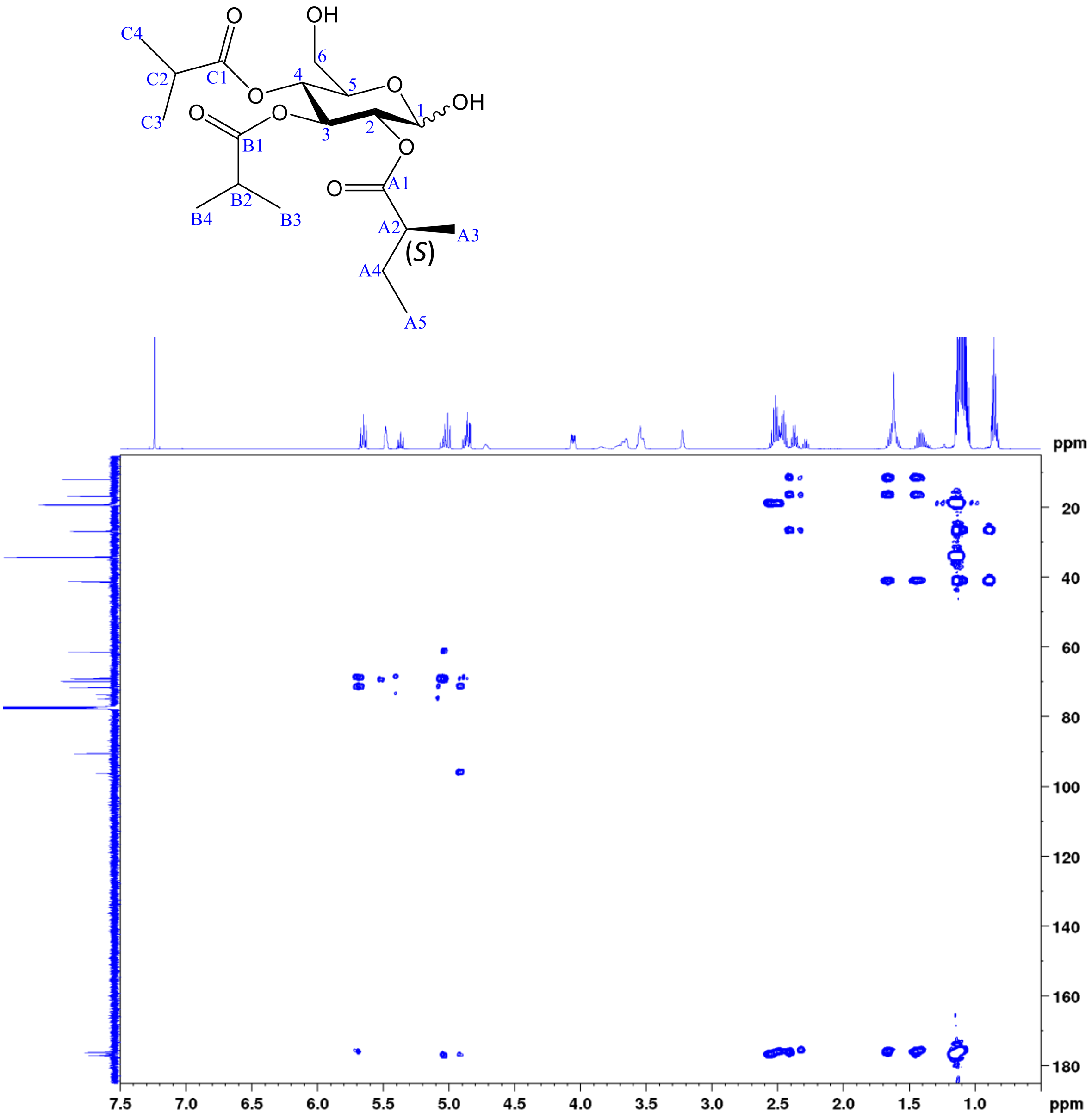

Figure S57: HMBC spectrum of synthesized pennelliiside D (**1**) (500 MHz, $CDCl_3$).

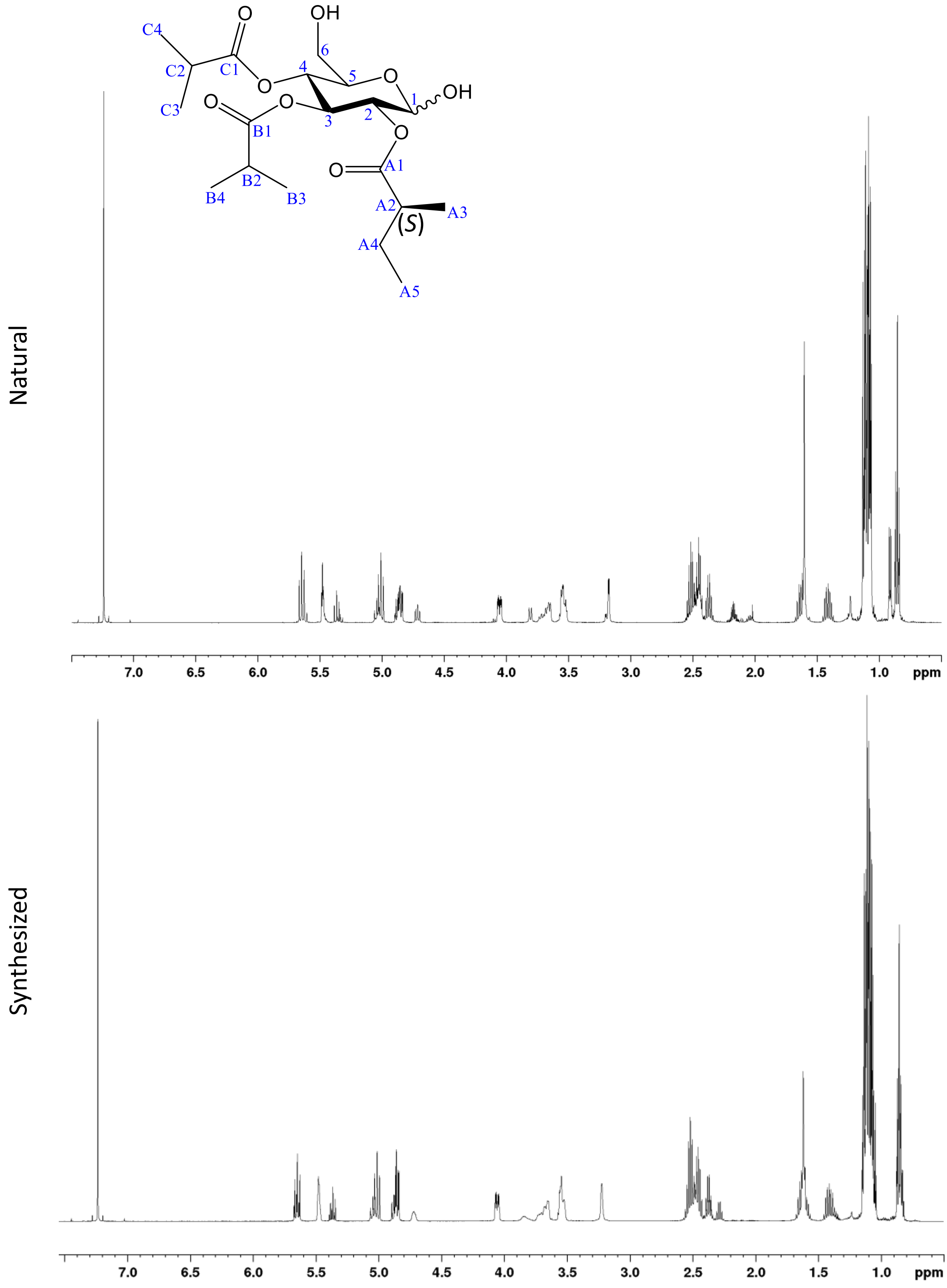

Figure S58: ^{1}H NMR spectra of natural and synthesized pennelliiside D (**1**) (500 MHz, $CDCl_3$).

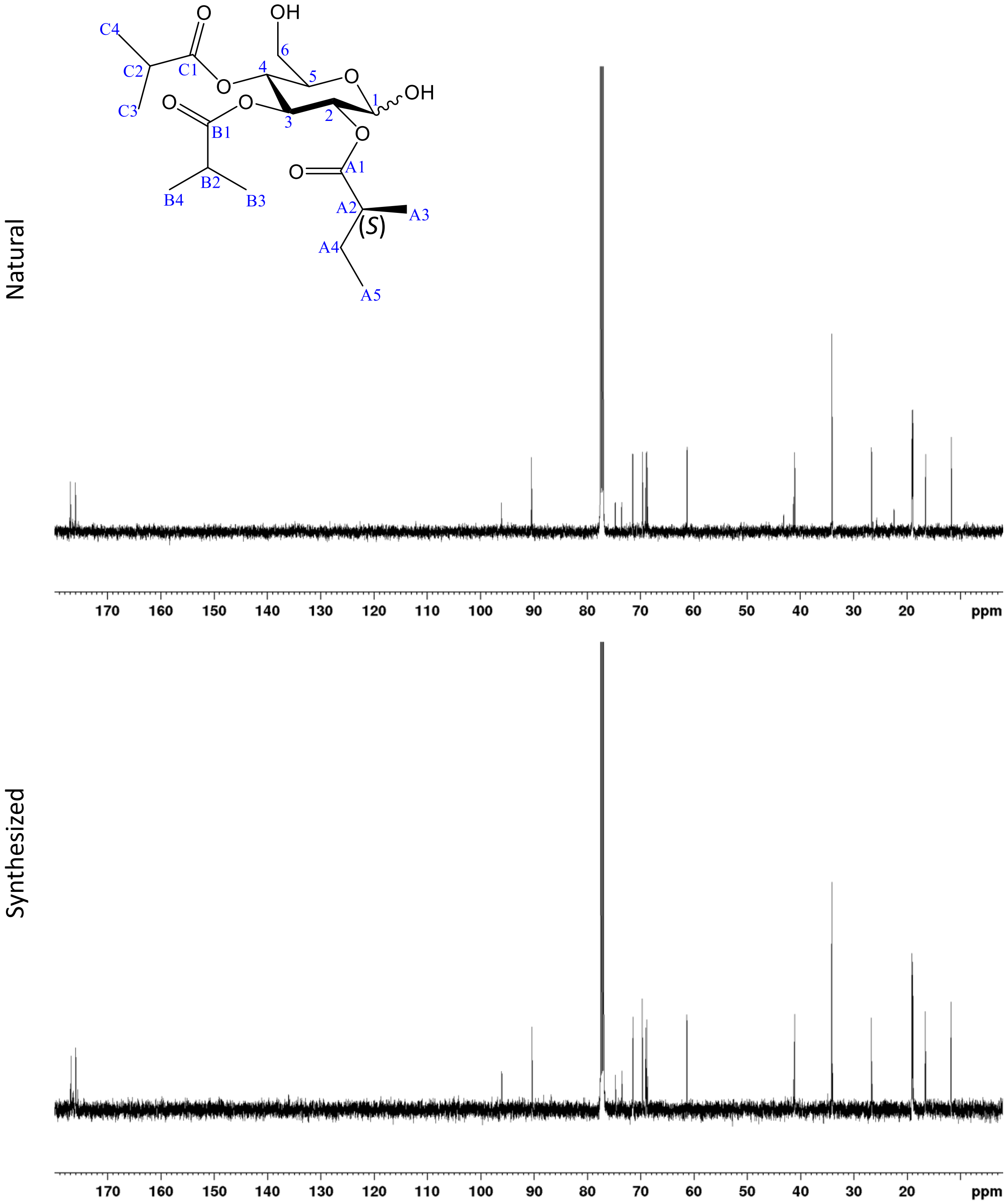

Figure S59: ^{13}C NMR spectrum of natural and synthesized pennelliiside D (**1**) (126 MHz, $CDCl_3$).

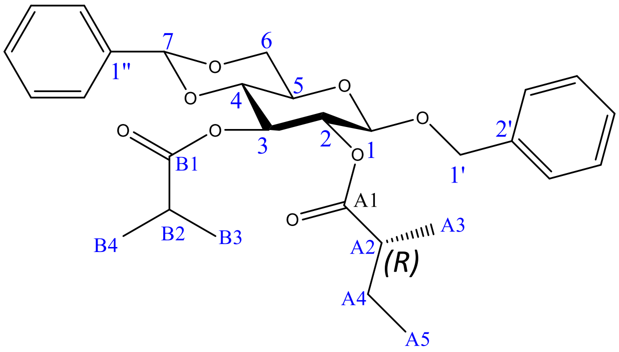

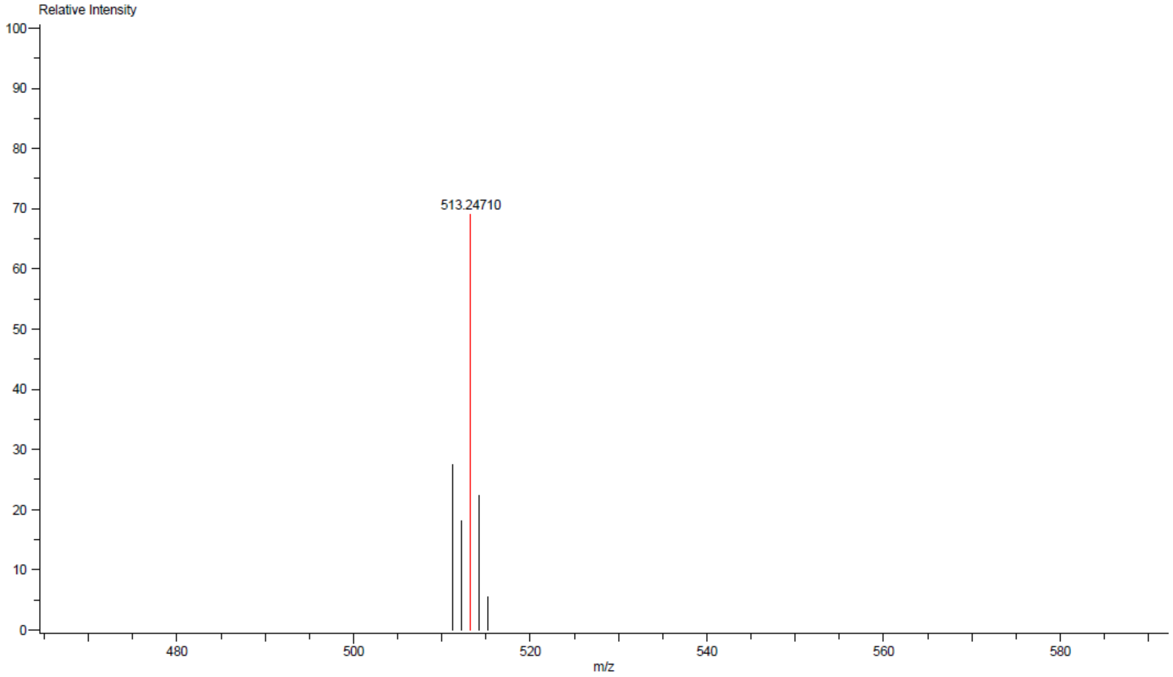

Figure S60: HRFD-MS spectrum of compound **10**.

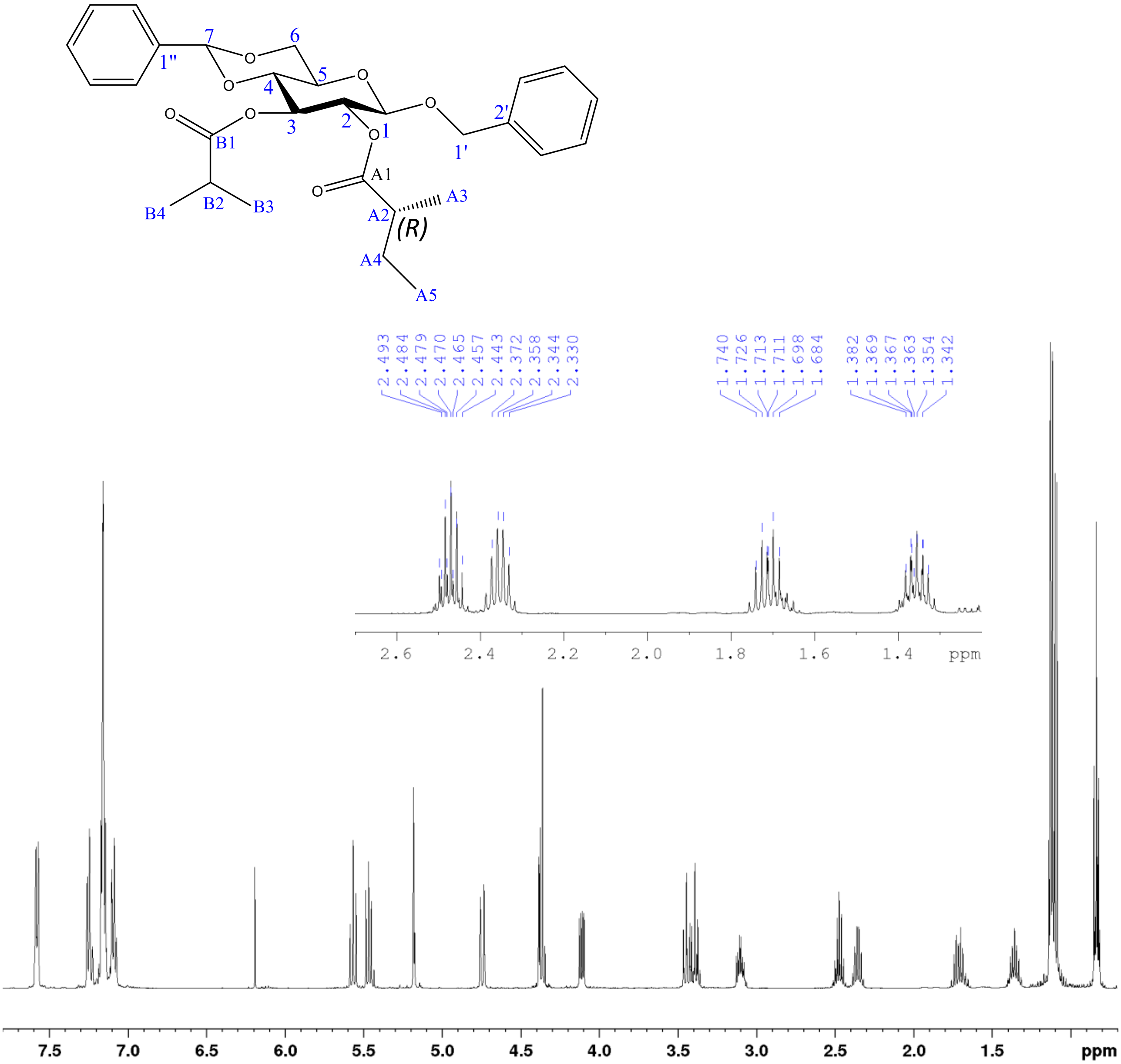

Figure S61: ^{1}H NMR spectrum of compound **10** (500 MHz, C_6D_6).

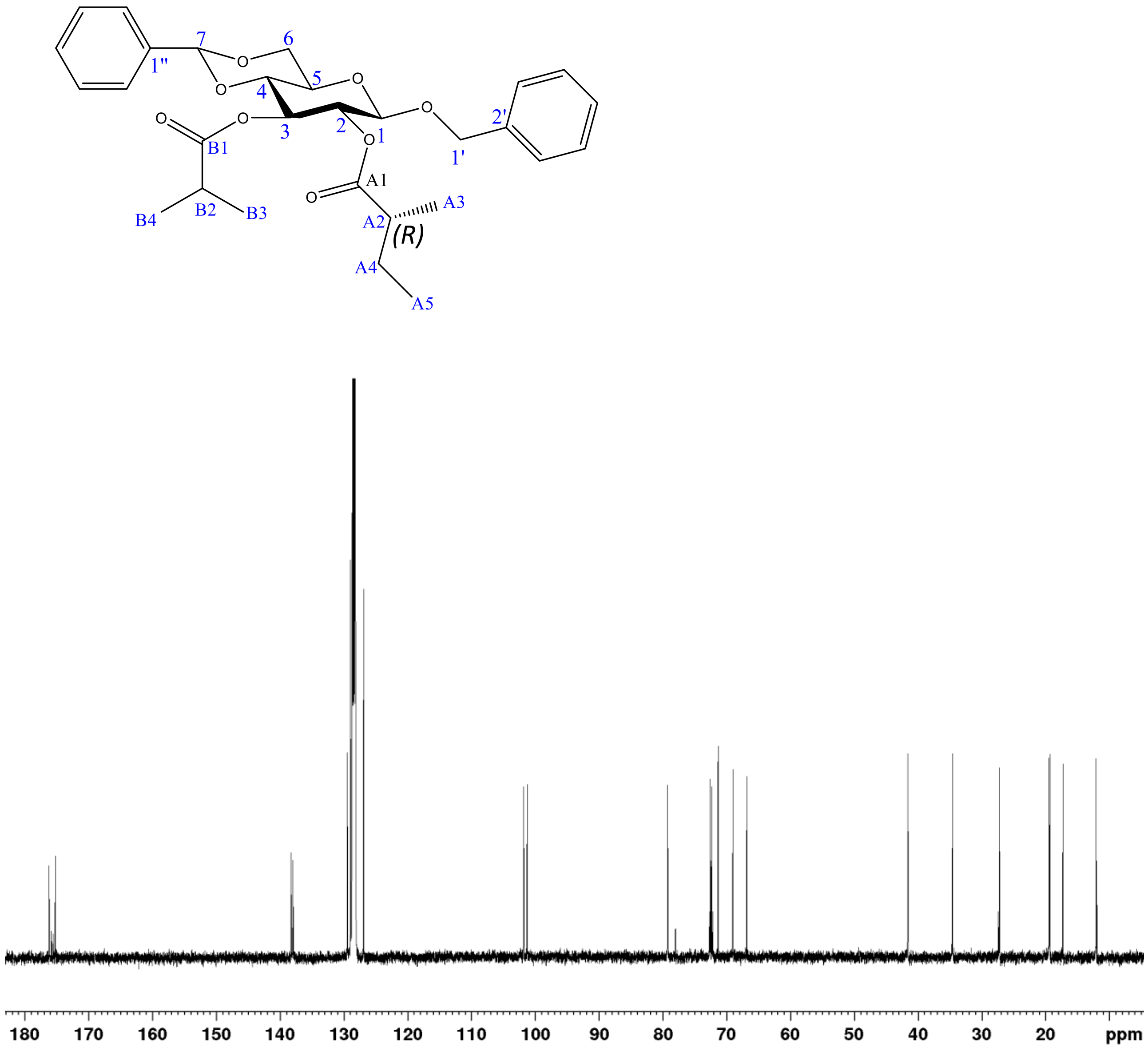

Figure S62: ^{13}C NMR spectrum of compound **10** (126 MHz, C_6D_6).

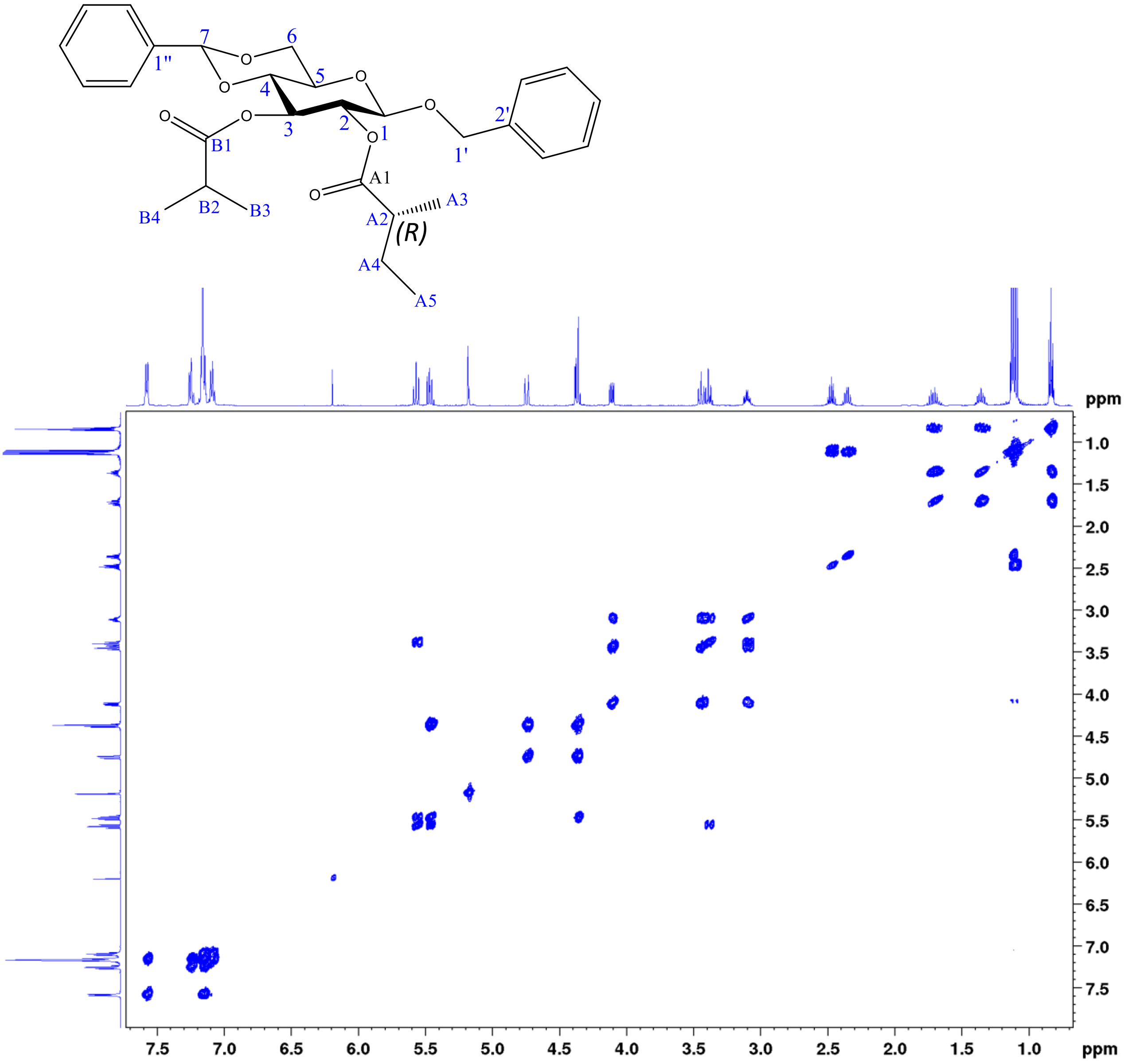

Figure S63: COSY spectrum of compound **10** (500 MHz, C_6D_6).

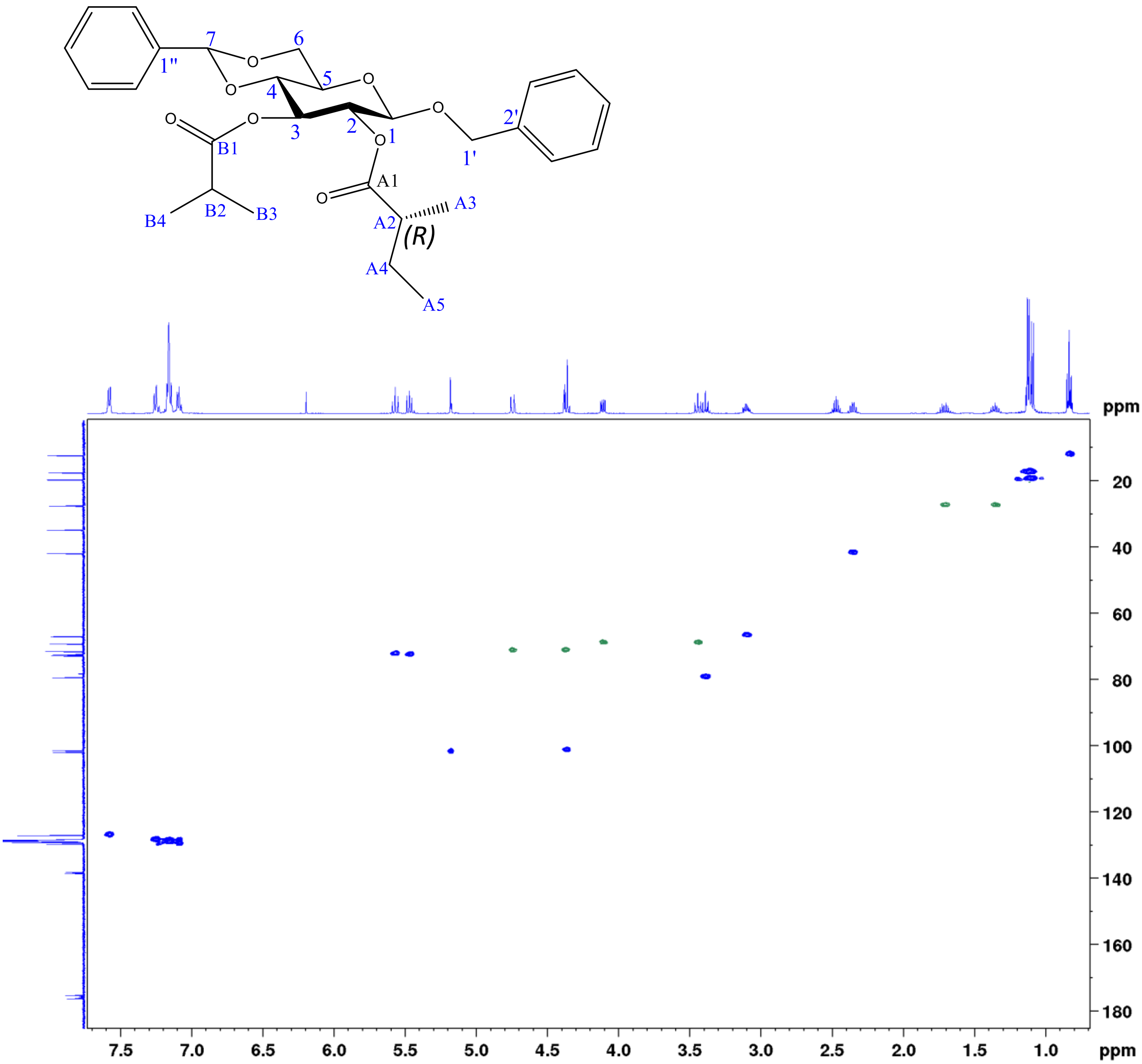

Figure S64: HSQC spectrum of compound **10** (500 MHz, C_6D_6).

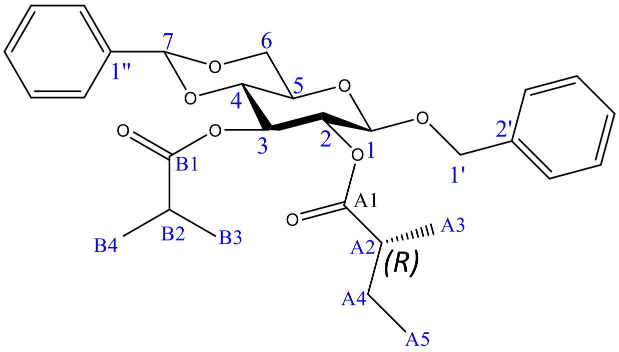

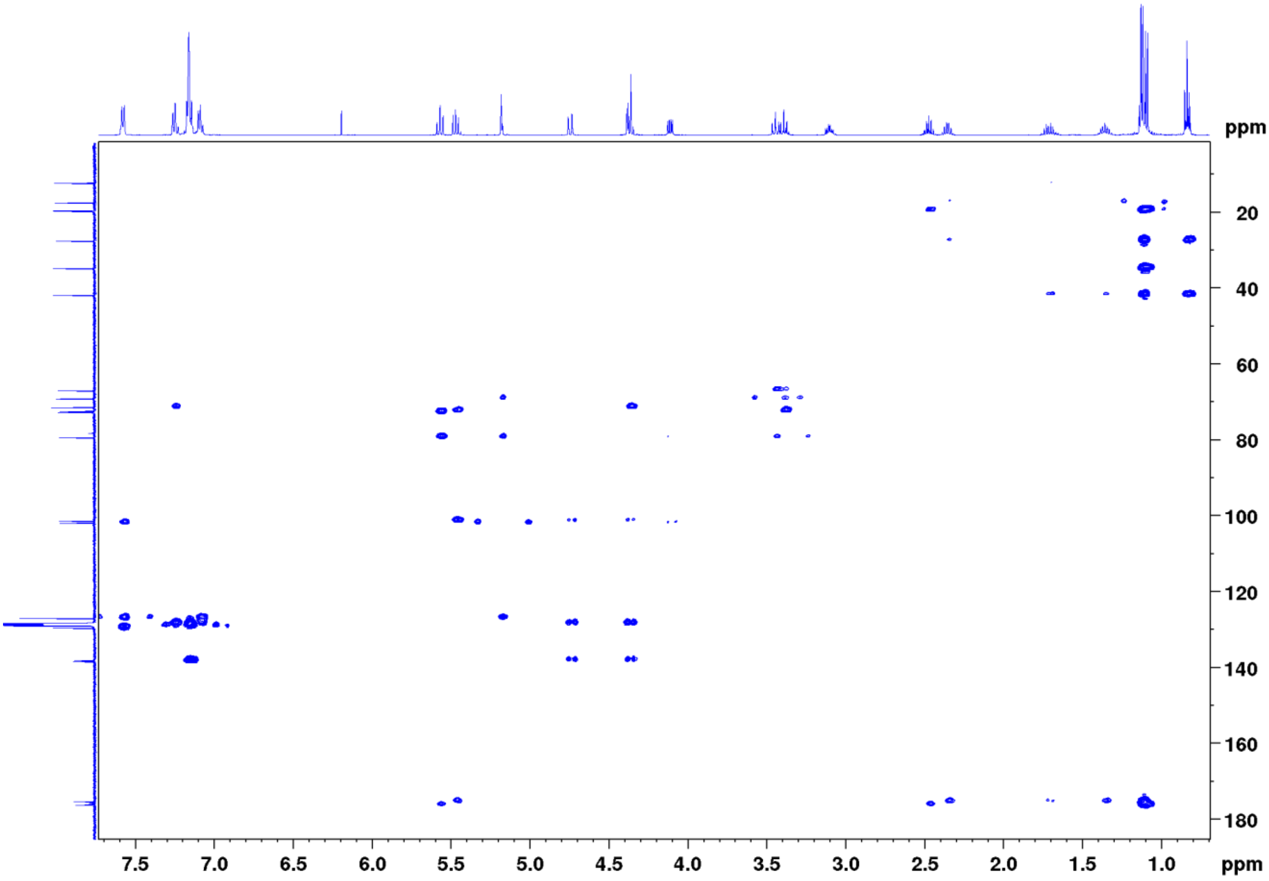

Figure S65: HMBC spectrum of compound **10** (500 MHz, C_6D_6).

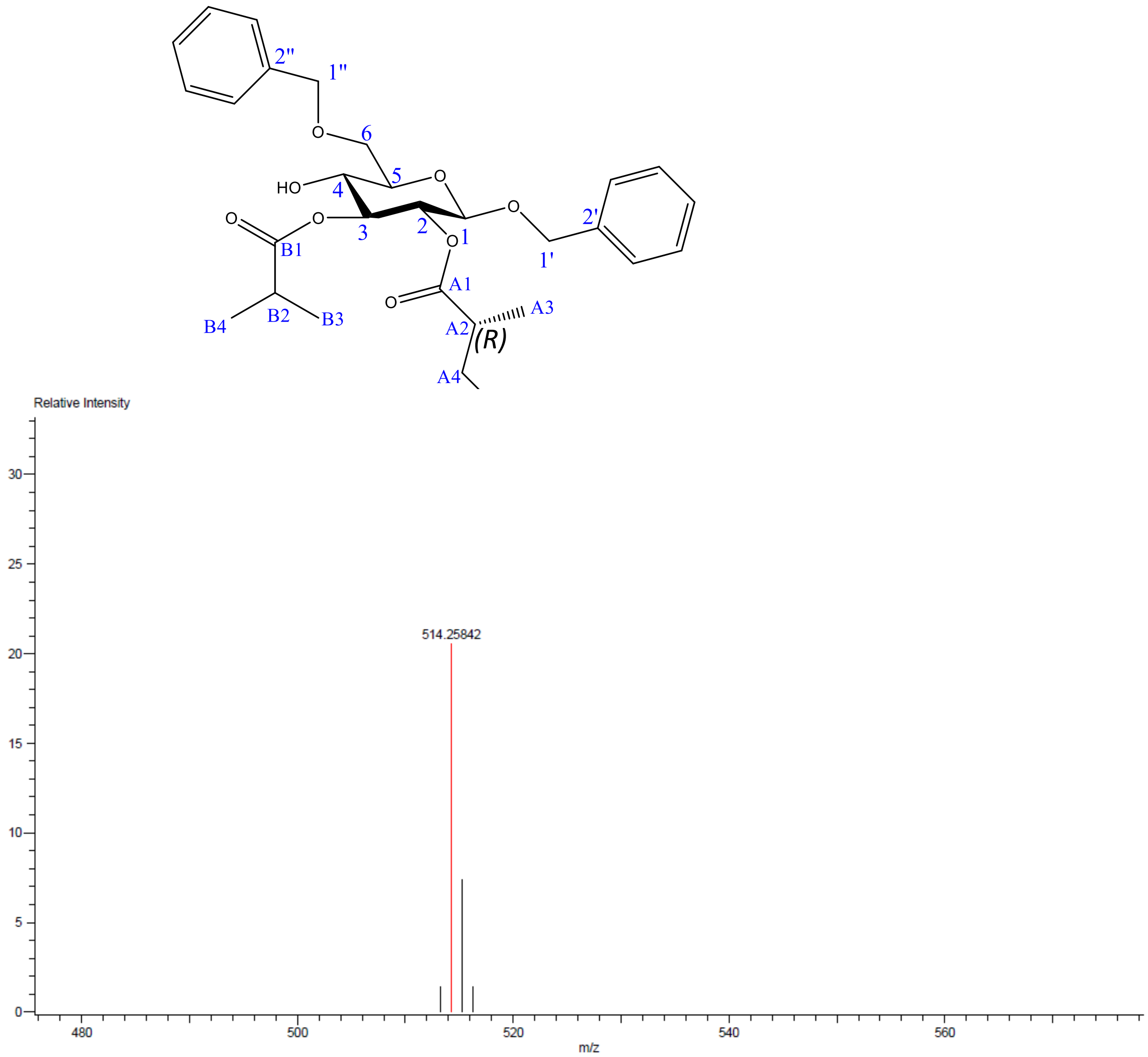

Figure S66: HRFD-MS spectrum of compound **11.**

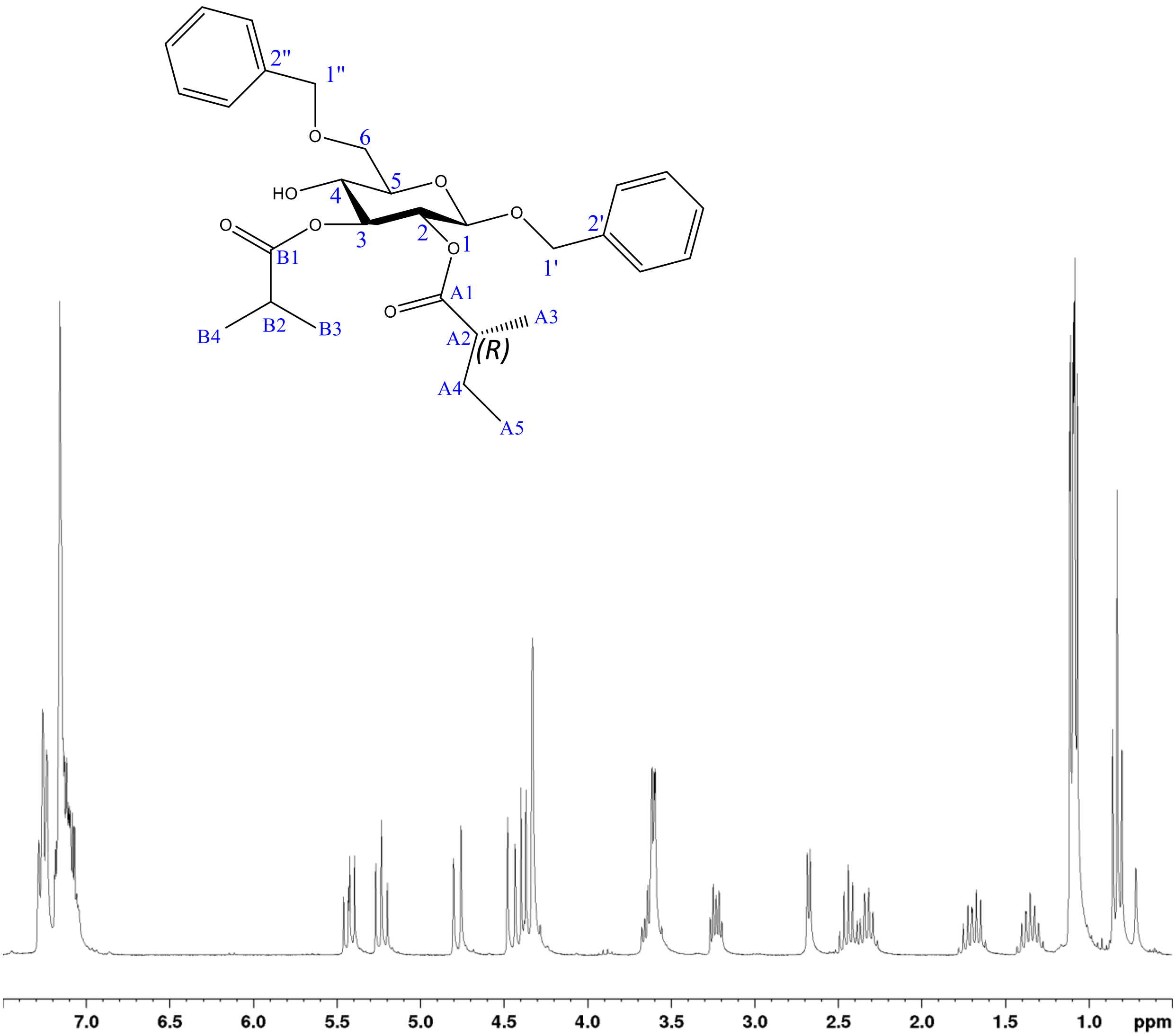

Figure S67: ^{1}H NMR spectrum of compound **11** (270 MHz, C_6D_6).

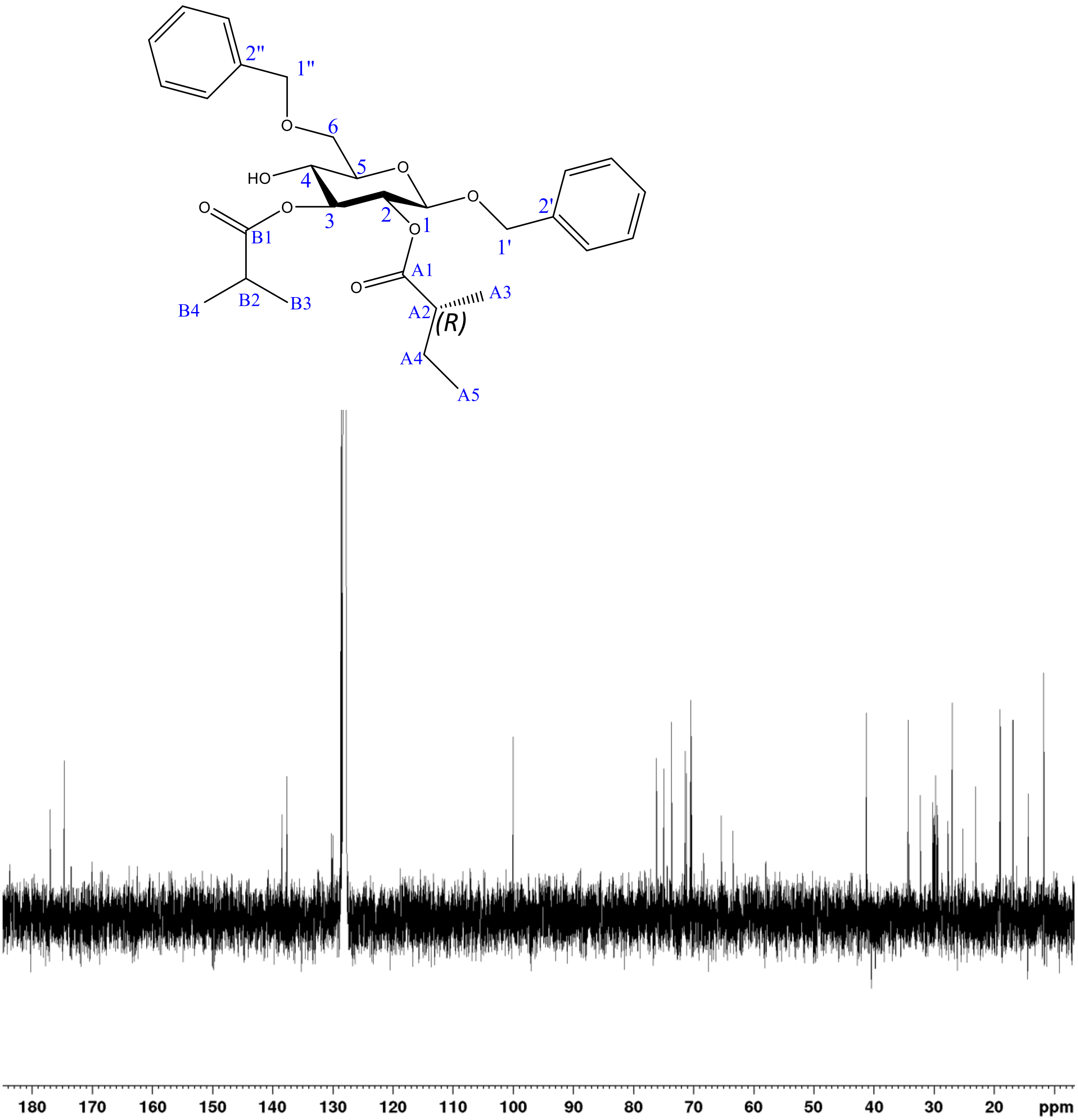

Figure S68: ^{13}C NMR spectrum of compound **11** (126 MHz, C_6D_6).

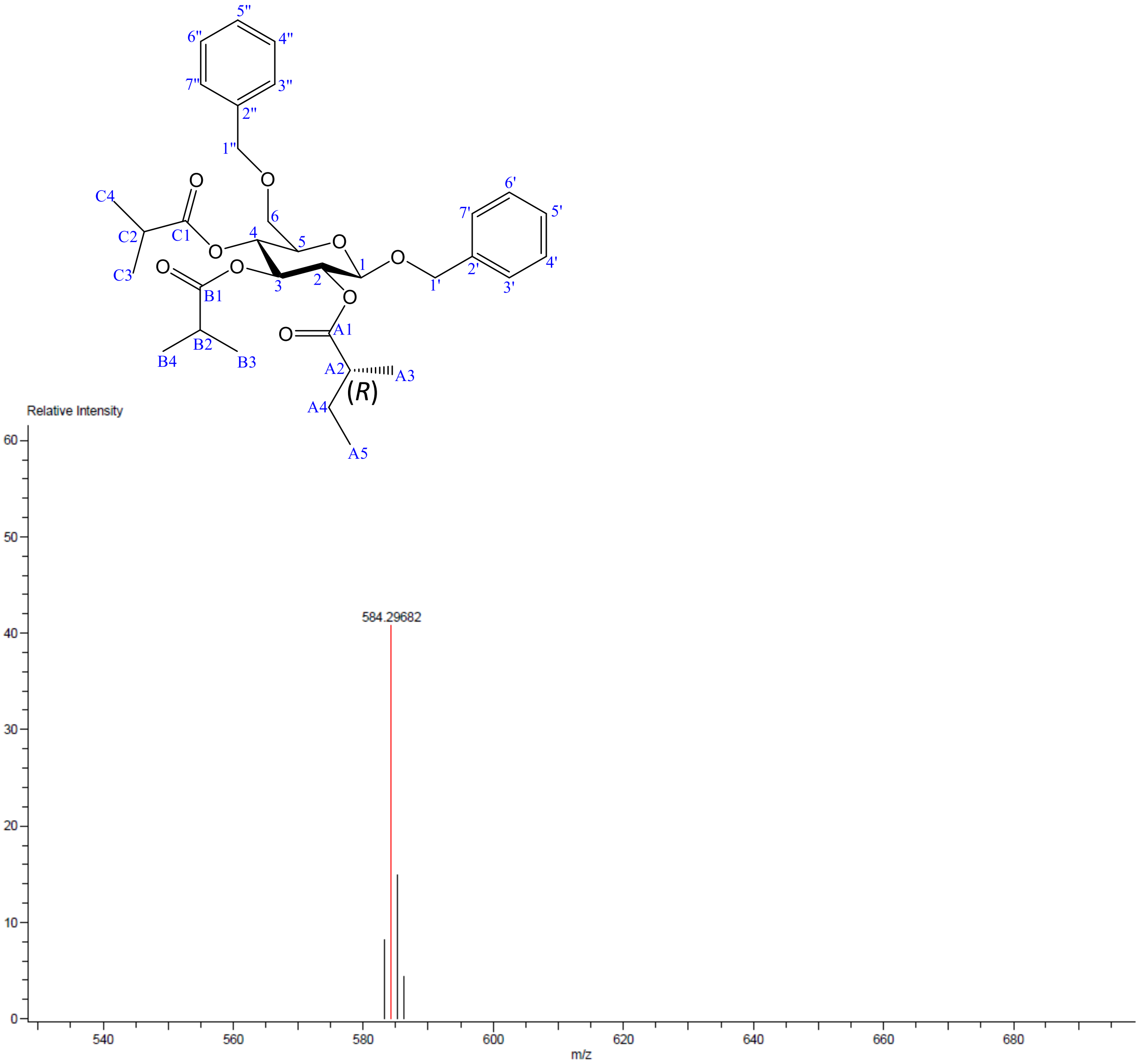

Figure S69: HRFD-MS spectrum of compound **12**.

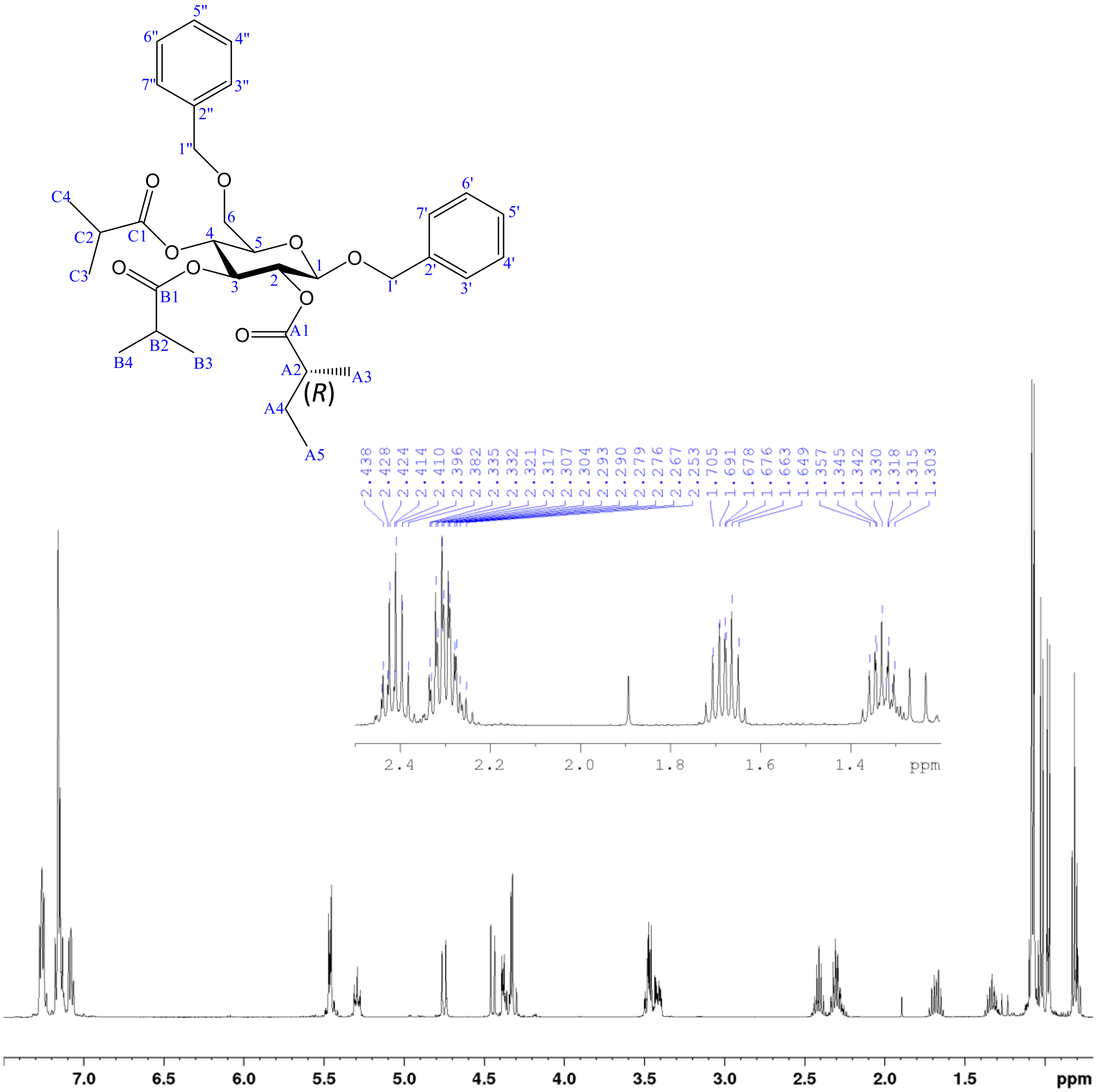

Figure S70: ^{1}H NMR spectrum of compound **12** (500 MHz, C_6D_6).

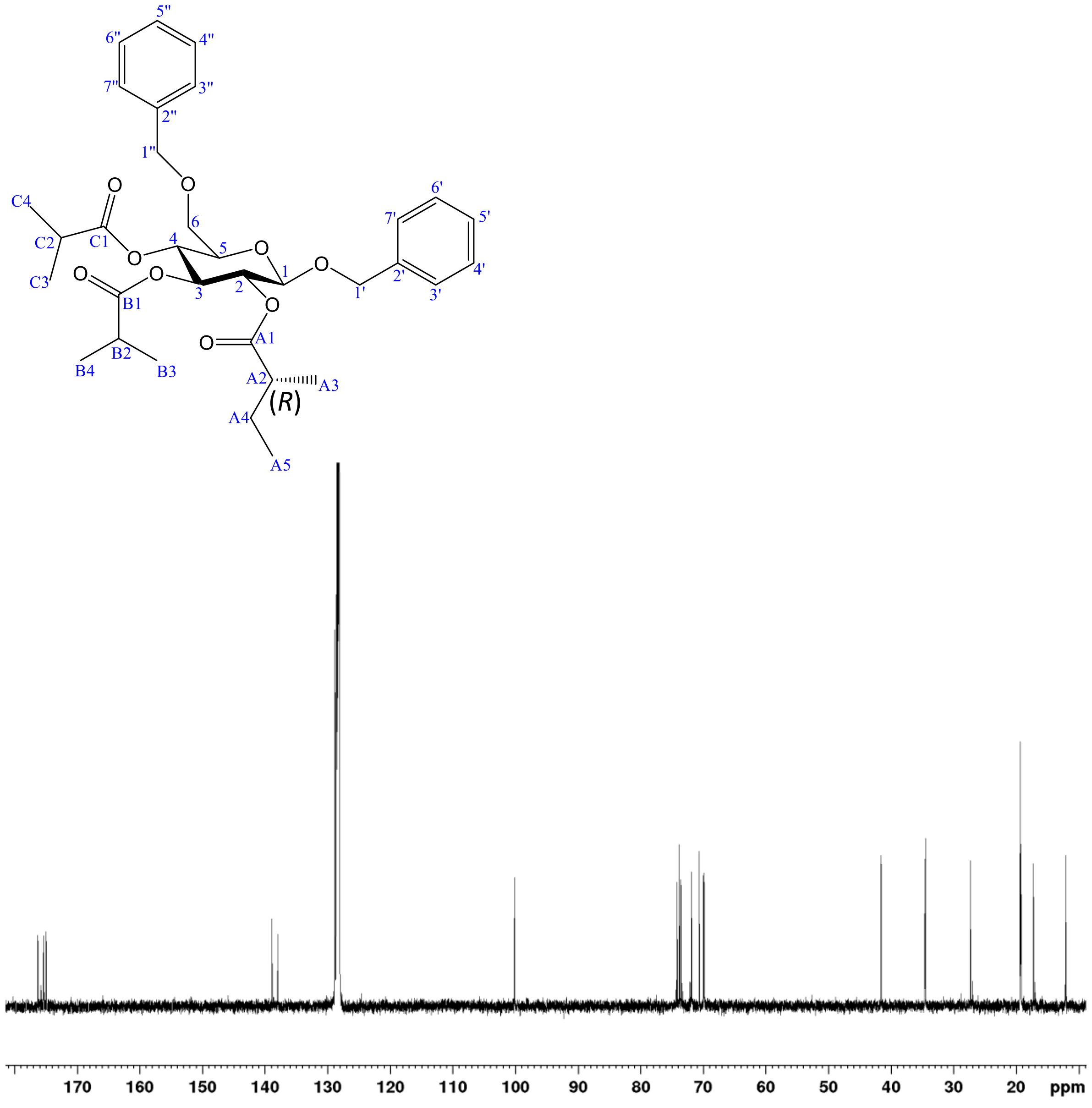

Figure S71: ^{13}C NMR spectrum of compound **12** (126 MHz, C_6D_6).

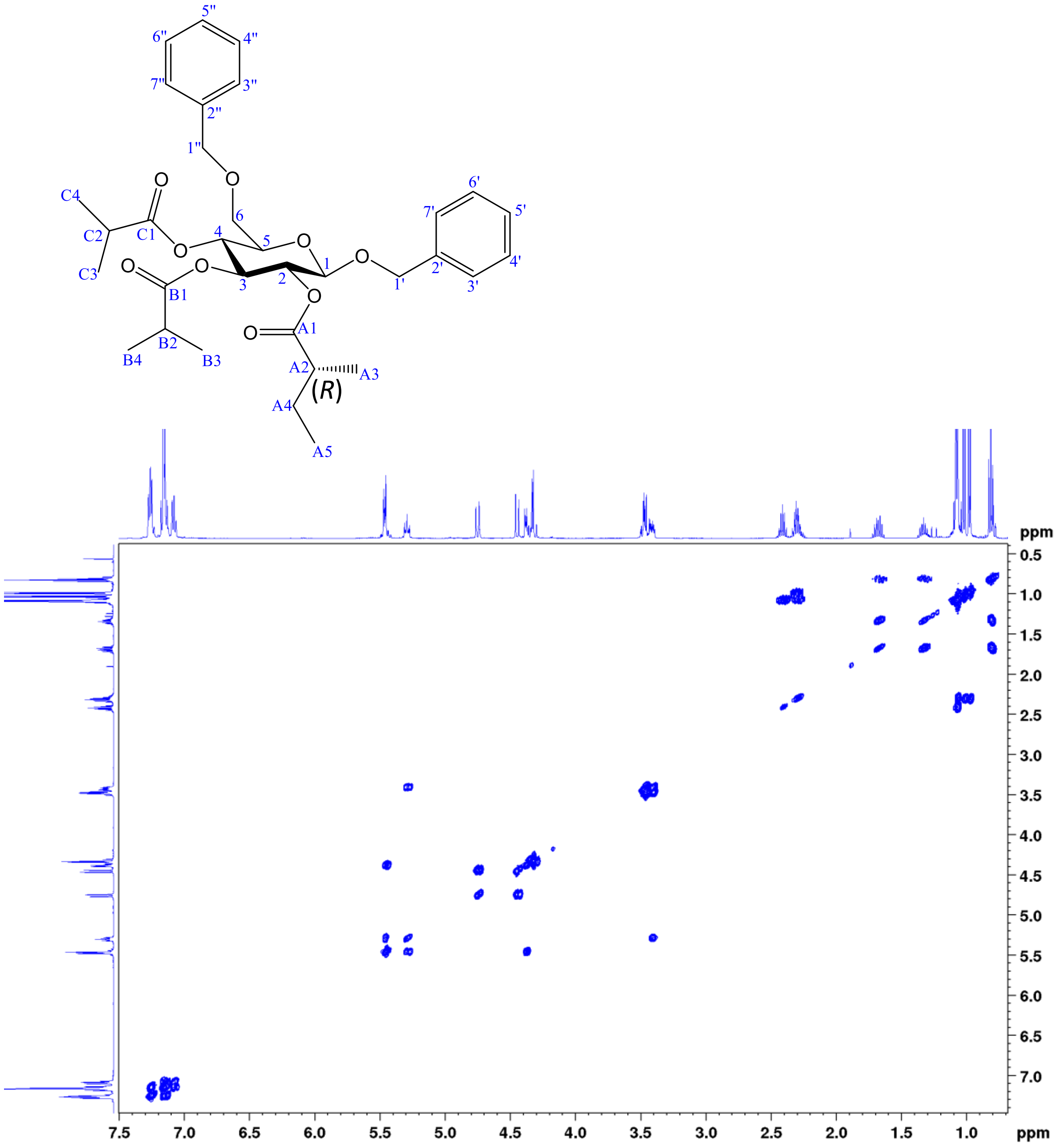

Figure S72: COSY spectrum of compound **12** (500 MHz, C_6D_6).

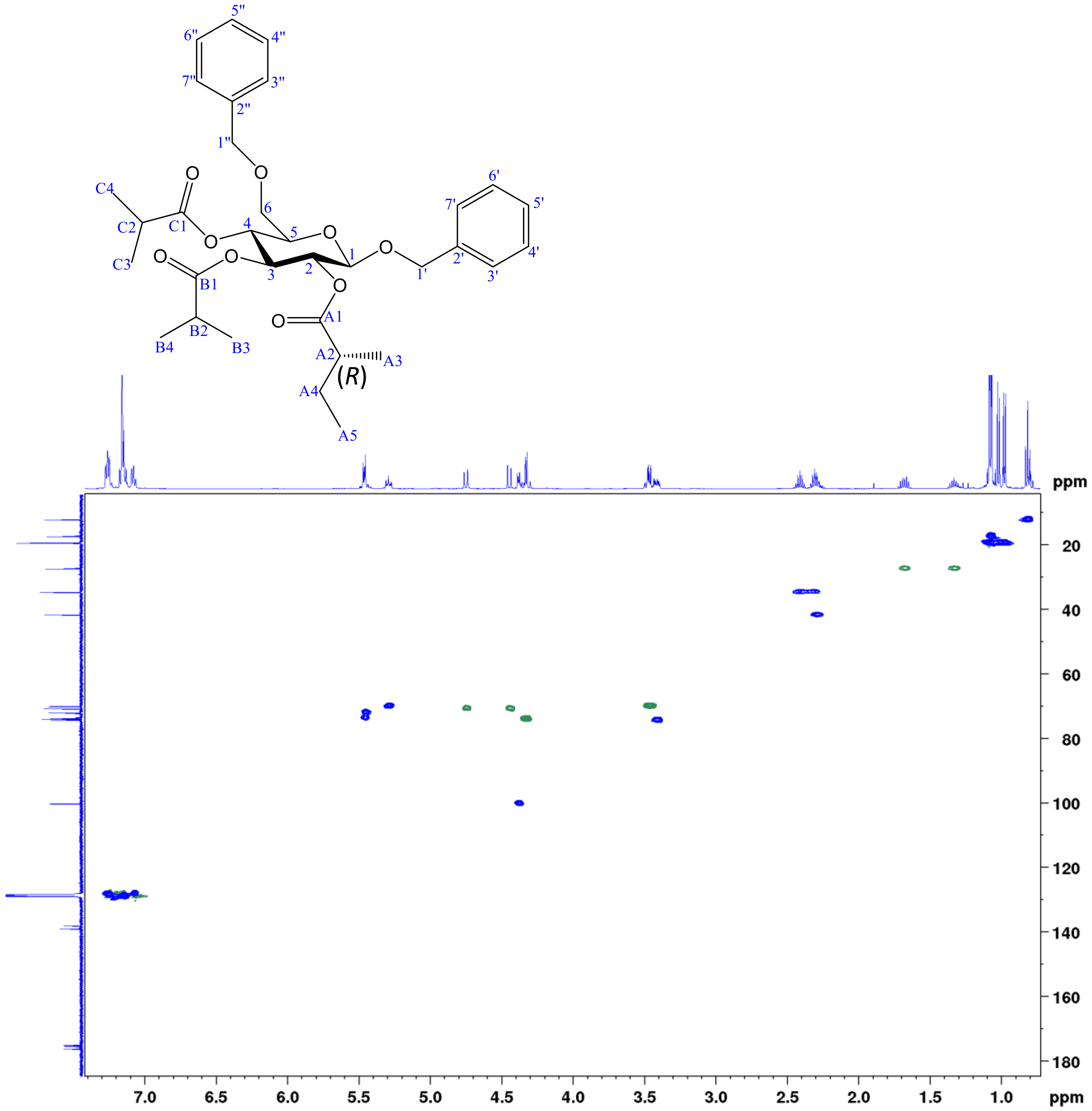

Figure S73: HSQC spectrum compound **12** (500 MHz, C_6D_6).

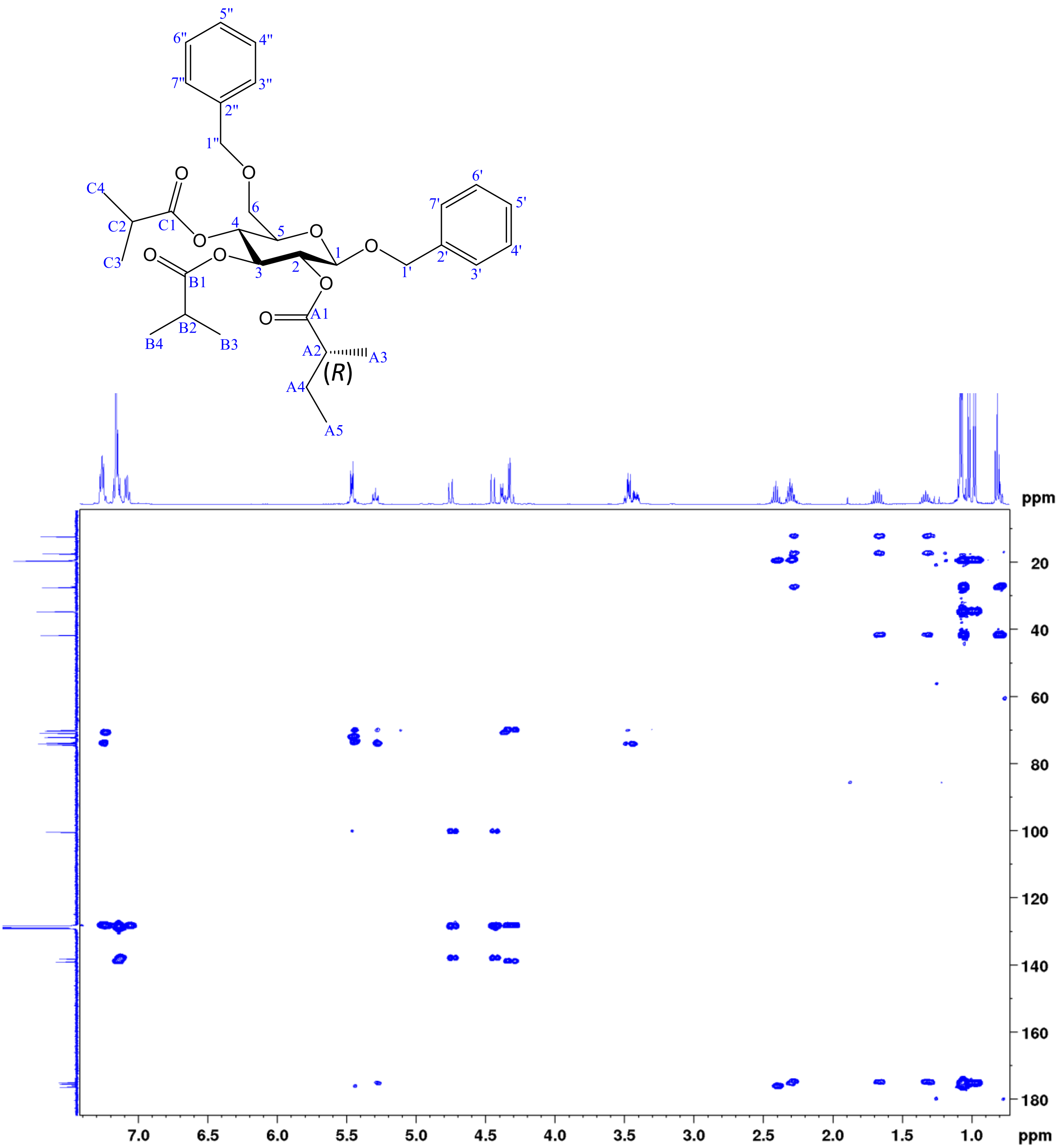

Figure S74: HMBC spectrum of compound **12** (500 MHz, C_6D_6).

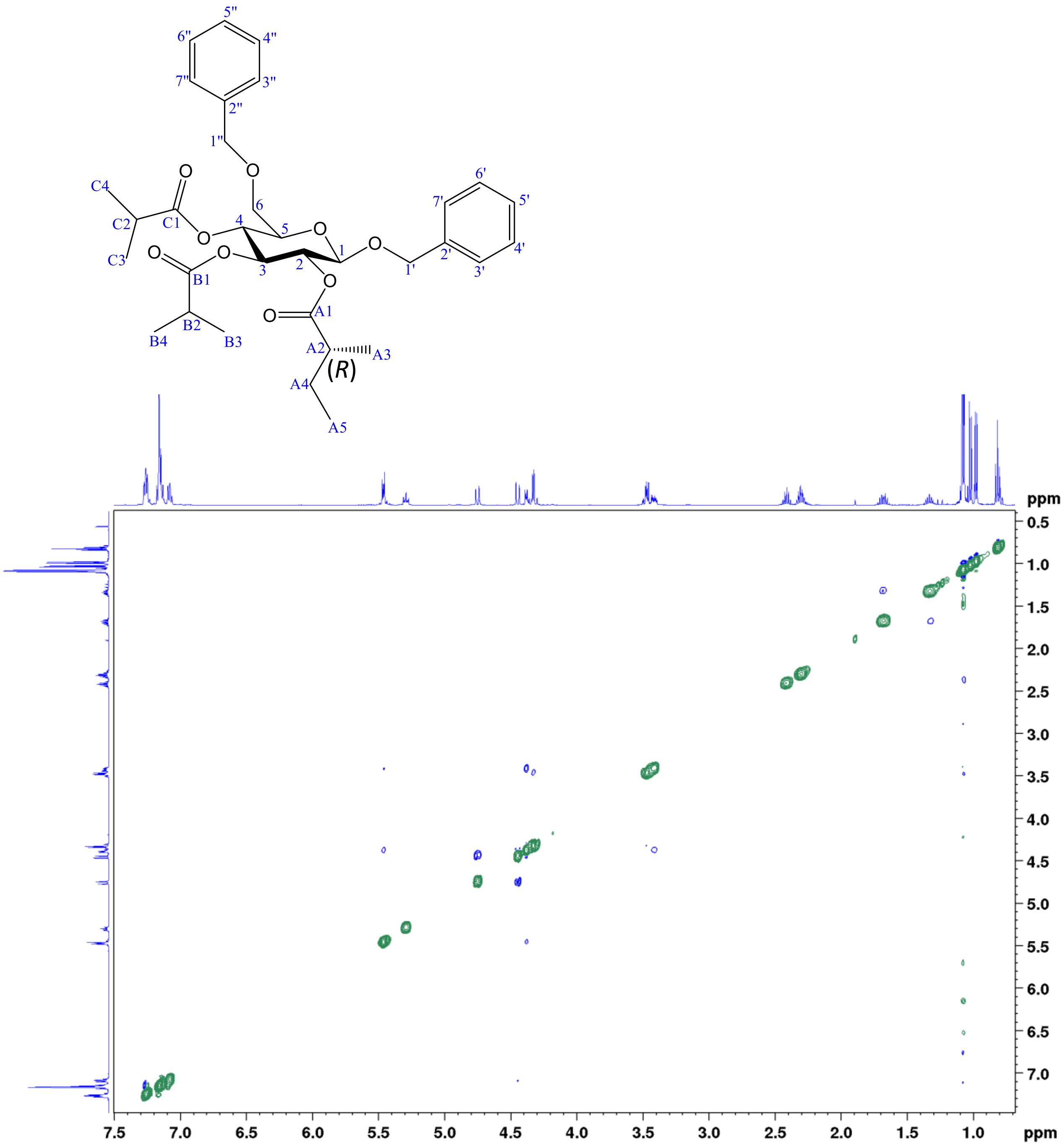

Figure S75: NOESY spectrum of compound **12** (500 MHz, C_6D_6).

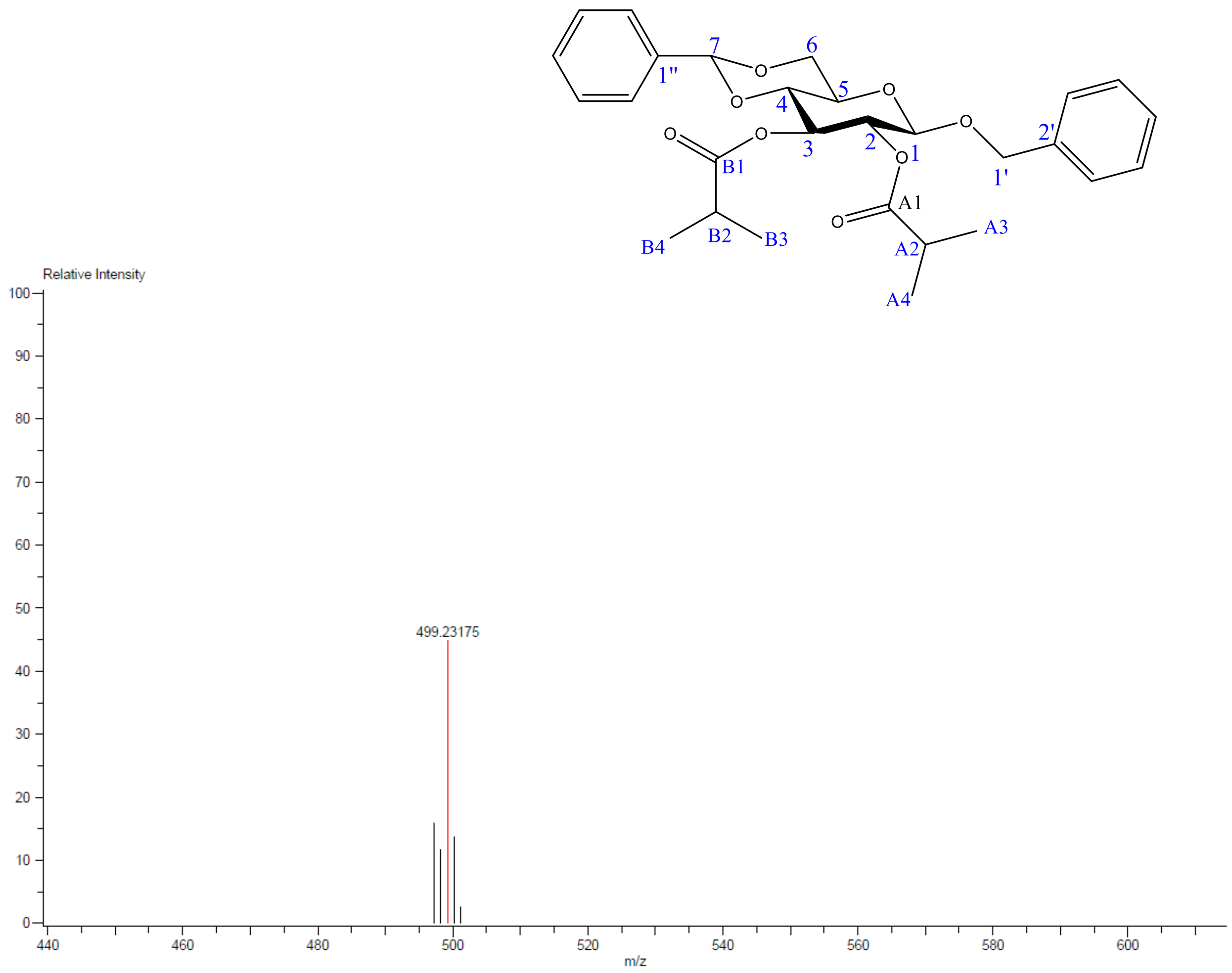

Figure S76. HRFD-MS spectrum of compound **13**.

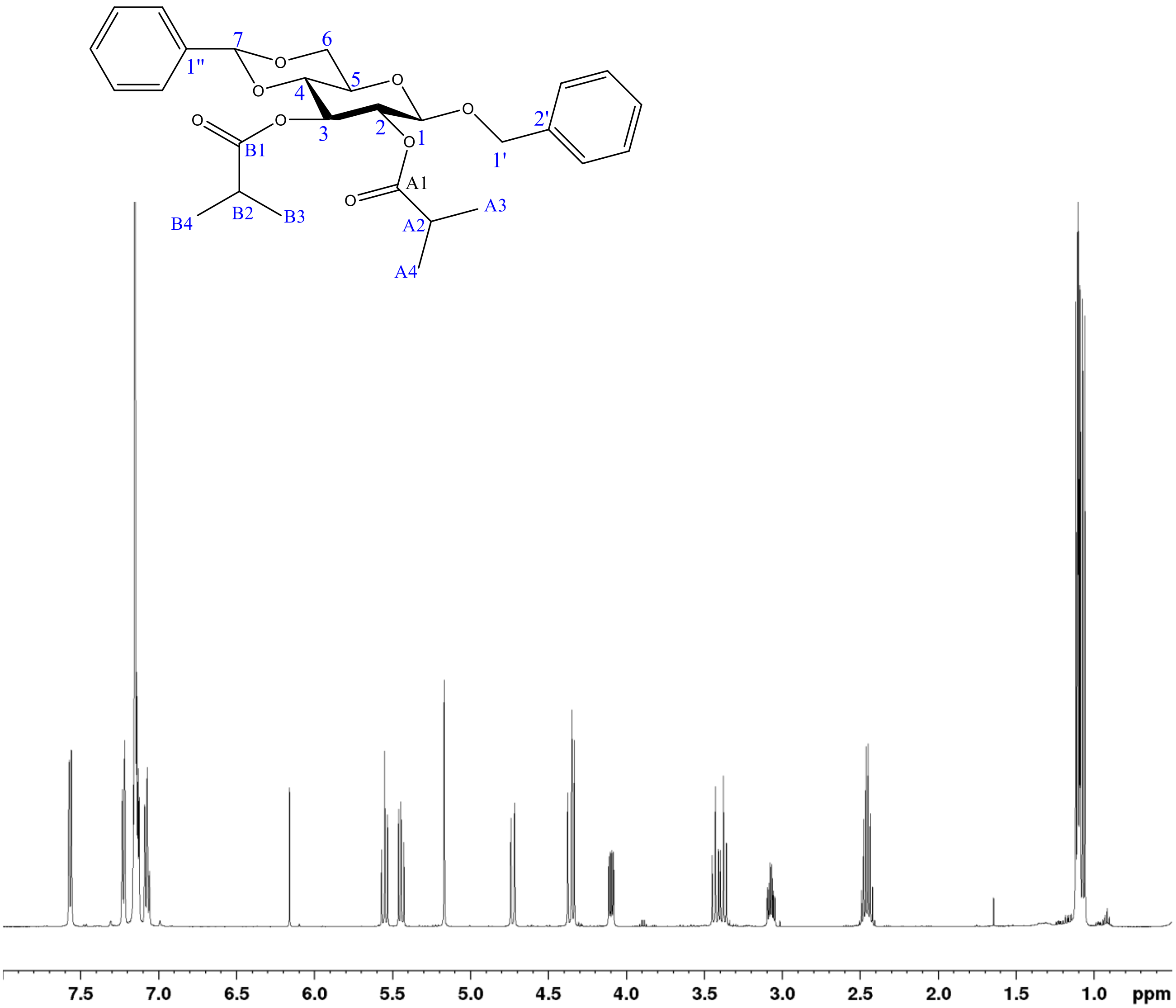

Figure S77: ^{1}H NMR spectrum of compound **13** (500 MHz, C_6D_6).

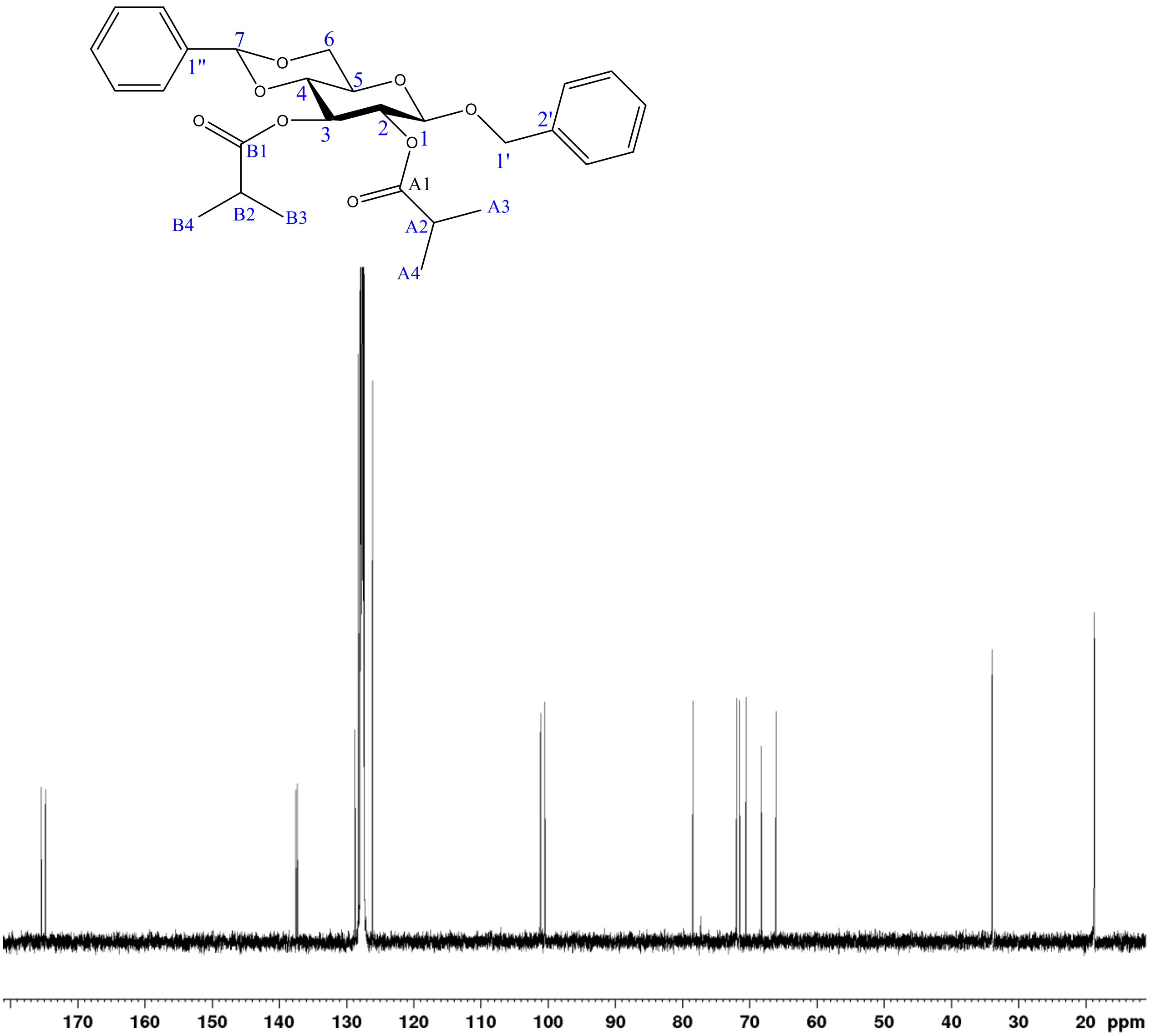

Figure S78: : ^{13}C NMR spectrum of compound **13 (**126 MHz, C_6D_6).

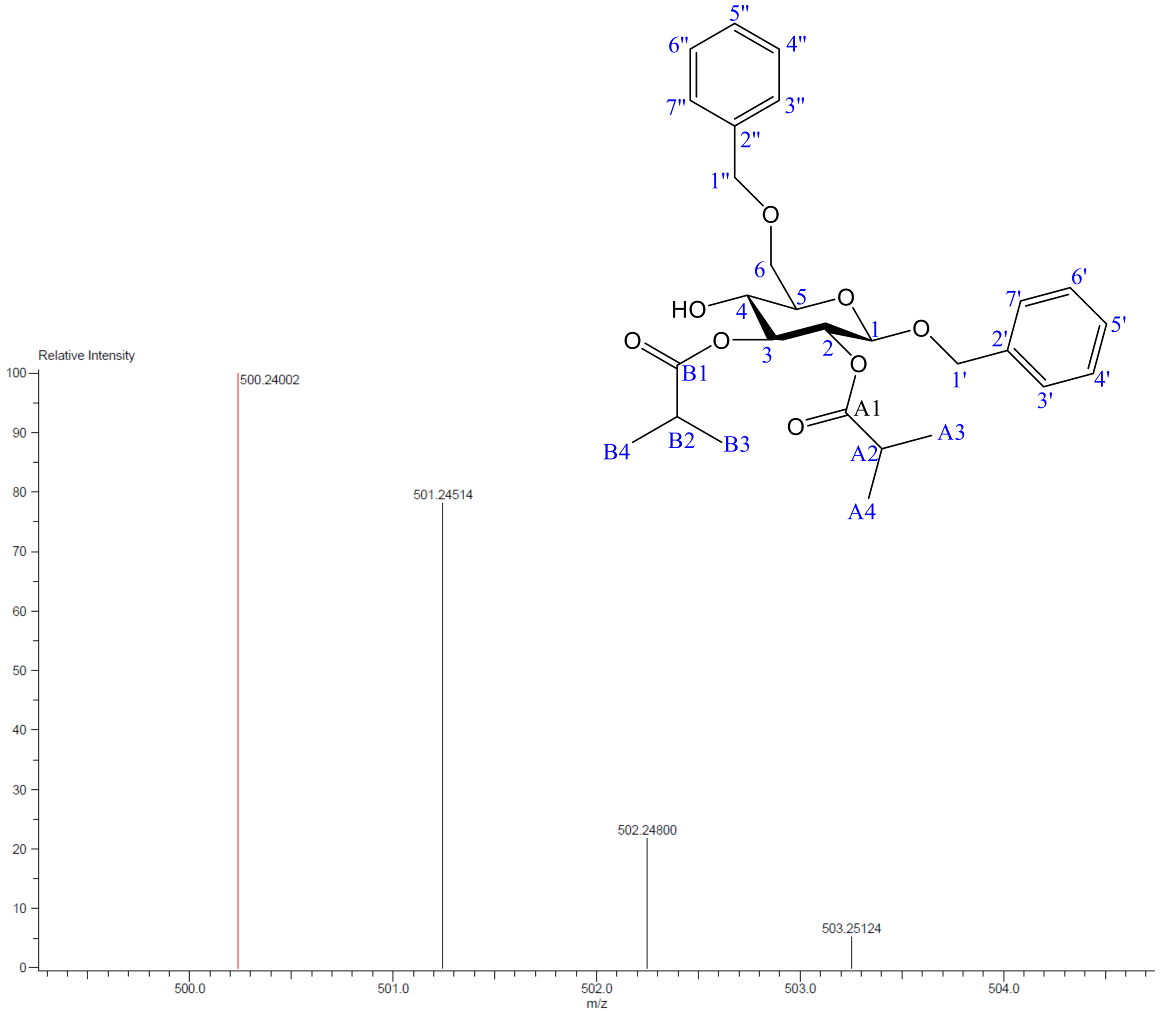

Figure S79. HRFD-MS spectrum of compound **16a** .

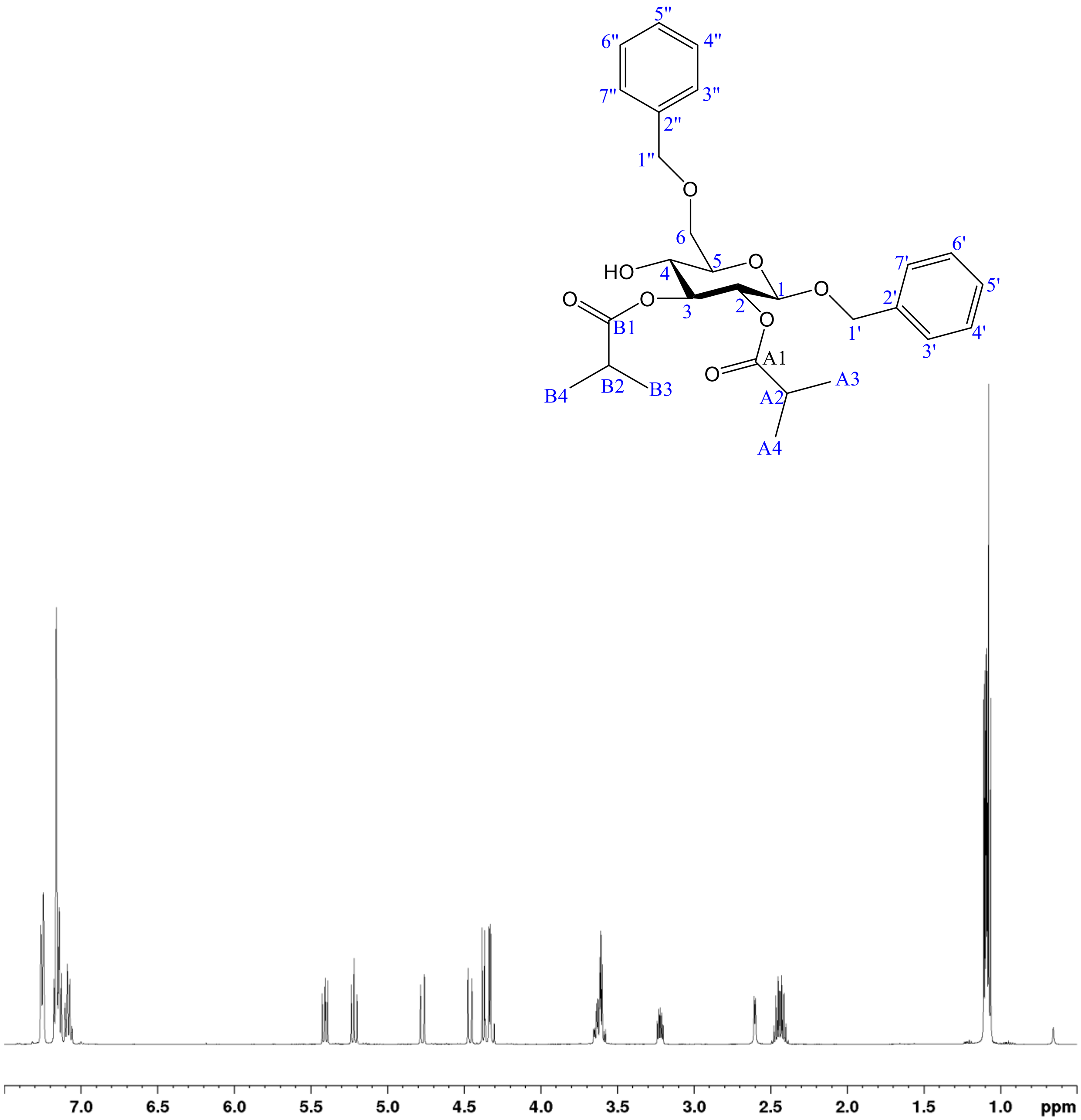

Figure S80: ^{1}H NMR spectrum of compound **16a** (500 MHz, C_6D_6).

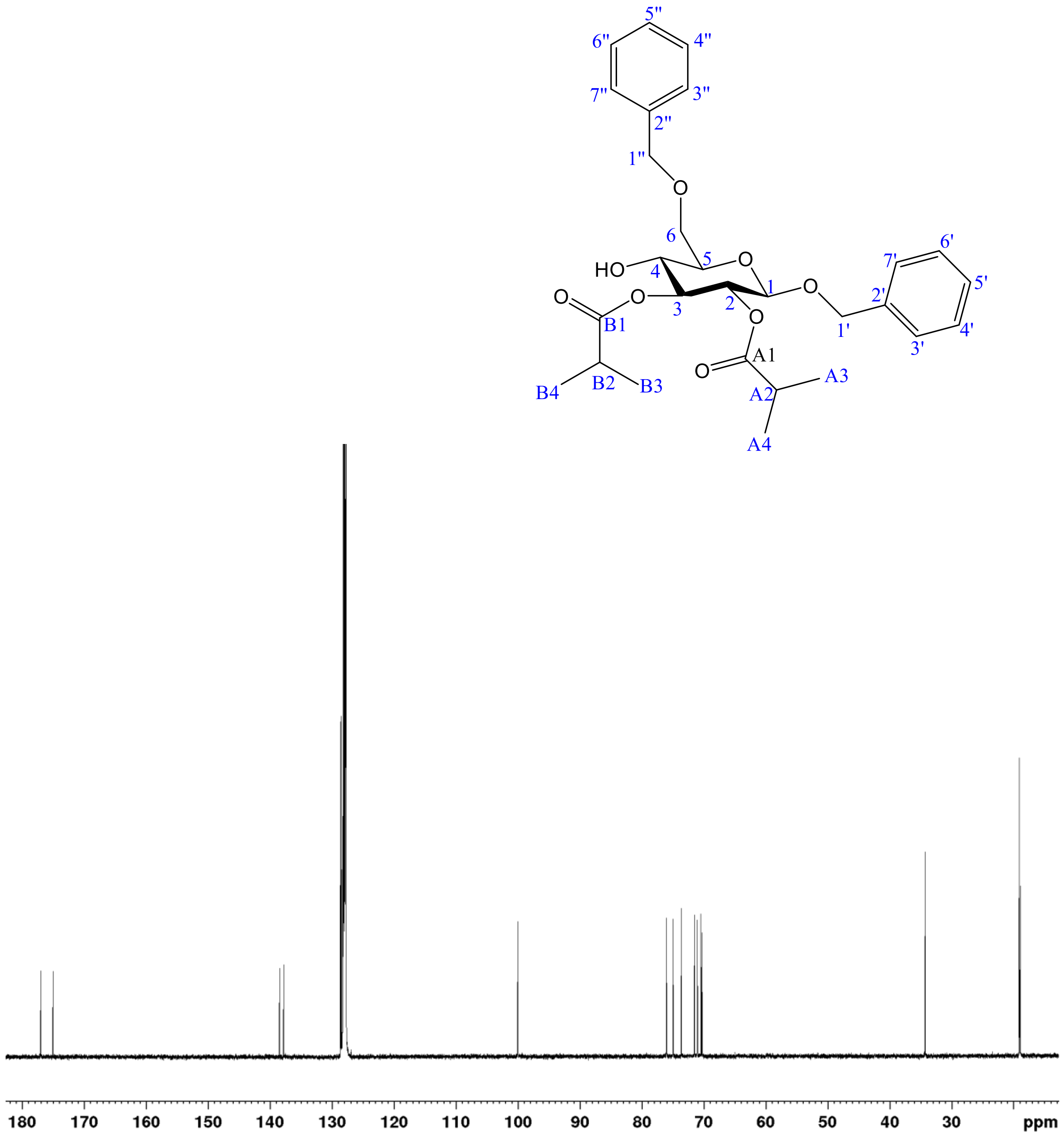

Figure S81: ^{13}C NMR spectrum of compound **16a** (126 MHz, C_6D_6).

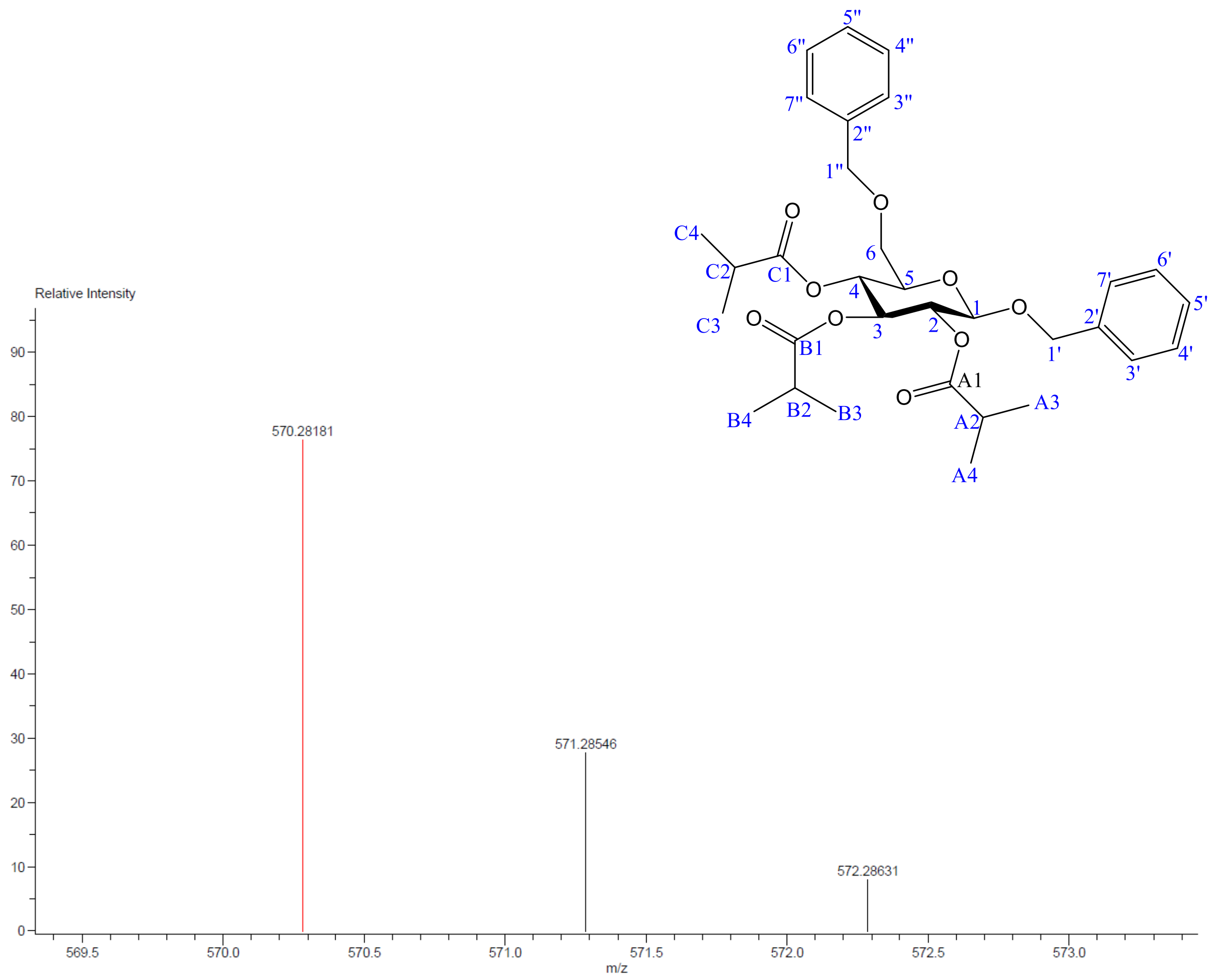

Figure S82. HRFD-MS spectrum of compound **17a** .

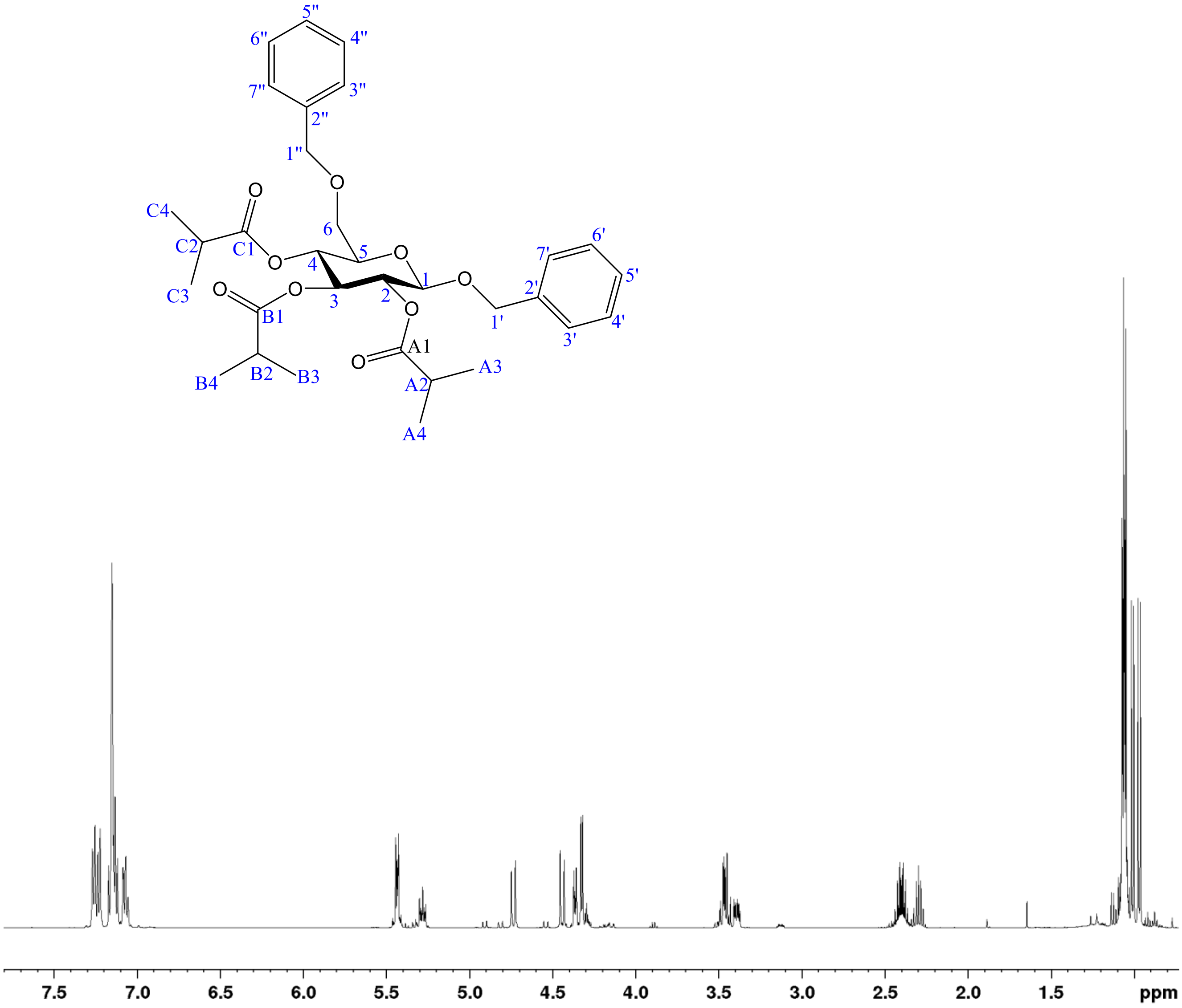

Figure S83: ^{1}H NMR spectrum of compound **17a** (500 MHz, C_6D_6).

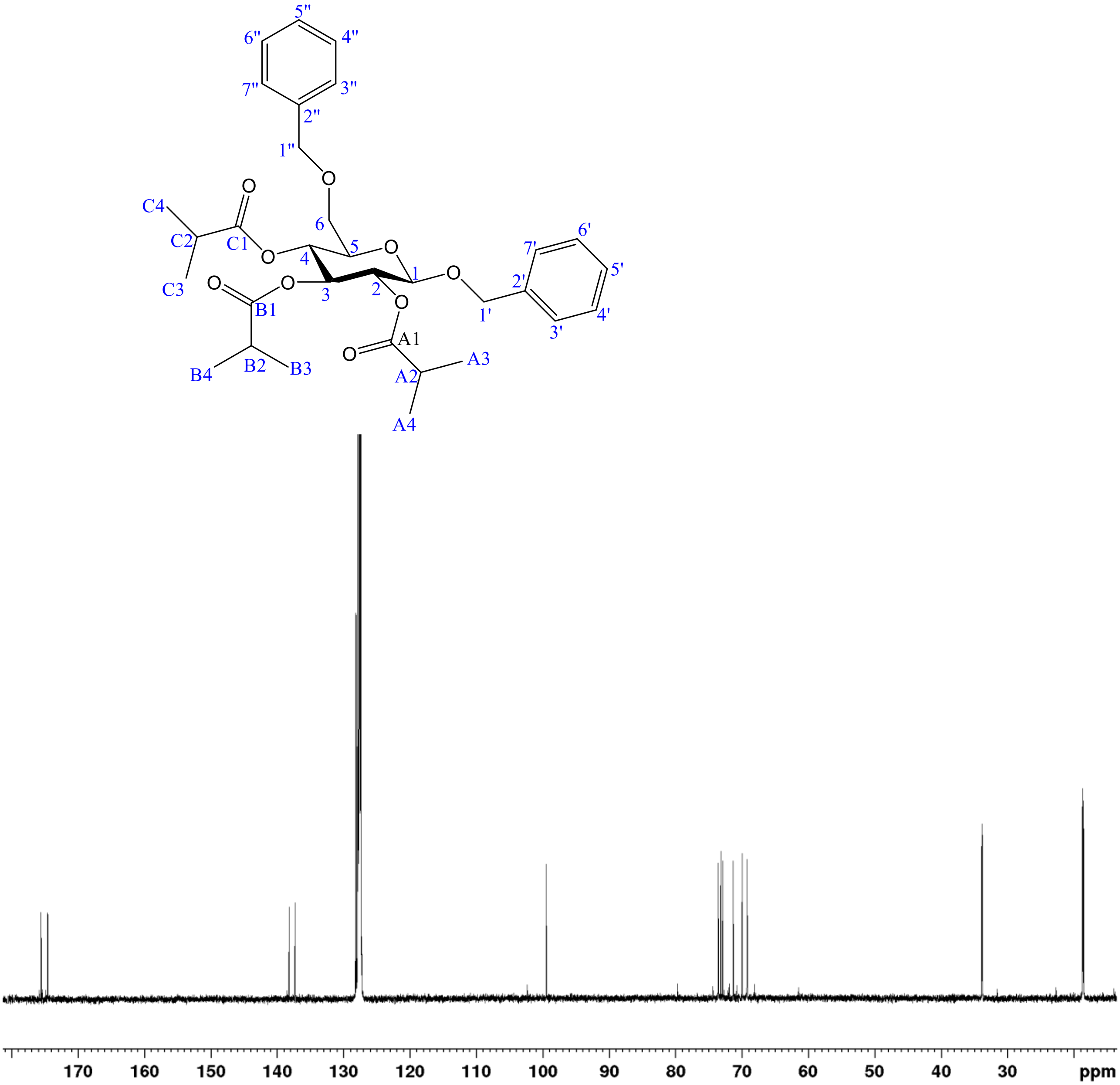

Figure S84: ^{13}C NMR spectrum of compound **17a** (126 MHz, C_6D_6).

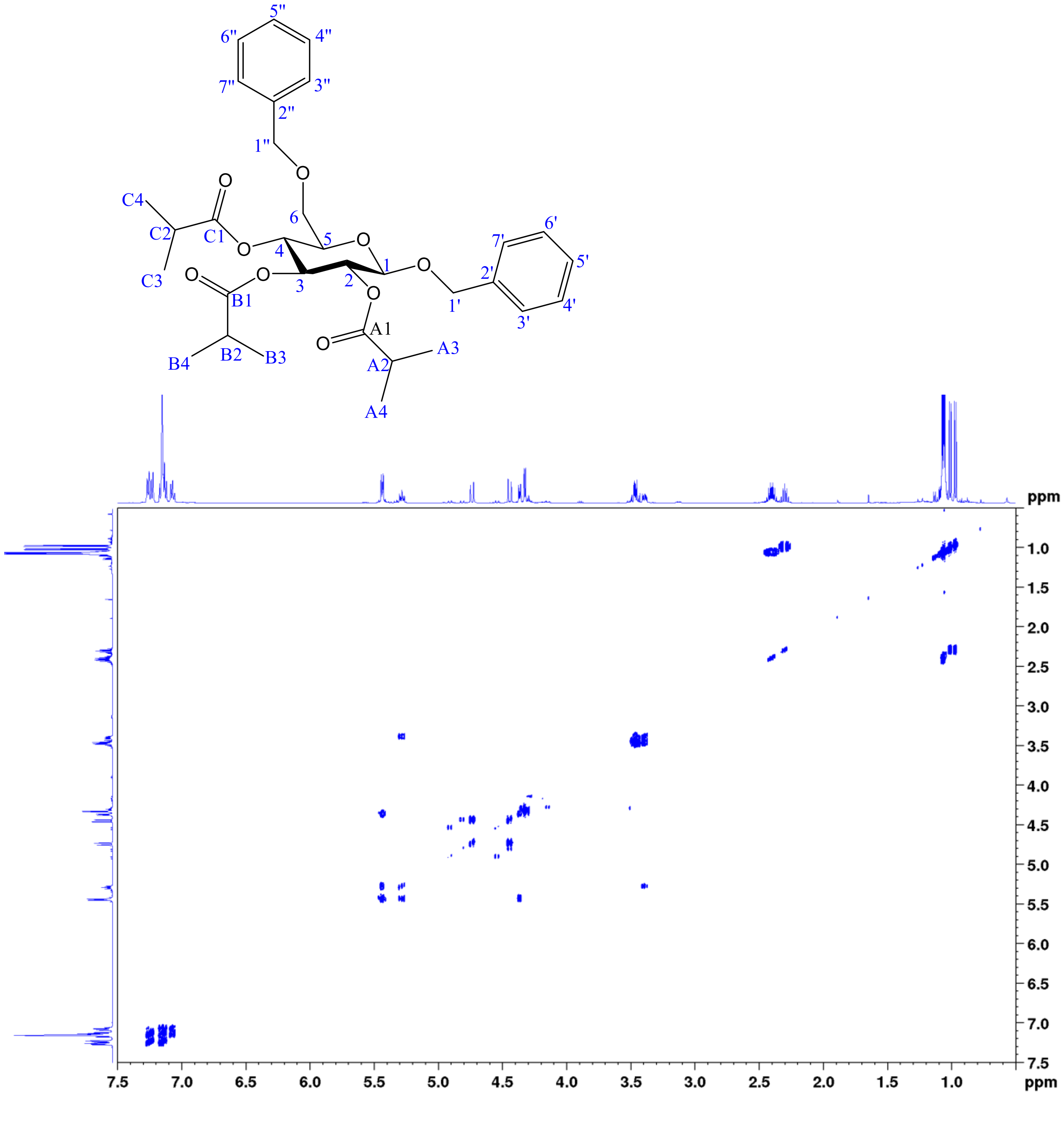

Figure S85: COSY spectrum of compound **17a** (500 MHz, C_6D_6).

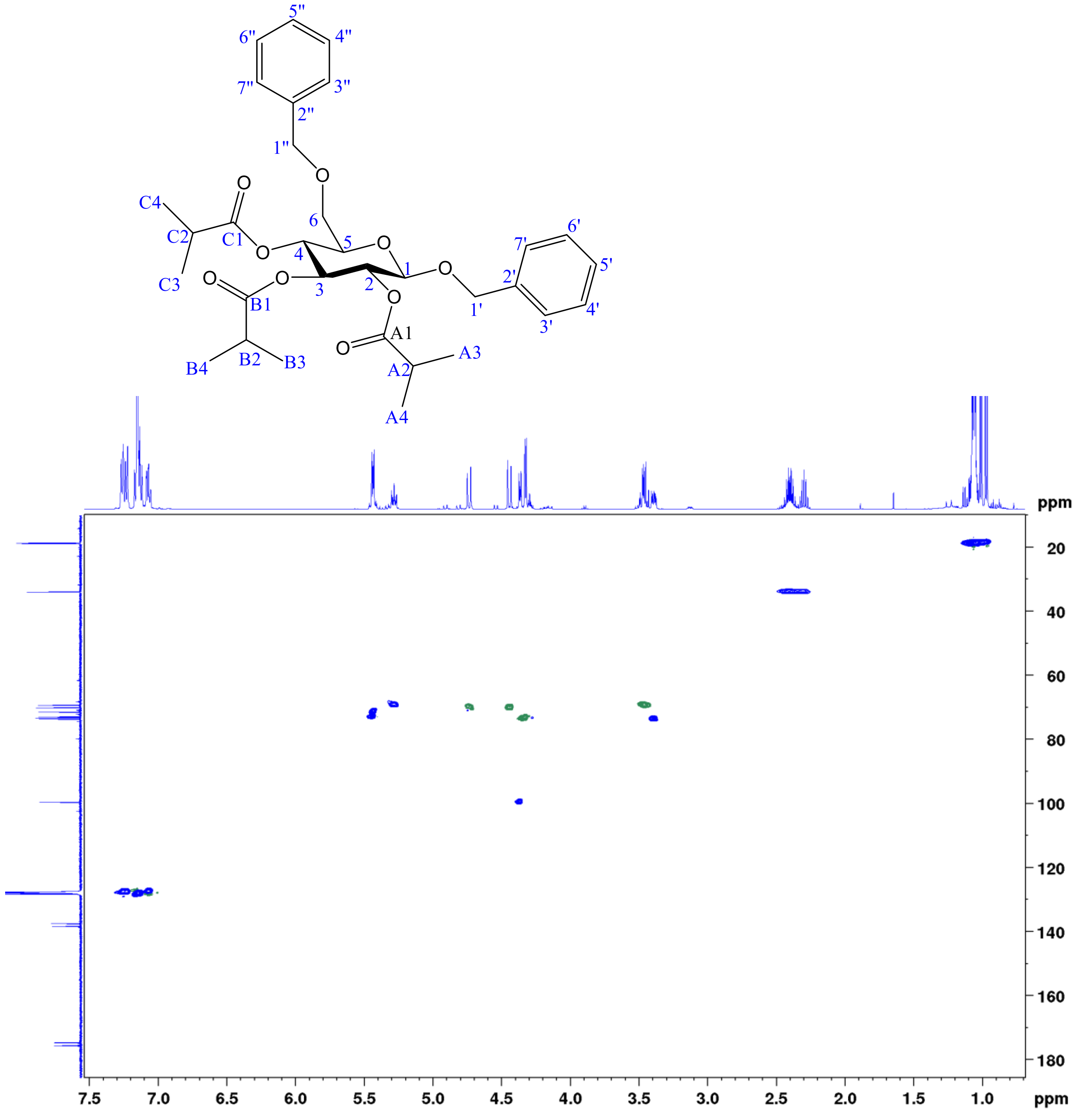

Figure S86: HSQC spectrum of compound **17a** (500 MHz, C_6D_6).

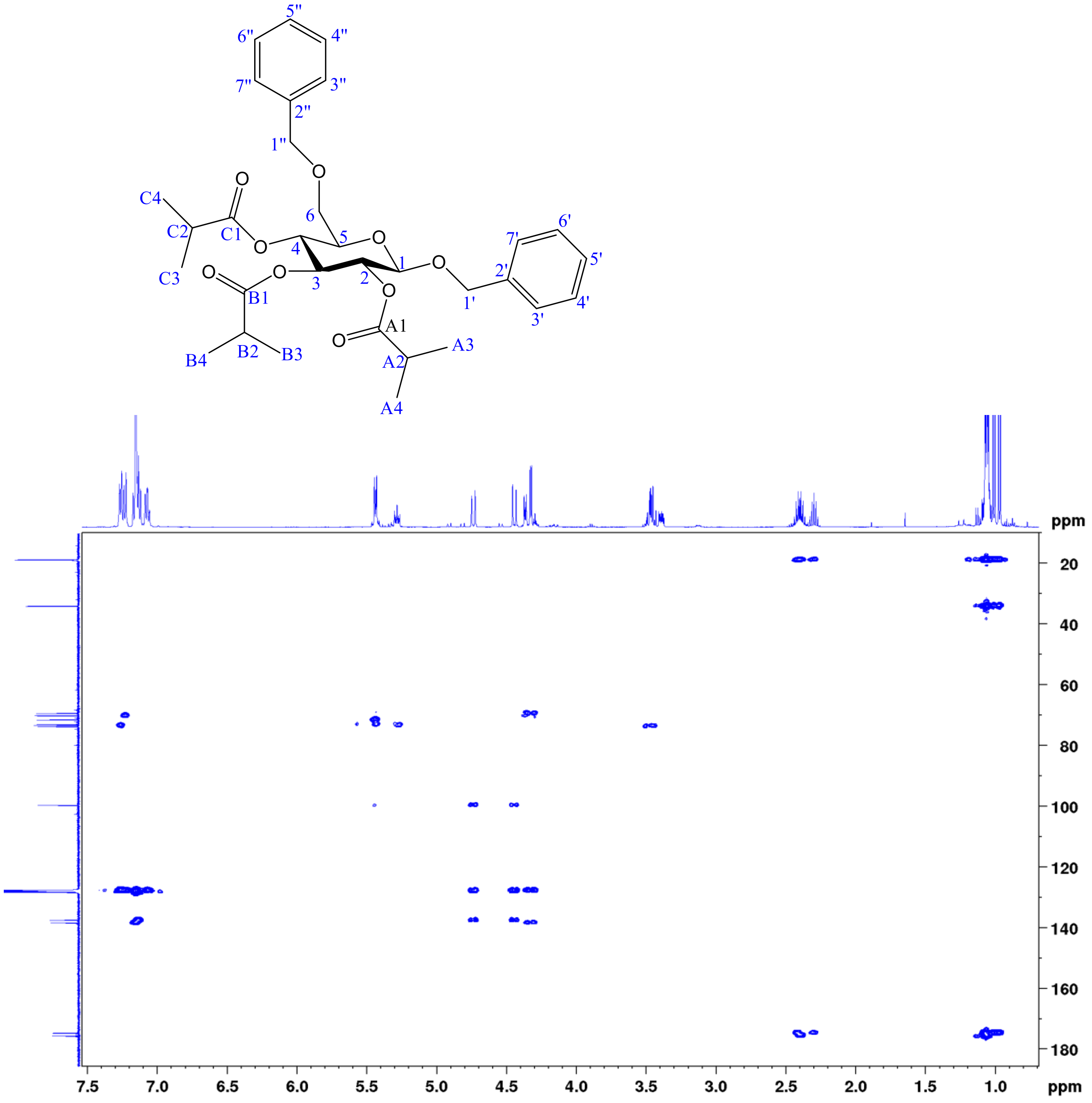

Figure S87: HMBC spectrum of compound **17a** (500 MHz, C_6D_6).

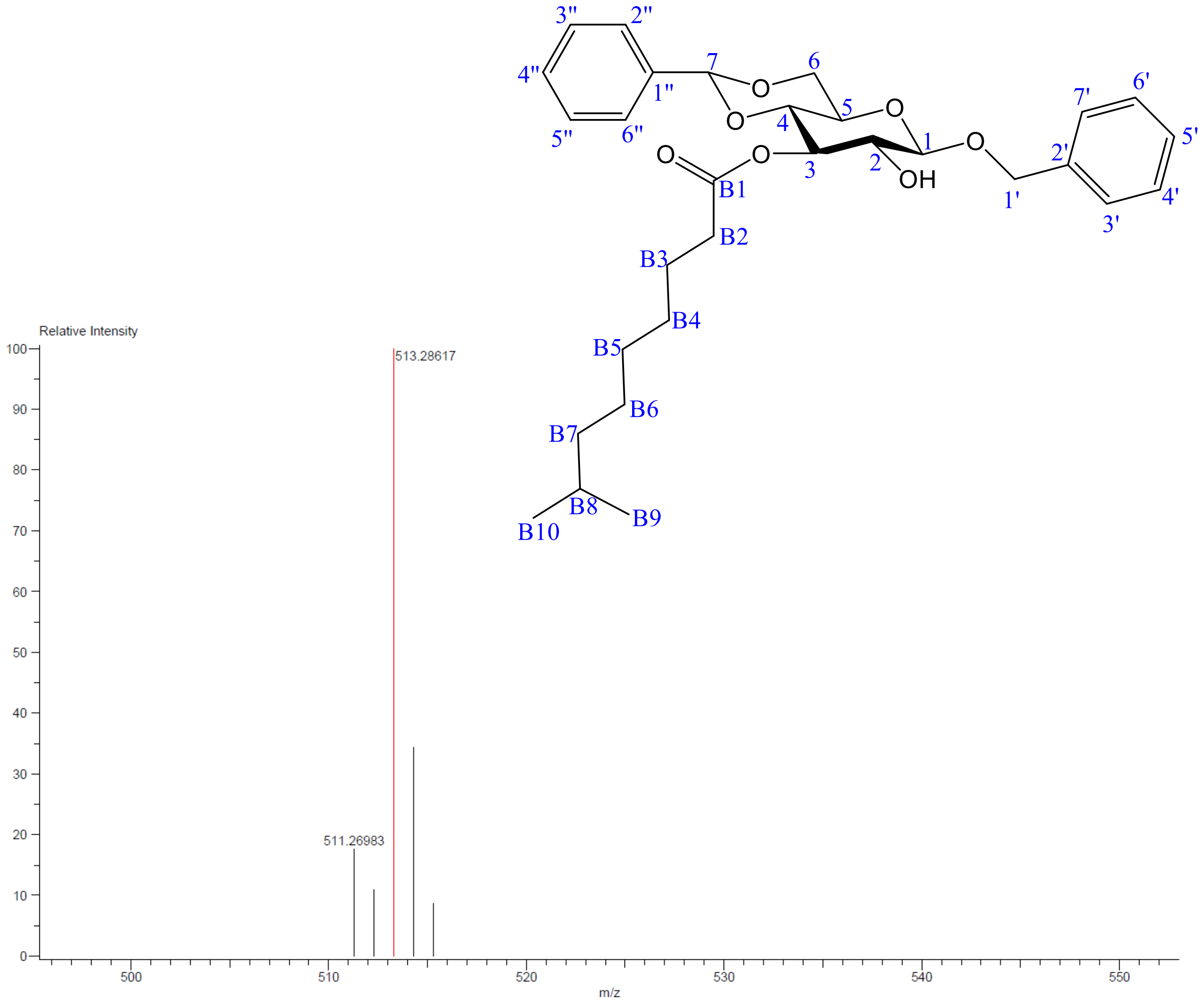

Figure S88. HRFD-MS spectrum of compound **14** .

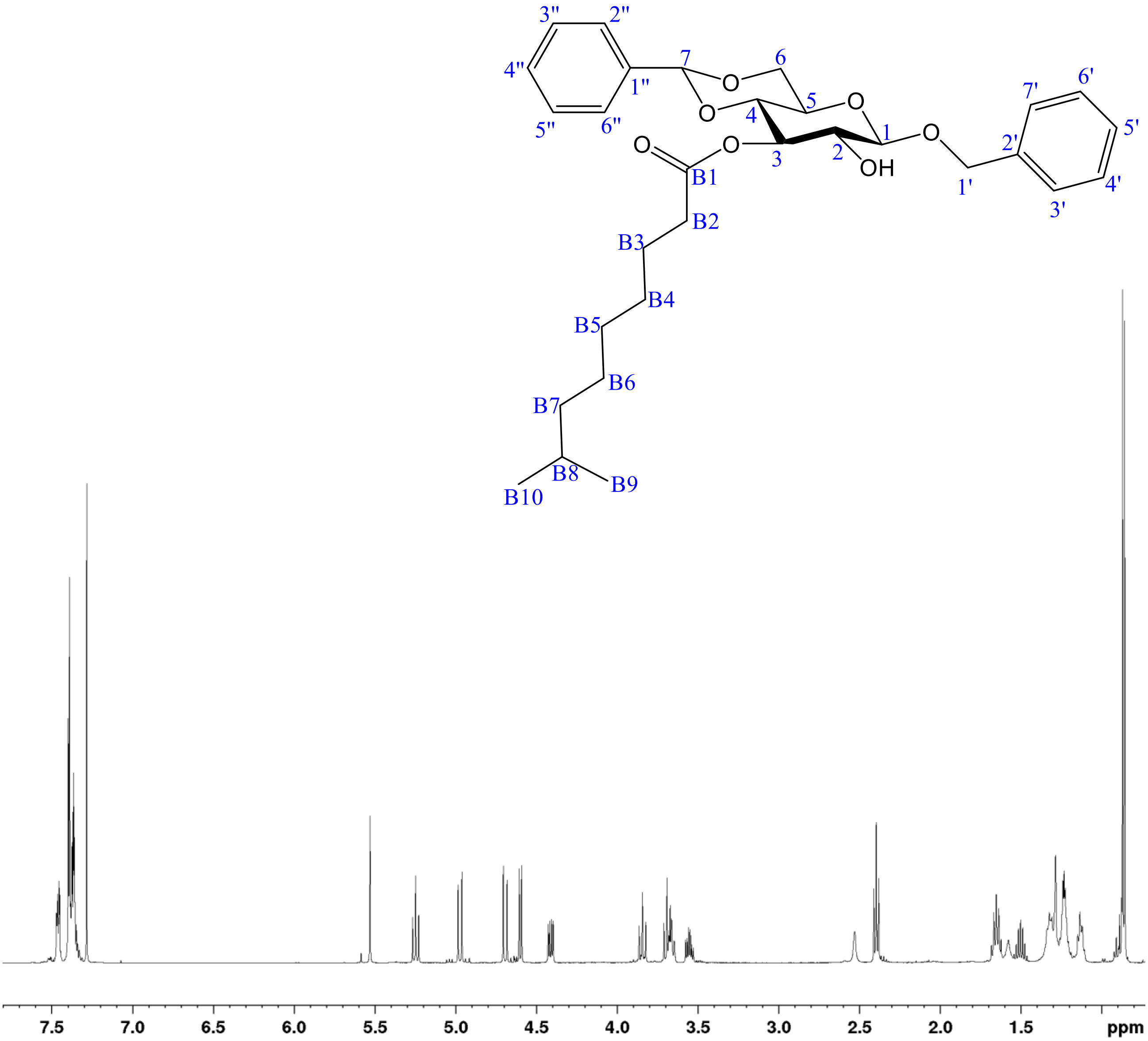

Figure S89: ^{1}H NMR spectrum of compound **14** (500 MHz, $CDCl_3$).

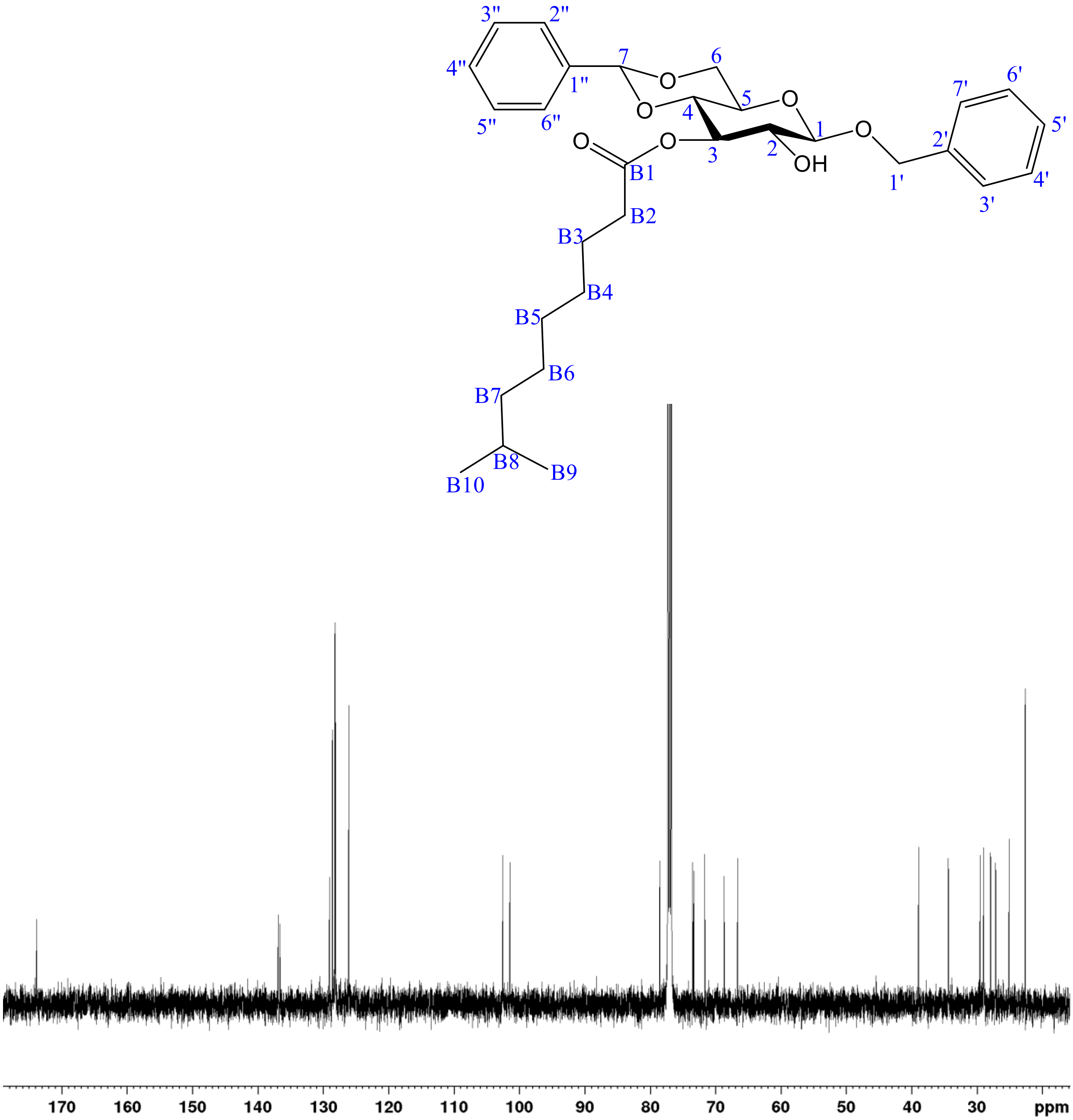

Figure S90: ^{13}C NMR spectrum of compound **14** (126 MHz, $CDCl_3$).

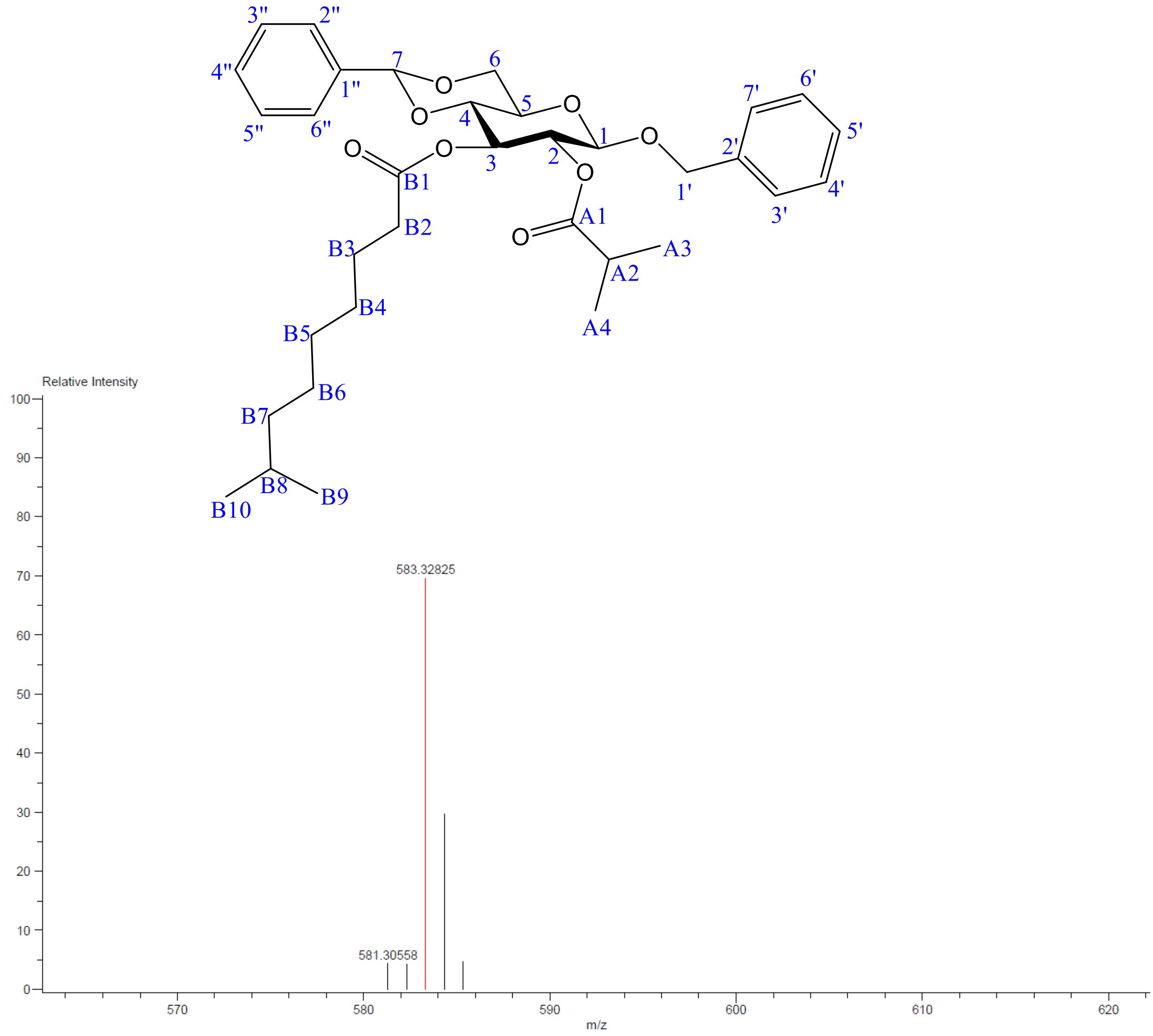

Figure S91. HRFD-MS spectrum of compound **15**.

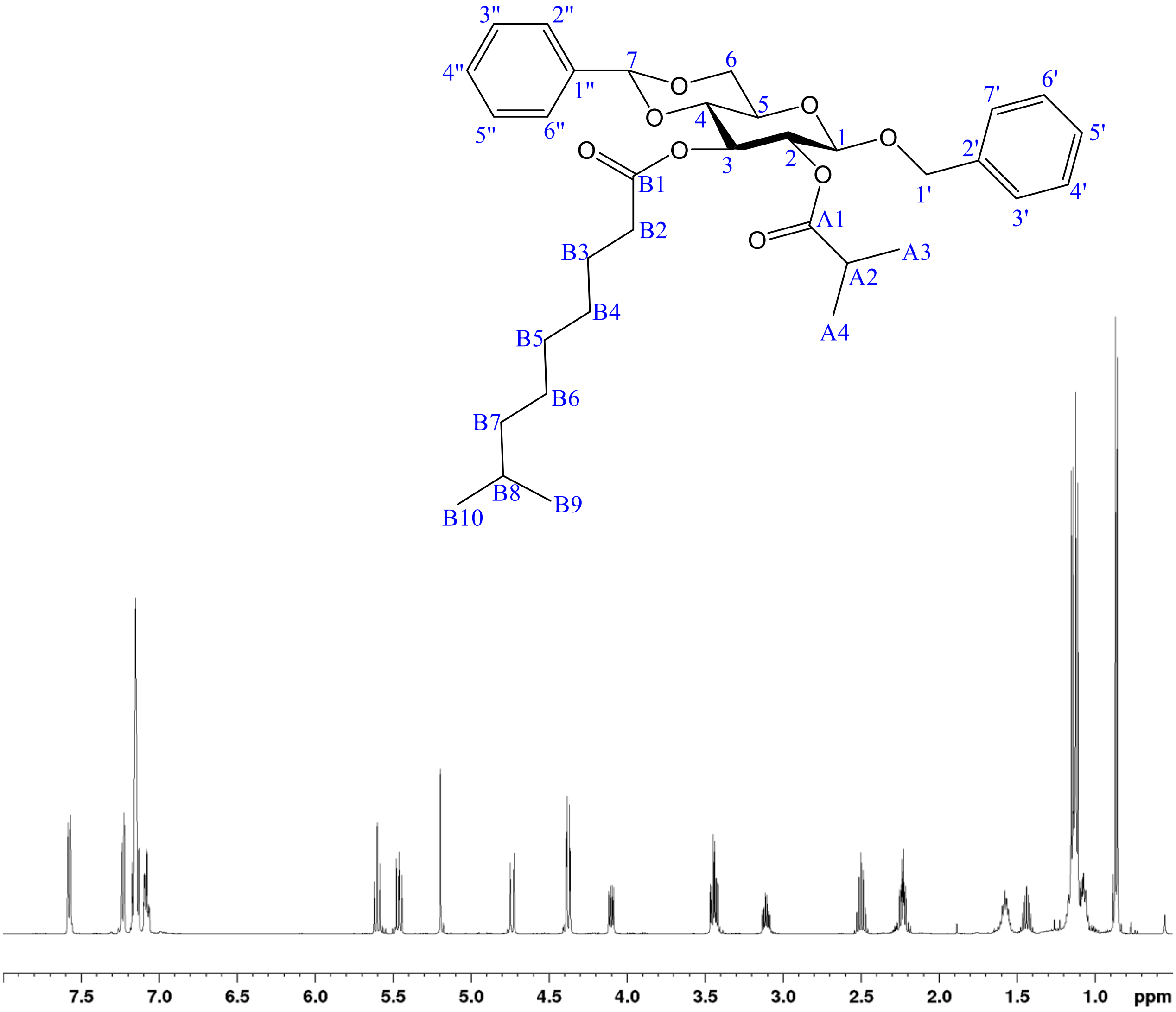

Figure S92: ^{1}H NMR spectrum of compound **15** (500 MHz, C_6D_6).

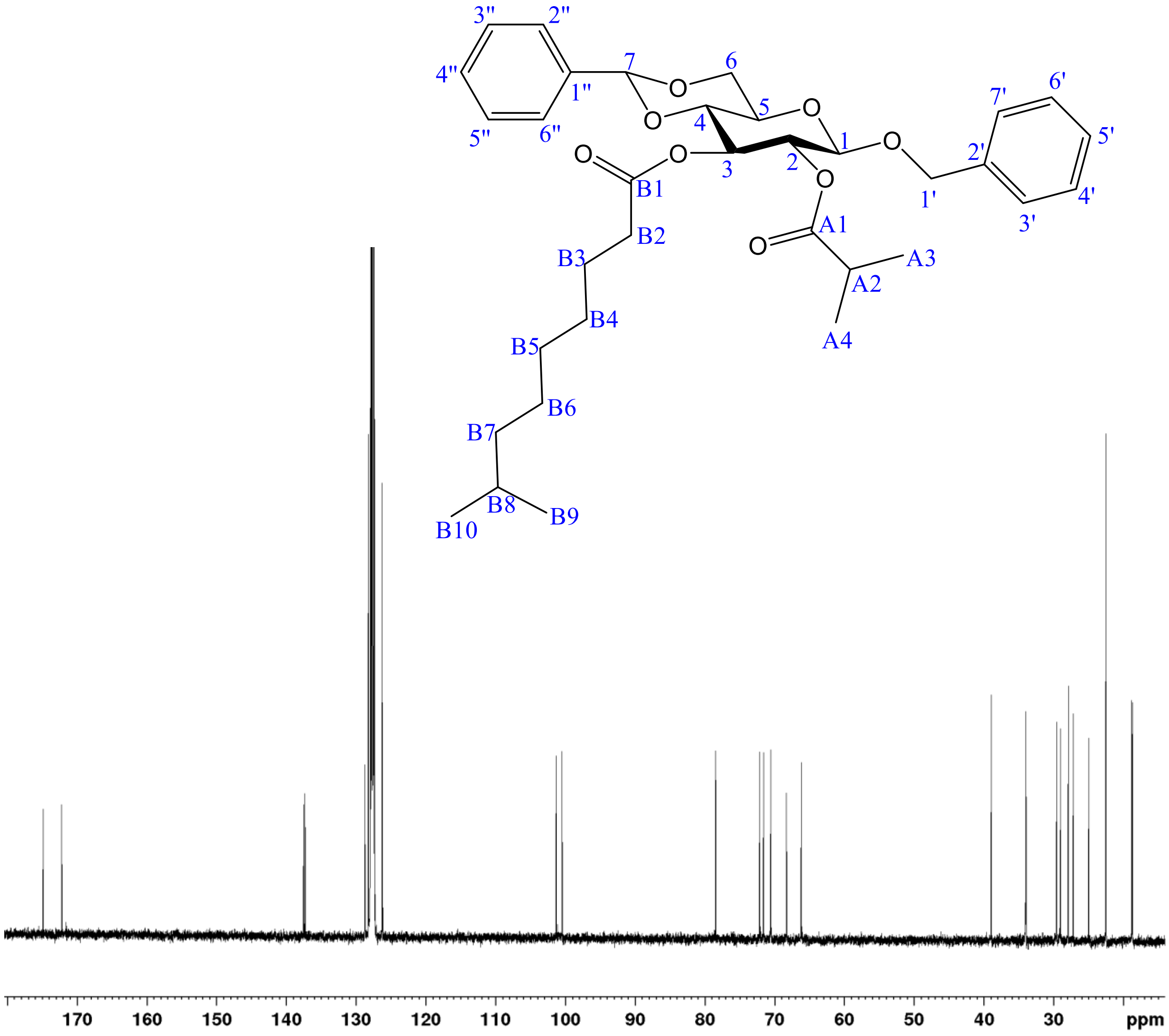

Figure S93: ^{13}C NMR spectrum of compound **15** (126 MHz, C_6D_6).

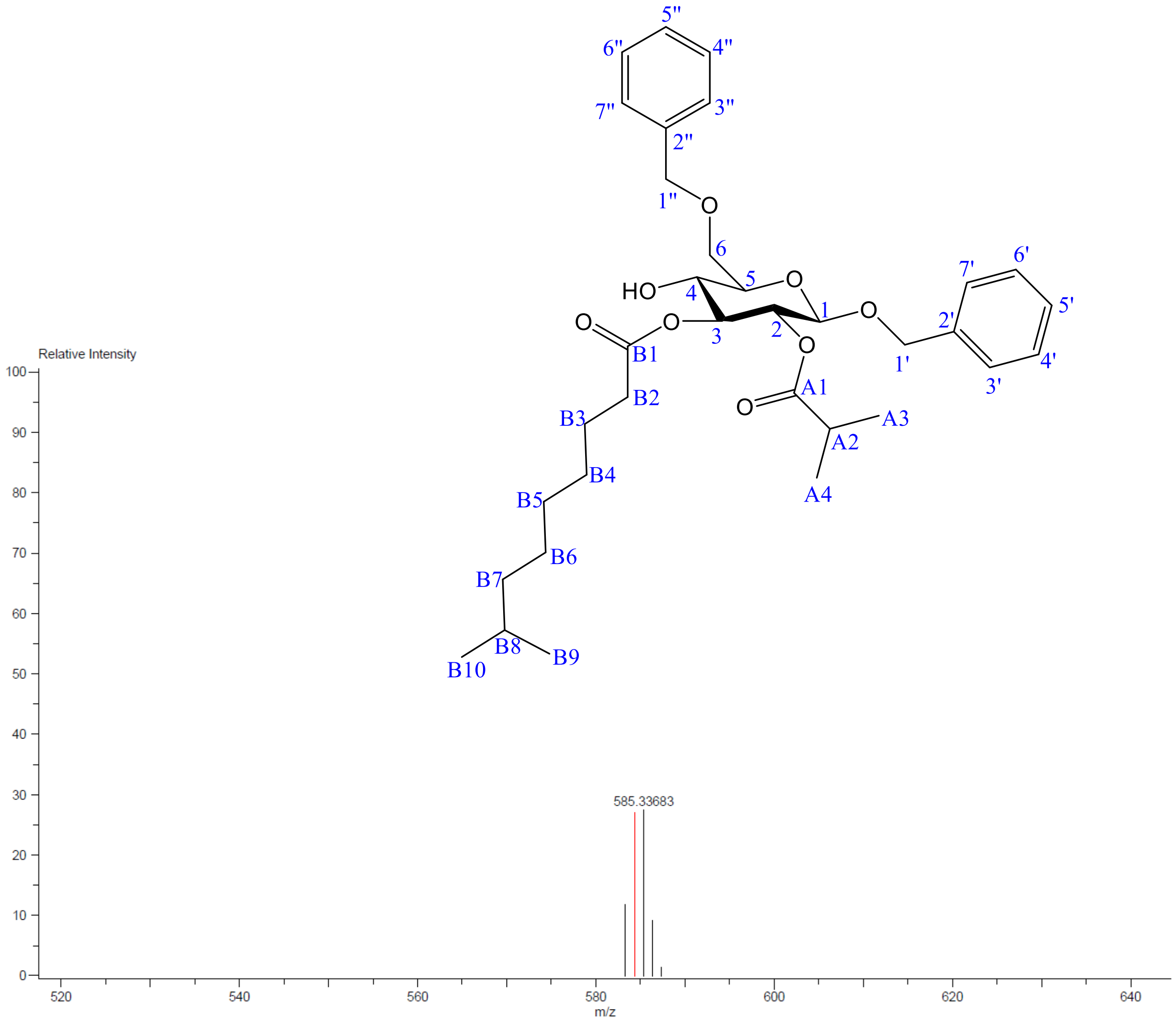

Figure S94. HRFD-MS spectrum of compound **16b** .

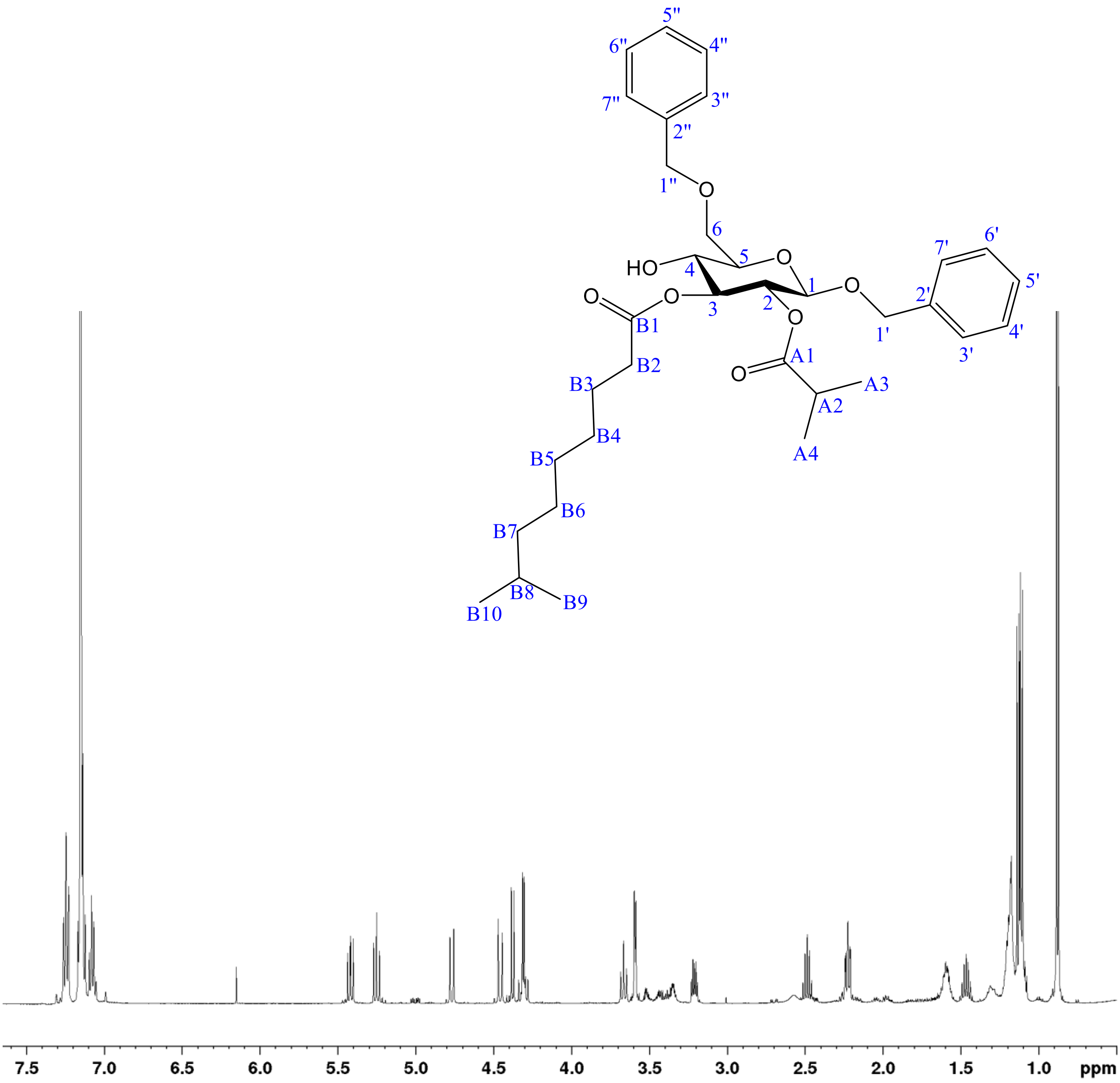

Figure S95: ^{1}H NMR spectrum of compound **16b** (500 MHz, C_6D_6).

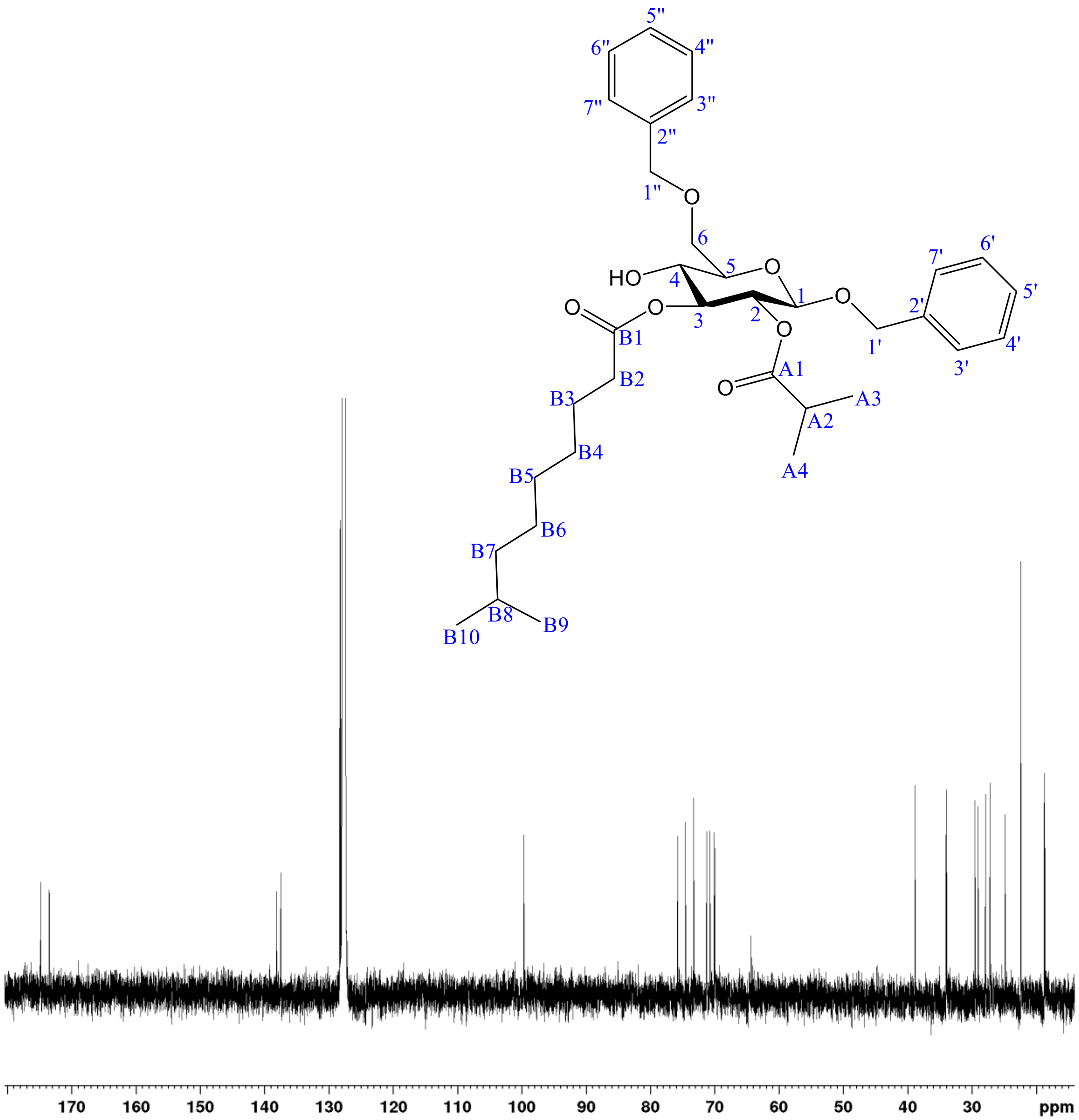

Figure S96: ^{13}C NMR spectrum of compound **16b** (126 MHz, C_6D_6).

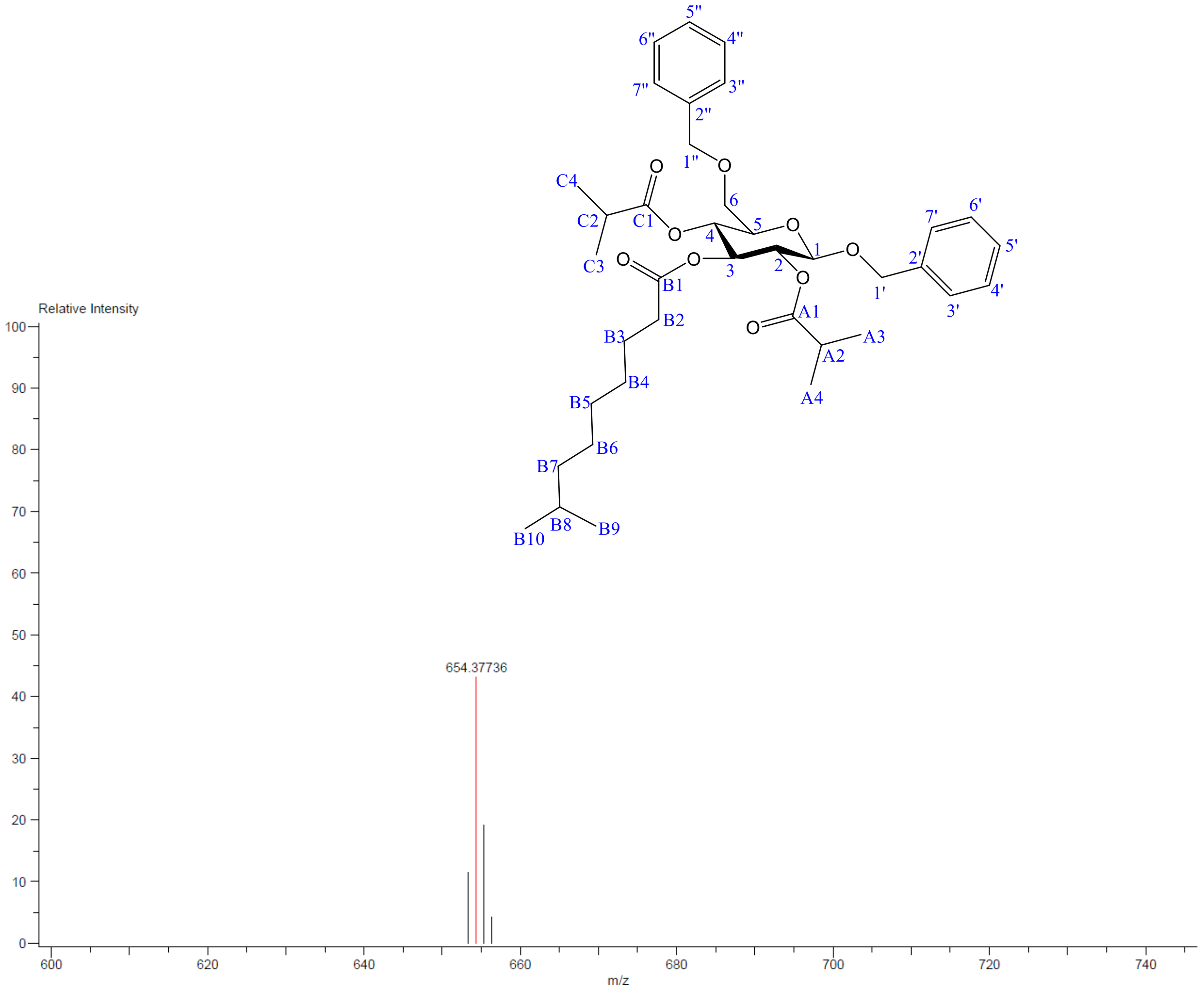

Figure S97. HRFD-MS spectrum of compound **17b** .

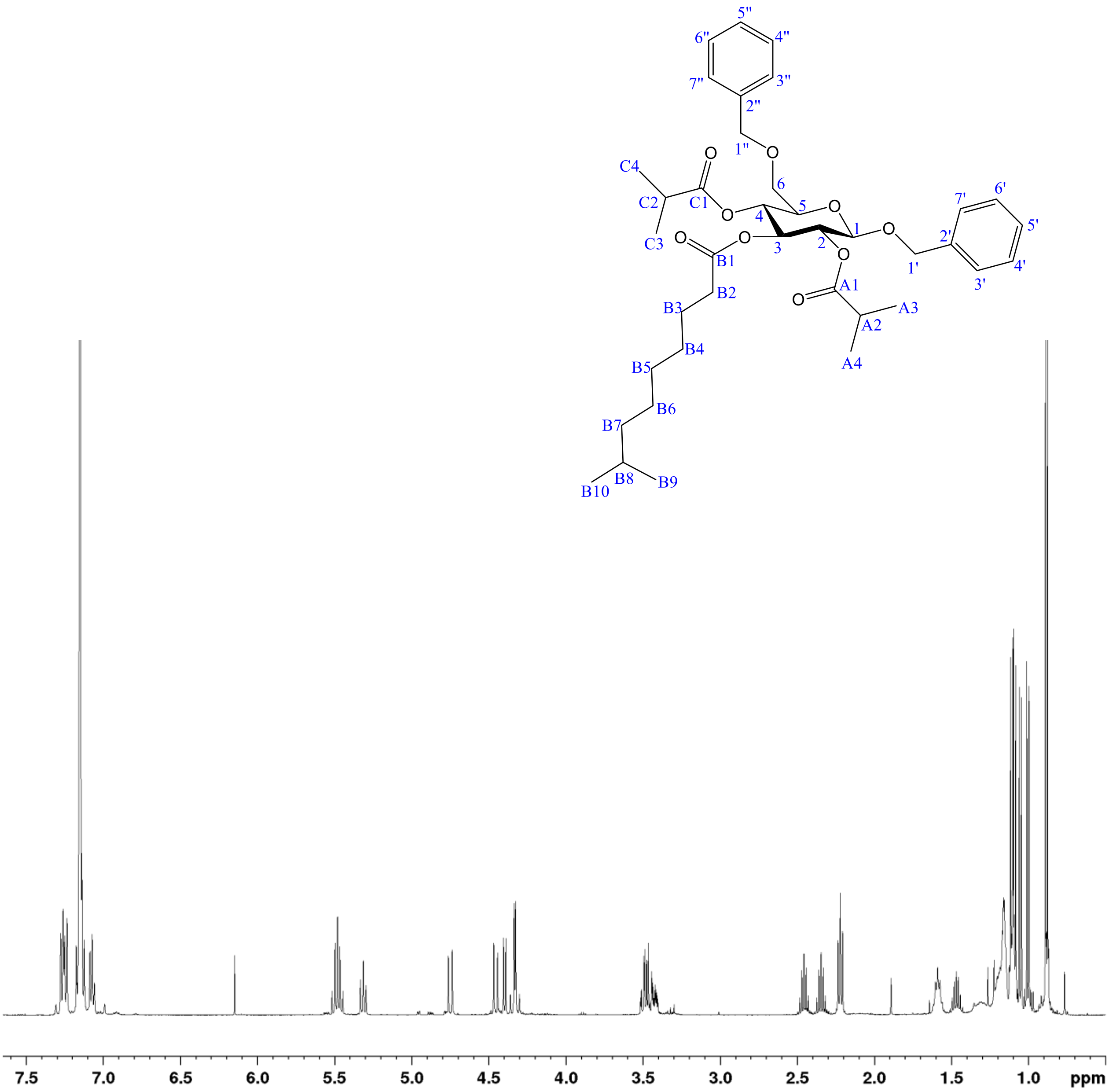

Figure S98: ^{1}H NMR spectrum of compound **17b** (500 MHz, C_6D_6).

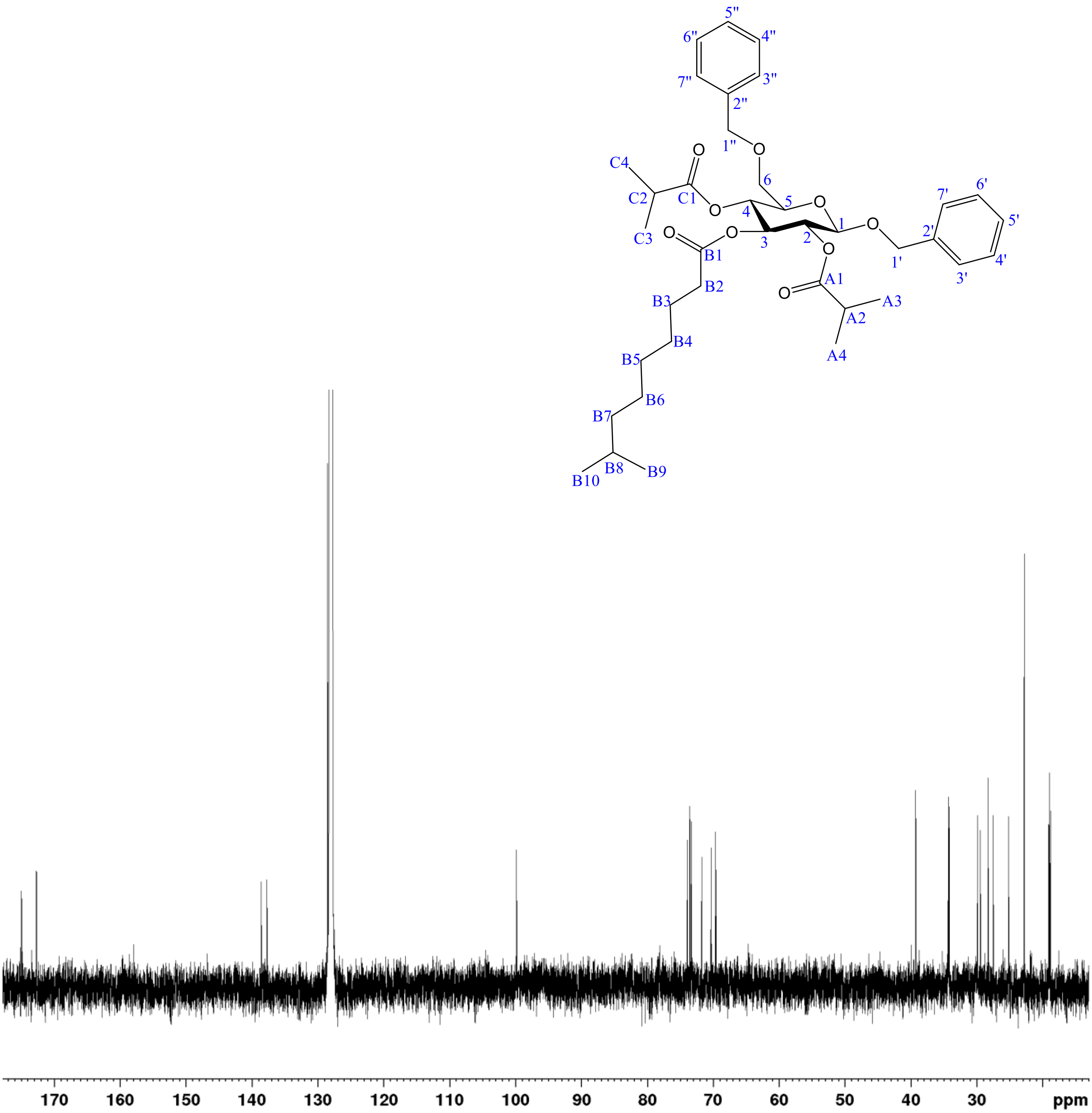

Figure S99: ^{13}C NMR spectrum of compound **17b** (126 MHz, C_6D_6).

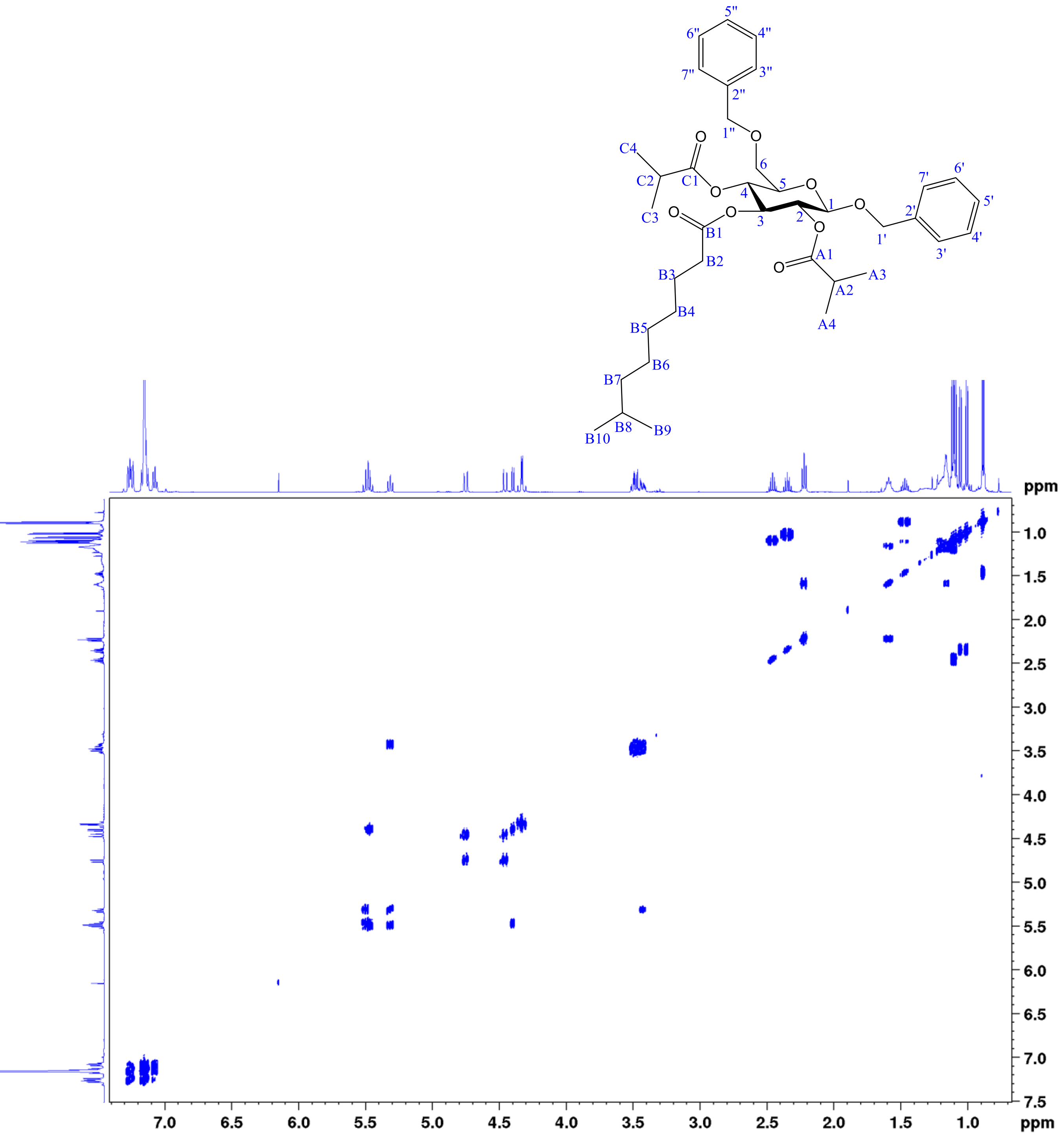

Figure S100: COSY spectrum of compound **17b** (500 MHz, C_6D_6).

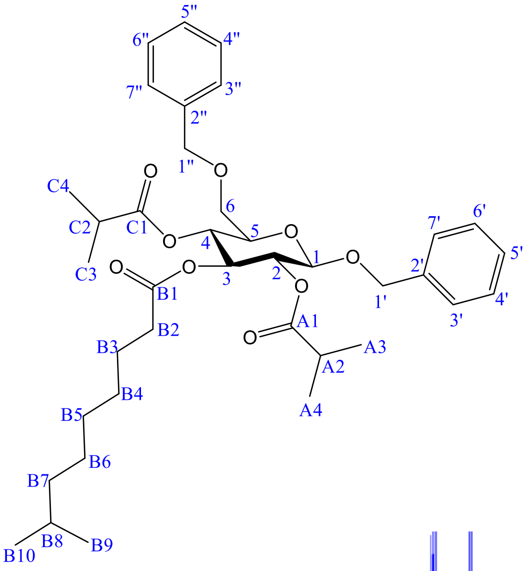

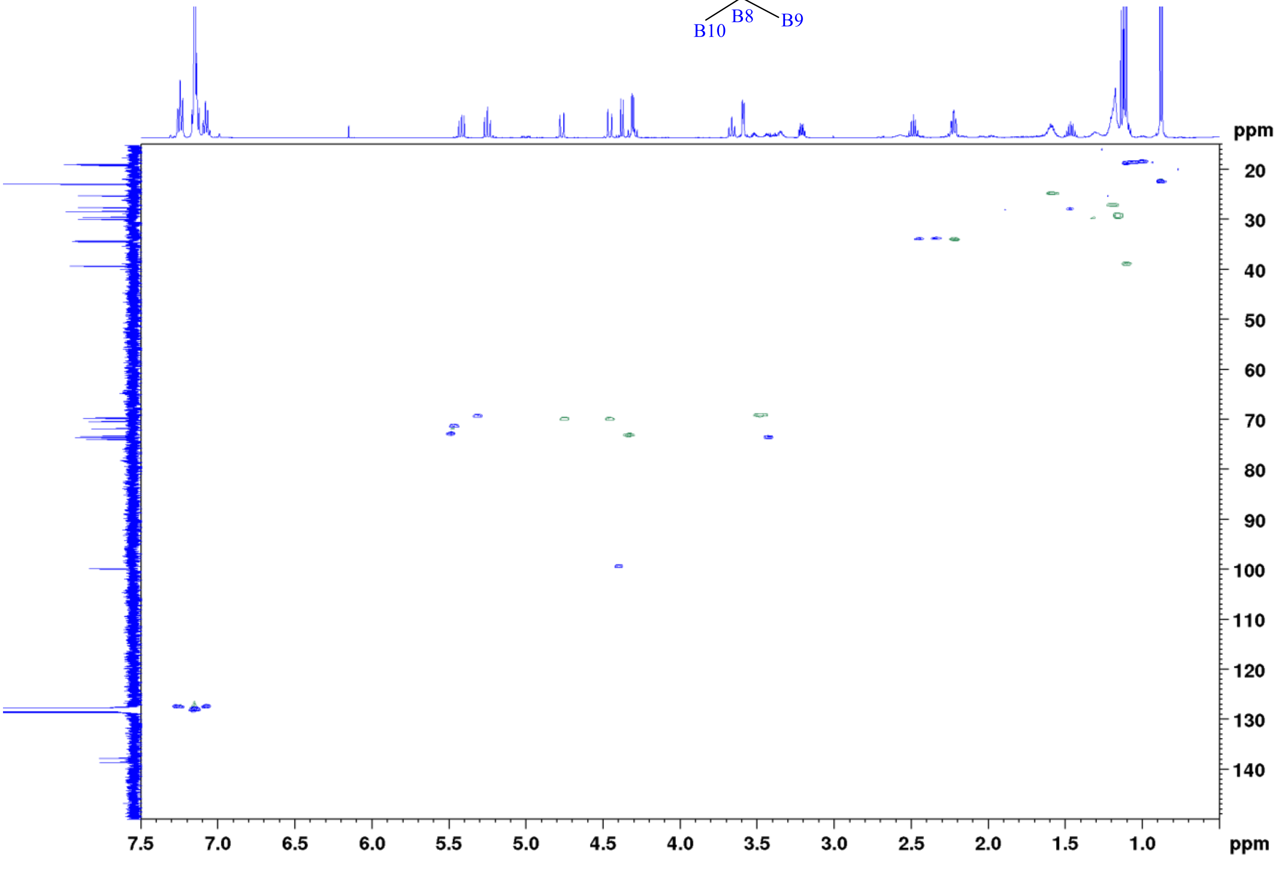

Figure S101: HSQC spectrum of compound **17b** (500 MHz, C_6D_6).

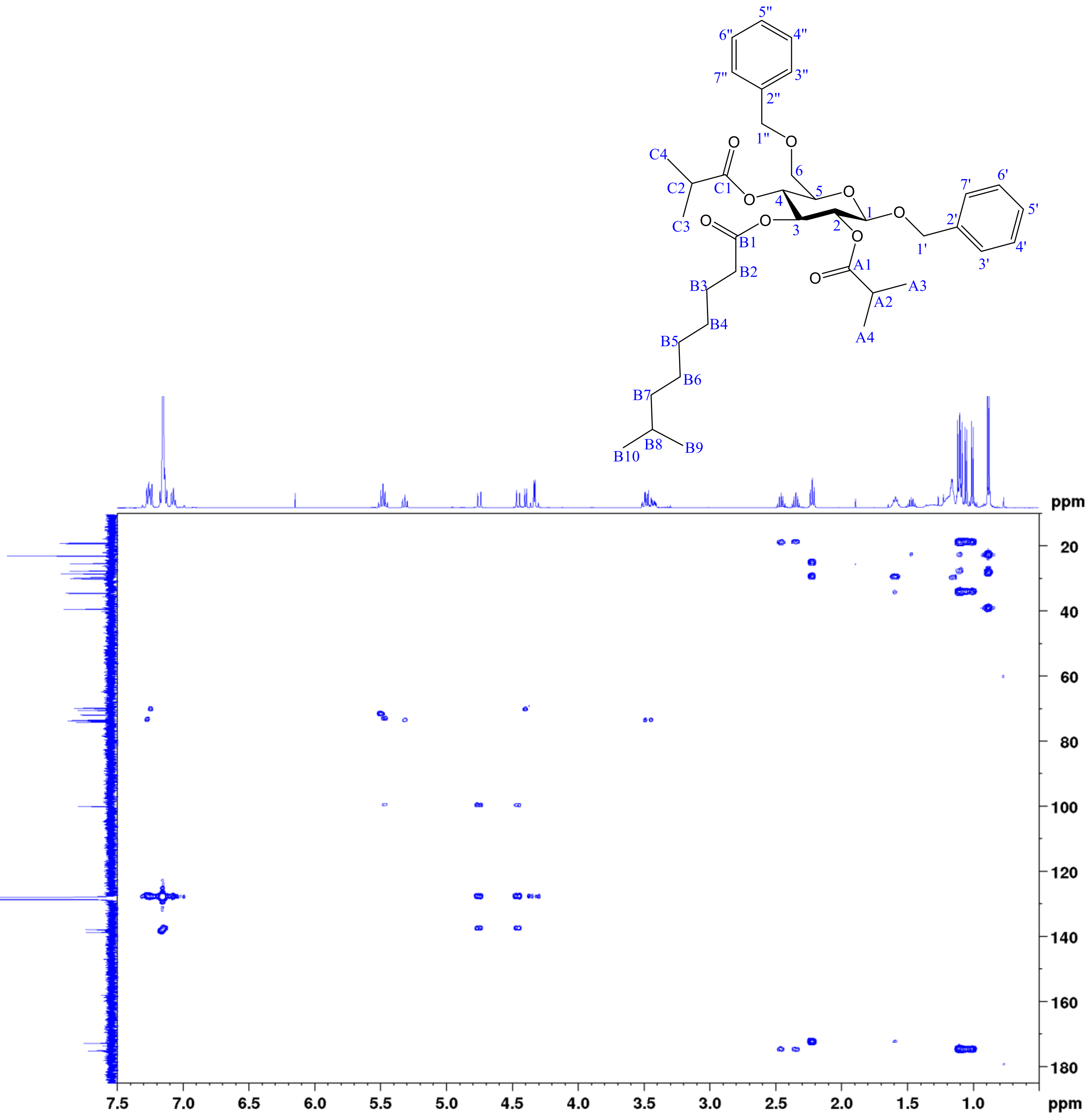

Figure S102: HMBC spectrum of compound **17b** (500 MHz, C_6D_6).
